# Genome-resolved metagenomics of Venice Lagoon surface sediment bacteria reveals high biosynthetic potential and metabolic plasticity as successful strategies in an impacted environment

**DOI:** 10.1007/s42995-023-00192-z

**Published:** 2023-11-03

**Authors:** Elisa Banchi, Erwan Corre, Paola Del Negro, Mauro Celussi, Francesca Malfatti

**Affiliations:** 1 National Institute of Oceanography and Applied Geophysics OGS, Trieste, Italy; 2 FR2424, Station Biologique de Roscoff, Plateforme ABiMS (Analysis and Bioinformatics for Marine Science), Sorbonne Université CNRS, 29680 Roscoff, France; 3 Department of Life Sciences, University of Trieste, Trieste, Italy

**Keywords:** Antibiotic resistance, PAHs, Biosynthetic gene clusters, Microscale, Mobilome, Resistome

## Abstract

Bacteria living in sediments play essential roles in marine ecosystems and deeper insights into the ecology and biogeochemistry of these largely unexplored organisms can be obtained from ‘omics’ approaches. Here, we characterized metagenome-assembled-genomes (MAGs) from the surface sediment microbes of the Venice Lagoon (northern Adriatic Sea) in distinct sub-basins exposed to various natural and anthropogenic pressures. MAGs were explored for biodiversity, major marine metabolic processes, anthropogenic activity-related functions, adaptations at the microscale, and biosynthetic gene clusters. Starting from 126 MAGs, a non-redundant dataset of 58 was compiled, the majority of which (35) belonged to (Alpha- and Gamma-) Proteobacteria. Within the broad microbial metabolic repertoire (including C, N, and S metabolisms) the potential to live without oxygen emerged as one of the most important features. Mixotrophy was also found as a successful lifestyle. Cluster analysis showed that different MAGs encoded the same metabolic patterns (e.g*.*, C fixation, sulfate oxidation) thus suggesting metabolic redundancy. Antibiotic and toxic compounds resistance genes were coupled, a condition that could promote the spreading of these genetic traits. MAGs showed a high biosynthetic potential related to antimicrobial and biotechnological classes and to organism defense and interactions as well as adaptive strategies for micronutrient uptake and cellular detoxification. Our results highlighted that bacteria living in an impacted environment, such as the surface sediments of the Venice Lagoon, may benefit from metabolic plasticity as well as from the synthesis of a wide array of secondary metabolites, promoting ecosystem resilience and stability toward environmental pressures.

**Supplementary Information:**

The online version contains supplementary material available at 10.1007/s42995-023-00192-z.

## Introduction

Microorganisms living in sediments are key players in marine ecosystems at both biogeochemical and ecological levels. Their importance derives in part from the competitive mechanisms and strategies for space and resource utilization, fostered by their high abundance, which may reach 10^9^ cells per cm^3^ (Petro et al. 2017). In upper oceanic sediments, it has been estimated that microbes represent 5 × 10^28^ cells (Flemming and Wuertz 2019). Surface sediments are also complex and dynamic habitats, especially in coastal and lagoon environments, where transport, mixing, deposition, and resuspension concur together with other abiotic (e.g., light, organic matter, oxygen) and biotic factors (Zinger et al. 2011) in shaping microbial assemblages (Banchi et al. 2021; Quero et al. 2017). Indeed, the functioning of these ecosystems is highly dependent on microbial communities, which play essential roles in nutrient cycling, organic matter degradation, and benthic food web dynamics (Schallenberg and Kalff 1993) and can interact and cope with environmental pressures via fundamental strategies. These include metabolic plasticity and functional redundancy: the first refers to the capacity of a community to adapt to environmental changes by tuning the overall performance of the dominant and keystone taxa, the latter implies that different members can perform similar functional roles within the community (Comte et al 2013).

Since most microorganisms (up to 99%; Sharma et al. 2005) are considered unculturable (Stewart 2012), information about their taxonomy and potential functions can only be obtained through DNA-based, culture-independent approaches like metagenomics. These tools have enabled unprecedented exploration of the biodiversity, distribution, dynamics, and ecology of microorganisms in diverse environments (Knight et al. 2017; Semenov 2021). They are also the gold standard in marine ecosystem research, from global- scale expeditions and surveys (Salazar et al. 2016; Sunagawa et al. 2015) to smaller-scale studies and long-term time series analysis (Miksch et al 2021; Yeh and Fuhrman 2022) in both pelagic and benthic realms. Metagenomic reads from shotgun sequencing, in addition to the canonical "assembly-gene prediction-annotation" workflow at the community level (Thomas et al. 2012), have more recently been used for the construction of metagenome-assembled-genomes (MAGs; Tully et al. 2018) (i.e., genome-resolved metagenomics). MAGs enable the linkage of taxonomy, metabolism, and functions, can lead to the discovery of novel species, and can also expand knowledge of microbial processes and interactions by shedding light on the microbial “dark matter” (Setubal 2021; Yang et al. 2021). Although different methods have been implemented for bioinformatic analysis of MAGs, complete (100%) genomes still represent a small fraction of the reconstructed genomes (Chen et al. 2020) due to various issues in sequencing, assembly/binning processes and genome properties. Nevertheless, this approach is an invaluable resource for studying non-culturable microorganisms, especially in under-sampled (Probandt et al. 2017), widespread environments, such as marine sediments. The microbial diversity and functions of the sediment microbes are generally less studied with respect to the pelagic ones, and more efforts are needed also in the framework of ‘omics’ approaches, including genome-resolved metagenomics. For example, in the marine microbial reference databases (https://mmp2.sfb.uit.no; Klemetsen et al. 2018), only 2 of 15 MAGs in MarRef and 1872 of 8,684 MAGs in MARdb, have been reconstructed from sediment samples, whereas the others are related to the water column or host/plant-associated samples.

The Venice Lagoon (northern Adriatic Sea) is an extended and heterogeneous ecosystem subjected to a wide range of natural and anthropogenic pressures including subsidence, tourism, and contaminations (e.g*.*, heavy metals and toxins, accumulated in the sediments after being discharged in the water column) (Depinto et al. 2010; Solidoro et al. 2010). Here, in 2019-2020, we conducted a study in which the sediment prokaryotic communities were characterized with DNA metabarcoding and metagenomics (Banchi et al. 2021). The surface sediment of five sites, distributed in sub-basins of the Lagoon according to the international risk analyses sediment quality guidelines (Apitz et al. 2007) were investigated: Chioggia, Marghera, Palude della Rosa, Sacca Sessola, and Tresse. The results highlighted that the microbial communities were significantly influenced by total organic carbon, salinity and grain size, differentiated among sub-basins (mostly due to the rare microbiome), and more stable compared to pelagic communities over time (Banchi et al. 2021).

To leverage from the previous knowledge, we performed a deep mining of metagenomic data of the surface sediment of the Venice Lagoon to reconstruct MAGs, with the aim of gaining new high-resolution insights on the ecological role of specific microbes. Within this framework, the objectives of this study were to: (i) identify the major metabolic processes linked to the biogeochemical cycles, (ii) investigate the adaptive strategies associated with anthropogenic related activities, biosynthetic gene clusters, and functional adaptation at the microscale, and (iii) evaluate the presence of microbial fundamental properties, such as metabolic plasticity and functional redundancy.

## Materials and methods

### Sampling and metagenomic sequencing

Sampling and sequencing data were published in Banchi et al. (2021). In brief, sampling was conducted seasonally in 2019 at five sites in the Venice Lagoon, Italy (Supplementary Table S1, Supplementary Fig. S1): Chioggia (C), Marghera (M), Palude della Rosa, (P), Sacca Sessola (S), and Tresse (T). Samples were taken in triplicates for each site, for a total of 60 samples. DNA was extracted using the DNeasy PowerSoil Pro kit (Qiagen). Libraries for the 60 metagenomes were prepared according to the Illumina Nextera DNA Flex Library Prep protocol and run on an Illumina NovaSeq 6000 System for a read length of 2 × 250 bp at the Genetic and Epigenetic ARGO Open Lab Platform, Area Science Park, Trieste, Italy.

### Metagenomic assembly and binning

The number and quality of metagenomic reads were checked with FastQC (Andrews 2010). Reads were cleaned with Trimmomatic (Bolger et al. 2014) and assembled into contigs using MEGAHIT v1.2.9 (Li et al. 2015). In order to maximize the output of the binning process, we co-assembled the metagenomes in two different ways for each site: using the three replicates of the same season (e.g*.*, the three Spring metagenomes from Chioggia) or using one replicate for each season (e.g*.*, one Spring, one Summer, one Autumn and one Winter from Chioggia). This procedure enabled the construction of 25 assemblies, five for each sampling site.

Assemblies were performed combining either three replicates of each site for the same season or one replicate for each season for each site, for a total of 25 assemblies for the 5 sites. Reads were mapped back to assembled contigs > 1000 bp using Bowtie2 v.2.4.1 (Langmead and Salzberg 2012) with default parameters.

Genomic binning was performed with CONCOCT v1.1.0 (Alneberg et al. 2014), MaxBin2 v.2.2.6 (Wu et al. 2014), and MetaBAT2 v2.14 (Kang et al. 2019). Bins obtained from the three algorithms were integrated using a consensus binning strategy with DAS_Tool v1.1.3 (Sieber et al. 2018) and potentially contaminating contigs (presenting divergent GC content, tetranucleotide signature, coverage, taxonomy) were removed with RefineM v0.1.2 (Parks et al. 2017). Bins completeness and contamination were assessed through single-copy marker gene analysis using CheckM v1.1.11 (Parks et al. 2015). Bins with > 90% completeness and < 5% contamination were considered high-quality MAGs, while bins with > 50% completeness and < 10% contamination were considered medium-quality MAGs (Bowers et al. 2017; Konstantinidis et al. 2017).

### MAGs taxonomic assignment and functional annotation

The taxonomy of each MAG was assigned with GTDB-Tk v1.7.0 (Chaumeil et al. 2020) on the GTDB v.R06-RS202 (Parks et al. 2018) database and with the microbial genome atlas (MiGA, NCBI-Prok Database; Rodriguez et al. 2018). Taxonomic assignment was further compared with 16S rRNA gene amplicon sequence data from the same samples (Banchi et al. 2021) annotated with SILVA v. 138 (Quast et al. 2013). A phylogenetic tree was constructed based on 43 conserved single-copy, protein-coding marker genes (Kato et al. 2018; Parks et al. 2015) using the maximum likelihood algorithm with MEGAX (Kumar et al. 2018) with default parameters. The tree was visualized and edited with Interactive Tree Of Life (iTOL) v5 (Letunic and Bork 2021).

Then, all reconstructed MAGs from the 25 assemblies (5 per sampling site) were pooled together and dereplicated at the strain level using dRep (Olm et al. 2017) (v.3.2.2; parameters: -p 72 --ignoreGenomeQuality -pa 0.95 -sa 0.99 -cm larger, following Xie et al. 2021). A Sankey diagram was constructed with the *networkd3* package (https://github.com/christophergandrud/networkD3) in the R environment (v. 4.2.1, R Core Team 2019).

The coverage percentage and relative abundance of the dereplicated MAGs in each assembly was computed by read mapping with CoverM v.0.6.1 (https://github.com/wwood/CoverM). A MAG was considered present in a sample if the coverage was > 80% (Zhou et al. 2020). Significant differences of MAG abundance among sites were assessed with Kruskal-Wallis test and Wilcoxon non-paired. False discovery rate (FDR) was used for p-value correction and results with q-value < 0.05 were considered significant.

Genes were predicted with Prodigal v.2.6.3 (Hyatt et al. 2010) and functional annotations were performed with the SEED Subsystem (Overbeek et al. 2005) using RASTtk at default parameters (Aziz et al. 2008, Brettin et al. 2015) and with KAAS (KEGG Automatic Annotation Server; Moriya et al. 2007) with GHOSTX settings. SEED annotations were used to assess the presence and distribution of genes related to anthropogenic- and microscale-related functions. KEGG annotations were screened to infer potential metabolisms of the MAGs using key marker genes related to central, carbon, methane, nitrogen, hydrogen, and sulfur metabolism (Acinas et al. 2021; Dombrowski et al. 2018). A hierarchical cluster analysis with the Ward method (Ward.D2) was performed in R (v. 4.2.1, R Core Team 2019) at gene and metabolism level. The relative contribution of key genes and metabolism to the average Bray-Curtis dissimilarity between each cluster *vs* the other clusters was calculated using a one-way similarity percentage procedure (SIMPER, cut-off: 50%) with the R package *vegan* (Oksanen et al. 2019). For high-quality MAGs, KEGG annotations were used to assess the completeness of the key metabolic pathway modules using the Reconstruction Tool of KEGG Mapper (Kanehisa and Sato 2020). Biosynthetic gene clusters (BGCs) were detected and identified within each MAG using Antibiotics and Secondary Metabolite Analysis Shell (antiSMASH) v.7 (Blin et al. 2021) at the default parameters. A hierarchical cluster analysis with the Ward method (Ward.D2) was performed in R (v. 4.2.1, R Core Team 2019) considering all the results from the different annotation approaches in terms of presence/absence.

## Results and discussion

### Metagenomic binning and MAG dataset

Shotgun sequencing produced ~120 millions of raw reads, with 14.8 ± 3.7 millions of paired-end reads of 35 ± 2 quality score for each metagenome on average. The number of contigs > 1000 bp derived from the three co-assembled metagenomes for each sample were 5,208,341 ± 376,210.

**Fig. 1:**
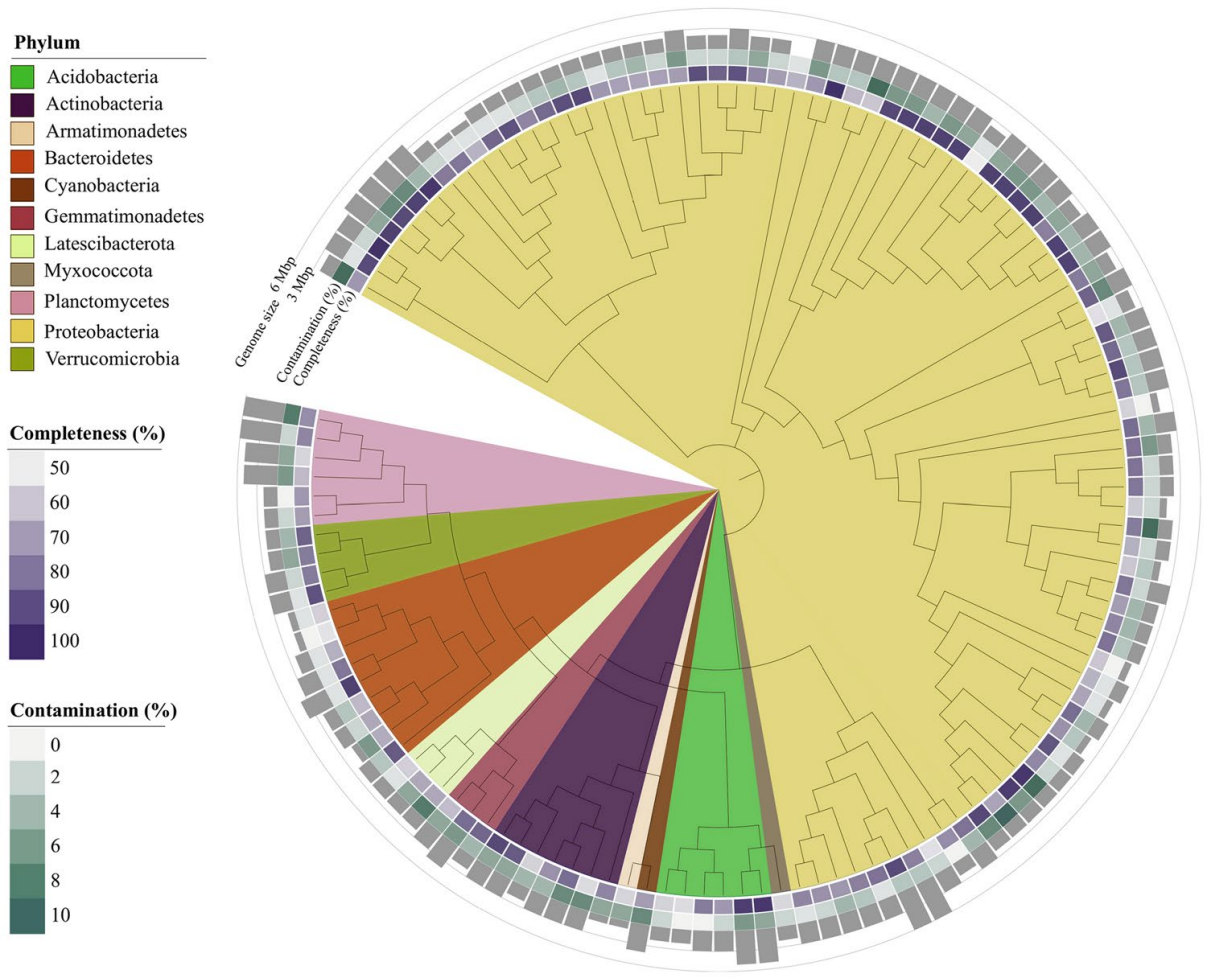
Phylogenetic tree of MAGs reconstructed from Venice Lagoon sediment, based on a concatenated alignment of 43 conserved marker genes. Clade colours indicate taxonomic assignment at the phylum level. MAGs completeness (%), contamination (%), and size (Mbp) are shown in the concentric rings outside the tree

The binning procedure allowed reconstructing 339 bins from the 25 assemblies (5 per each sampling site), 126 of which were considered MAGs (Fig. 1, Supplementary Table S2, Supplementary Table S3). The MAGs average size was 3.07 ± 1.06 Mbp, with 77.0 ± 12.8 % completeness, 3.9 ± 2.3 % contamination, and 21.4 ± 19.1 % of strain heterogeneity (Fig. 1, Supplementary Table S3). Regarding the binning algorithms, Metabat was the most successful: 92 MAGs resulted from the selection/dereplication of the bins performed with Metabat, 34 with Concoct, and none with Maxbin (Supplementary Table S3). Of the 126 MAGs, 14 were of high quality and 112 of medium quality (Supplementary Table S2, Supplementary Table S3). The dereplication procedure enabled constructing a dataset of 58 non-redundant MAGs, 9 of which were of high quality (Supplementary Table S4). These numbers are in line with other studies on marine sediment with a comparable set of samples and sequencing effort in both marine sediment (Zhang et al. 2019; Zhao et al. 2020) and water (Haro-Moreno et al. 2018; Kimbrel et al. 2018; Trivedi et al. 2020).

Different factors may affect the number and quality of reconstructed genomes, which can be considered for an overview of the metagenome binning process. The reconstruction of MAGs is likely limited by strain heterogeneity (i.e*.*, microdiversity), which reduces the assembly quality and thus the efficiency of binning (Meziti et al. 2021; Ramos-Barbero et al. 2019). In addition, the most abundant microorganisms in the community are often not resolved at genome level, which reduces the number of reconstructed MAGs; this phenomenon is known as “The great metagenomic anomaly” (Okazaki et al. 2022; Ramos-Barbero et al. 2019). Dong and colleagues (2022), in an analysis of sand sediments associated with coral reefs, considered that the strain heterogeneity they calculated, 40% on average, could affect MAGs recovery. In our dataset, this value averages 21% (Supplementary Table S2, Supplementary Table S3), thus not representing a limitation for genomes’ reconstruction.

The coverage of the non-redundant MAGs across the assemblies revealed that 98.4 ± 1.6 % of reads were mapped on average (Supplementary Table S5), indicating that all MAGs were present at all sites. The relative abundance of MAGs in each assembly ranged from 0.002 to 3.5 %, and 37 MAGs presented a significant different relative abundance at site level (Supplementary Table S6). In particular, 16 MAGs were significantly higher in Sacca Sessola than in the other sites. As we mapped all MAGs at all five sites in the Venice Lagoon in all seasons (even if some presented significant differences in terms of abundance), this implies that the same genomes were reconstructed in all the samples.

Based on the metagenomic data obtained from different Lagoon areas characterized by different anthropogenic pressures and sediment features, one might expect some degree of differentiation in time and space. On the other hand, the environmental gradients present in our samples were not as high as in other studies (Acinas et al. 2021; Dong et al. 2022). Moreover, in previous investigations (Banchi et al. 2021), we saw that sites had no or minimal proportions of "private taxa" and that differences among sites were mainly due to differences in relative abundance. Knowing the temporal stability of the microbial communities, the use of all co-assemblies from the different seasons and of the combined seasons was considered to maximize the binning process leading to a higher number of genomes of good quality, instead of increasing the genome diversity.

### MAGs taxonomic composition

**Fig. 2:**
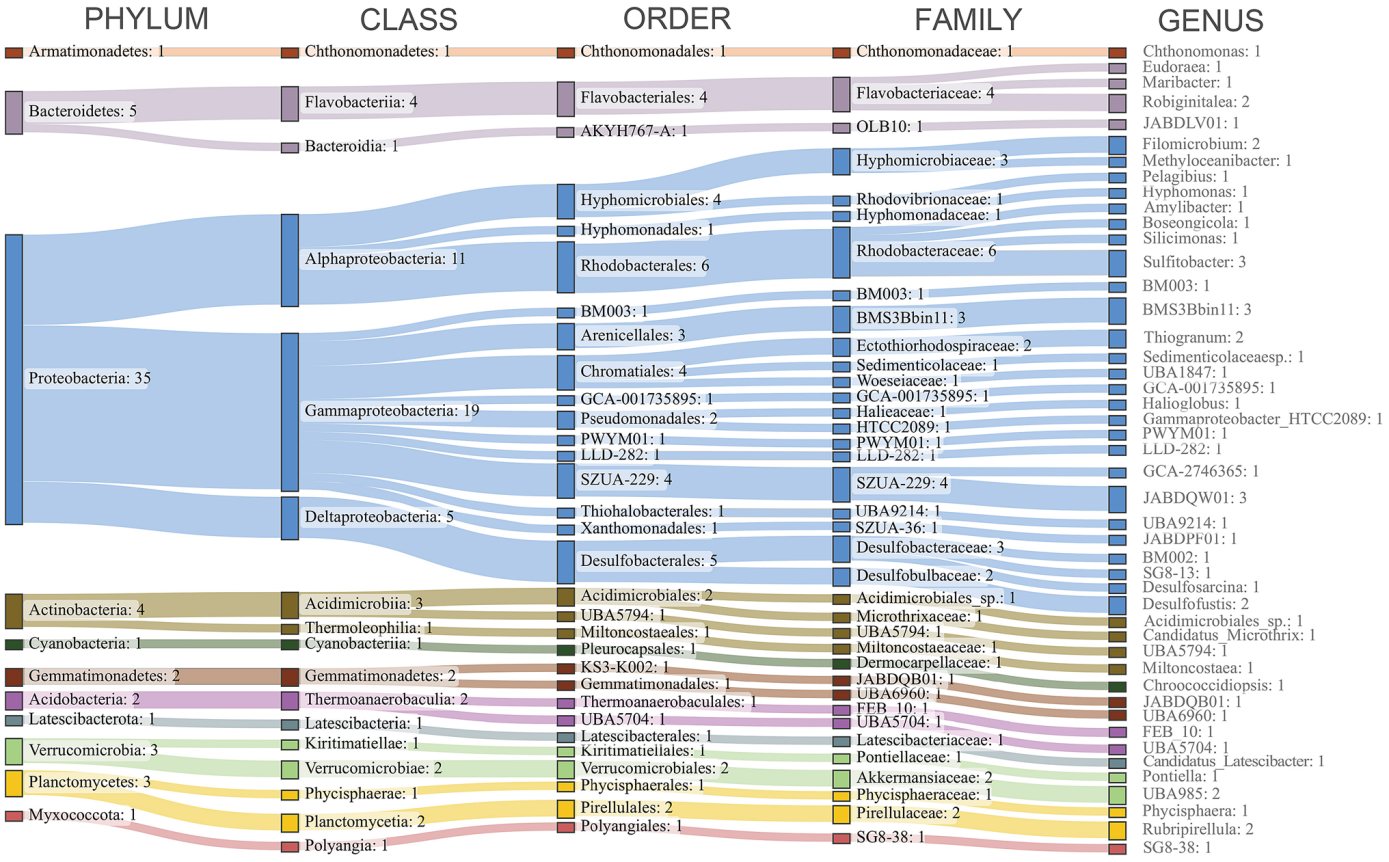
Sankey diagram showing the taxonomic assignment up to the genus level of the 58 non-redundant MAGs reconstructed from Venice Lagoon sediment

The high diversity of the benthic microbial communities (compared to their seawater counterpart) is well known and mostly attributed to the complexity and dynamics of this environment, that may be coupled with steep physicochemical gradients (Acosta-González and Marques 2016; Zinger et al. 2011). The non-redundant MAG dataset spanned a wide range of phyla and classes (11 and 17, respectively, Fig. 2). Of the 58 MAGs, 55 were identified at genus level, and 29 at species level. The majority (35) belonged to the phylum Proteobacteria (Fig. 2, Supplementary Table S4). At the class level, Gammaproteobacteria were the most abundant (20), followed by Alphaproteobacteria (11). The prevalence of Gammaproteobacteria agreed with the data we obtained through 16S metabarcoding for the same sites (Banchi et al. 2021) and is consistent with what is commonly found in marine sediments and in other studies in the Venice Lagoon (Borin et al. 2009, Quero et al. 2017). At the order and family level, Rhodobacterales and Rhodobacteraceae were the most abundant (6). Within genera, the most present (3 MAGs each) were BMS3Bbin11 (Arenicellales), JABDQW01 (SZUA-229, uncultured Gammaproteobacteria), and *Sulfitobacter*.

The MAG belonging to the genus *UBA1847*, already found in coastal sediments (Seidel et al. 2021), belongs to the Woeseiaceae family, which are ubiquitous and abundant bacteria in coastal sediments (Mußmann et al. 2017). In relation to the low oxygen levels found in Venice Lagoon sediment (Borin et al. 2009), we reconstructed different genomes belonging to sulfur-reducing bacteria (Deltaproteobacteria, Desulfobacterales). These included the Desulfobacteraceae *BM002 sp. 002899795, SG8-13, Desulfosarcina,* and the Desulfobulbaceae *Desulfofustis*. Other sulfur-related bacteria in our dataset belonged to the genus *Thiogranum* (*Thiogranum sp.015494295*), considered an obligately chemolithoautotrophic, sulfur-oxidizing taxon (Mori et al. 2015), and *Filomicrobium* (*Filomicrobium sp.00151606*) a methanesulfonate degrading bacteria (King 2018). We detected a member of the genus *Methyloceanibacter*, a taxon commonly found in sediment and that includes methylotrophs and methanotrophs (King 2018). Marine methylotrophs are key players in the biogeochemical carbon cycle, metabolizing reduced one-carbon compounds (e.g., methane) which in marine sediments (including Venice Lagoon; Zonta et al. 2020) are usually present at high concentration (Vekeman et al. 2016).

The comparison of MAGs’ GTDB (Chaumeil et al. 2020)/MiGA (Rodriguez et al. 2018) taxonomic assignment with 16S rRNA-derived taxonomy, besides the difference in data production (shotgun sequencing *vs* amplicon) and processing (genome-level assignment *vs* barcode region), represents an additional resource to explore our data. At the genus level, all “defined” genera (*Amylibacter, Boseongicola, Candidatus Microthrix, Candidatus Latescibacterota, Chroococcidiopsis, Chthonomonas, Desulfofustis, Desulfosarcina, Eudoraea, Filomicrobium, Halioglobus, Hyphomonas, Maribacter, Methyloceanibacter, Pelagibius, Phycisphaera, Robiginitalea, Rubripirellula, Silicimonas, Sulfitobacter, Thiogranum*) were present except for *Pontiella* and *Miltoncostaea*, which are not included in the SILVA database (Quast et al. 2013). The majority of MAGs belonged to the most abundant (> 0.1%, relative abundance, on average; with *Candidatus Latescibacterota* and *Halioglobus* in the top ten genera, > 1% on average) genera following 16S data (Banchi et al. 2021) and may be considered members of the prokaryotic core community (Probandt et al. 2018).

Although Archaea were present in our sediment samples (i.e*.*, they accounted for approximately 4% of the total 16S rRNA reads; Banchi et al. 2021), we were unable to reconstruct any archaeal genome. Typically, due in part to their low abundance in the marine environment, the number of archaeal MAGs is a small fraction of the total MAGs, generally ranging from 0 to 6% (Acinas et al. 2021; Dong et al. 2022; Trivedi et al. 2020; Vavourakis et al. 2018; Zhang et al. 2019) of the whole dataset. Moreover, archaeal genomes are still poorly studied compared to their bacterial counterparts, and this is also reflected in genome-resolved bioinformatics analyses (e.g*.*, fewer representatives in reference databases, higher divergence even at higher taxonomic levels), which may bias the binning process (Gribaldo and Brochier-Armanet 2006; Nasir et al. 2014; Vollmers et al. 2022).

### MAGs metabolic pathways, plasticity, and redundancy

Marine sediments microbial communities are hot spots of element cycling and represent the major carbon sink on our planet (Mußmann et al. 2017). Genome-resolved metagenomics has the capacity to provide significant insights into the benthic microbial community, as it allows discovering new metabolic pathways and a deeper understanding of the structure and function of microbial coupling (Ward et al. 2020).

**Fig. 3:**
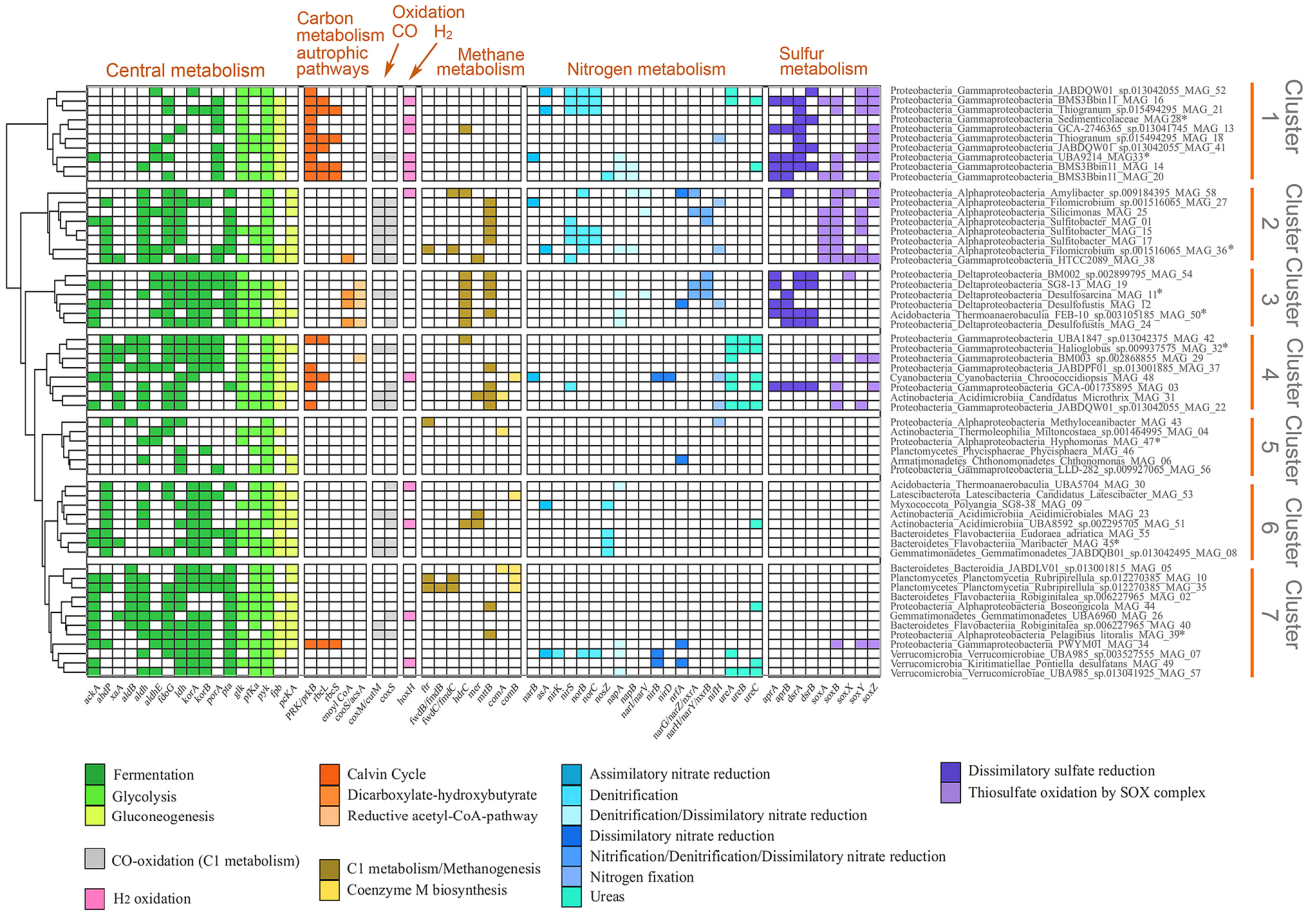
Heatmap showing the metabolic potential of the MAGs based on the presence of key genes and metabolic pathways. enoyl-Coa: enoyl-CoA hydratase/3-hydroxyacyl-CoA dehydrogenase; Dicarboxylate-hydroxybutyrate: dicarboxylate-hydroxybutyrate cycle/hydroxypropionate-hydroxybutylate cycle; Reductive acetyl-CoA pathway: reductive acetyl-CoA pathway (Wood-Ljungdahl pathway). Asterisks indicate high-quality MAGs

The reconstructed genomes of this study were screened for metabolic potential and plasticity based on the presence and distribution of selected marker genes related to the main biogeochemical cycles and metabolisms (Acinas et al. 2021; Dombrowski et al. 2018). These included the KEGG categories of Central metabolisms, Carbon metabolism/autotrophic pathways, CO oxidation, H_2_ oxidation, Methane metabolism, Methane production, Nitrogen/Methene metabolism, Nitrogen metabolism, and Sulfur metabolism. Of the 103 marker genes, described in Acinas et al. (2021) and Dombrowski et al. (2018), 61 were found in the MAG dataset (Fig. 3, Supplementary Table S7), revealing a wide metabolic repertoire.

Within Central metabolism, we identified marker genes related to three different pathways: fermentation, glycolysis, and gluconeogenesis. Central metabolism key genes were the most present within our dataset: fermentation and glycolysis key genes were present in every MAG (58), while Gluconeogenesis genes were found in 48 MAGs. Besides being able to process organic carbon and biomass via fermentation, in 22 MAGs we detected key genes of Carbon metabolism/autotrophic pathways for Carbon fixation: Calvin cycle, Dicarboxylate-hydroxybutyrate cycle/Hydroxypropionate-hydroxybutylate cycle, and Wood-Ljungdahl pathway. CO Oxidation key marker genes were present in 22 MAGs, whereas the H_2_ Oxidation key gene was found in 12 MAGs. Methane metabolism marker genes, related to C1 metabolism/methanogenesis and Coenzyme M biosynthesis, were found in 27 MAGs. Within Nitrogen metabolism, in 40 MAGs we identified key genes, related to Assimilatory nitrate reduction, Nitrification/Denitrification/Dissimilatory nitrate reduction, and Nitrogen fixation. Sulfur metabolism-related genes, belonging to Dissimilatory sulfate reduction and Thiosulfate oxidation by SOX complex, were detected in 28 MAGs (Fig. 3).

Fermentation represented the microbial core function present in all phyla of benthic communities (Dombrowski et al. 2018), even in our dataset (Fig. 3). Fermentation, despite having a low yield of ATP in comparison to aerobic respiration 2 *vs* 38 respectively (Buckel et al. 2021), occurs in anoxic conditions where there is a lack of oxygen and no need for external electron acceptors. This implies that all the genomes we have reconstructed are able to survive in the absence of oxygen, a frequent condition in lagoon sediments. When computing all the contribution of the autotrophic metabolisms such as Carbon (*PRK/prkB, rbcL, rbcS, enoyl-CoA hydratase*/3-*hydroxyacyl-CoA dehydrogenase*, *cooS/csA*), CO (*coxM/cutM* and *coxS*) and H_2_ oxidation (*hoxH*), mixotrophy was revealed as a widespread successful strategy among the majority of the Proteobacteria members.

In our MAG dataset, within the methane metabolism, we detected the presence of the *mttB* gene in all (Alphaproteobacteria) Rhodobacteraceae, the presence of *mttB* and *hdrC* in all (Deltaproteobacteria) Desulfobacteraceae, and the presence of *hdrC* in all (Deltaproteobacteria) Desulfobulbaceae (Fig. 3). The gene *mttB* encodes for a trimethylamine methyltransferase, suggesting a capacity for anaerobic one-carbon metabolism (Lin et al. 2021), and it is commonly found in pelagic and benthic marine Rhodobacteraceae (Kanukollu et al. 2016; Simon et al. 2017) and in Desulfobacteraceae (Lin et al. 2021). The gene *hdrC* encodes for a heterodisulfide reductase and is considered essential in oxidative sulfur metabolism, and therefore present in sulfur-oxidizing prokaryotes (Boughanemi et al. 2016). In the context of the sulfur cycle, we identified bacteria that encode genes related to sulfate reduction (*aprA, aprB, dsrA, dsrB*) and thiosulfate oxidation (*soxA, soxB, soxX, soxY, soxZ*) (Fig. 3). These include taxa already known to be involved in these processes such as *Thiogranum sp.* (Mori et al. 2015) and *Filomicrobium* sp. (King 2018), *Amylibacter sp.*, HTTC2089 (González et al. 2019), *BMS3Bbin11 sp.* (Kato et al. 2018), SZUA-229 (Zhong et al. 2022), *FEB*-*10* (recently proposed to be included as *Sulfomarinibacter* in the new family Sulfomarinibacteraceae; Flieder et al. 2021), GCA-001735895 (Chen et al. 2021) as well as other bacterial taxa not previously recognized as being involved in this metabolism (e.g., PWYM01).

The stability of an ecosystem can be enhanced by metabolic plasticity, in which organisms can switch between different metabolisms and pathways to cope with environmental changes, the availability of nutrient and energy sources (Biggs et al. 2020). In our dataset, metabolic plasticity was observed in several members of Alpha- and Gamma-proteobacteria, which potentially can use diverse electron donors such as H_2_ and reduced species coupled with electron acceptors such as O_2_ and nitrate and nitrite. Such plasticity was reported for communities found in hydrothermal vent systems (Anantharaman et al. 2013).

The hierarchical clustering of MAGs based on key genes, grouped the genomes into 7 clusters (Fig. 3), indicating potential functional redundancy among specific taxa. We ran SIMPER (Supplementary Table S8) to identify the presence of characterizing genes of each cluster. Even if each of the identified clusters contains at least a high-quality MAG (i.e., >90 % completeness; Fig. 3) which showed representative sets of characterizing genes, we are aware that in some cases the lack of these genes may be the consequence of the lower genome completeness. For this reason, we focused the discussion of the ‘present’ genes with respect to the missing ones, considering the cluster analysis an efficient approach to highlight distinctive patterns. Cluster 1 included 10 MAGs, Gammaproteobacteria belonging to Arenicellales, Chromatiales, SZUA-229, and Thiohalobacterales. Cluster 1 characterizing genes were: *porA* (Fermentation; Central metabolism), *PRK/prkB* and *rbcL* (Calvin cycle; Carbon metabolism/autotrophic pathways), *soxY* and *soxZ* (Thiosulfate oxidation; Sulfate metabolism). Cluster 2 clustered 7 Alphaproteobacteria (Rhodobacterales and Hyphomicrobiales) and one Gammaproteobacteria (Pseudomonadales). The genes present in Cluster 2 that contributed significantly to the differentiation from the others were: *mttB* (Methanogenesis; Methane metabolism), *coxM/cutM* and *coxS* (CO oxidation; C1 metabolism), *nirS* (Denitrification; Nitrogen metabolism), *soxA*, *soxB* and *soxY* (Thiosulfate oxidation; Sulfate metabolism). Cluster 3 grouped all the 6 Deltaproteobacteria (Desulfobacterales) and one Acidobacteria (Thermoanaerobaculales). The characterizing genes in Cluster 3 were: *hdrC* (Methanogenesis; Methane metabolism), *cooS/acsA* (Wood-Ljungdahl pathway; Carbon metabolism/autotrophic pathways), *napA* (Denitrification/Dissimilatory Nitrate reduction; Nitrogen metabolism), *aprA*, *aprB*, *dsrA*, *dsrB* (Dissimilatory sulfate reduction; Sulfur metabolism). Cluster 4 included 8 genomes: 6 Gammaproteobacteria, one Actinobacteria (Acidimicrobiales) and the Cyanobacteria (Pleurocapsales). Cluster 4 characterizing genes were: *ureA* and *ureC* (Urease; Nitrogen metabolism), *exaA* (Fermentation/alcohol dehydrogenase; Central metabolism), *PRK/prkB* (Calvin cycle; Carbon metabolism/autotrophic pathways), and *mttB* (Methanogenesis; Methane metabolism) (Fig. 3; Supplementary Table S8). Cluster 5 was formed by 6 genomes belonging to different phyla (Actinobacteria, Armatimonadetes, Planctomycetes and Proteobacteria). The cluster was characterized by the presence of genes related to Central Metabolism, and from the depletion of the other pathways. Accordingly, the SIMPER analysis (Fig. 3; Supplementary Table S8) did not identify any characterizing gene. Cluster 6 grouped 8 genomes belonging to non-Proteobacteria phyla (Actinobacteria, Bacteroidetes, Gemmatimonadetes, Latescibacterota and Myxococcota). The genes present in this cluster which significantly contributed to differentiate it from the others were: *nosZ* (Denitrification; Nitrogen metabolism), *coxS* (CO oxidation; C1 metabolism), and *adhP*, *korA* and *korB* (Fermentation; Central metabolism) (Fig. 3; Supplementary Table S8). Finally, Cluster 7 was formed by 12 genomes including all the MAGs belonging to Verrucomicrobia. The characterizing genes of this cluster belonged to Central metabolism: *ackA*, *aldB*, *pta* (Fermentation) and pckA (Gluconeogenesis) (Fig. 3; Supplementary Table S8).

Functional redundancy (i.e*.*, taxa performing similar functions) is thought to promote the resilience of biological communities by increasing the buffering capacity in response to the loss of individuals and therefore maintaining the functioning of the ecosystem (Biggs et al. 2020; Pan et al. 2022). We detected different bacteria encoding the same metabolic pathways (Fig. 3), suggesting the presence of functionally redundant microbes. For instance, all the members of Cluster 1 were potentially capable of carbon fixation via the Calvin cycle, representatives of Cluster 2 oxidized CO. Bacteria within Cluster 1 and Cluster 3 performed dissimilarity sulfate reduction, while members of Cluster 1 and 2 were potentially capable of thiosulfate oxidation. As previously noted for metabolic plasticity, functional redundancy features were also found primarily in Alpha- and Gamma-proteobacteria, supporting the hypothesis of key roles of these organisms in lagoon’s ecosystem functioning. The presence of multiple survival strategies could enhance microbial adaptation to environmental changes, as well as the exploitation of different ecological niches (Dombrowski et al. 2018; Pan et al. 2022) of the heterogeneous surface sediment habitat of the Venice Lagoon.

The assessment of the module completeness of the selected metabolic pathways in the 9 high-quality MAGs (Supplementary Table S9) could be determined for Central metabolism (Glycolysis, Gluconeogenesis), Carbon metabolism/autotrophic pathways (Calvin cycle, Dicarboxylate-hydroxybutyrate cycle/Hydroxypropionate-hydroxybutylate cycle, and Wood-Ljungdahl pathway), Methane metabolism (Coenzyme M biosynthesis, C1 metabolism/Methanogenesis), Nitrogen metabolism (Assimilatory nitrate reduction, Denitrification, Dissimilatory nitrate reduction, Nitrogen fixation) and Sulfur metabolism (Dissimilatory sulfate reduction, Thiosulfate oxidation by SOX complex). The high-quality MAGs presented complete or almost complete (up to 2 missing blocks) modules related to Central metabolism (Supplementary Table S9). While in four MAGs (MAG 32,29,45,47) these were the only complete modules, four (MAG 11,33,36,50) presented complete modules relative to the Nitrogen metabolism, and five (MAG 11,28,33,36,50) to the Sulfur Metabolism. Noteworthy, no high-quality MAG presented complete Methane-related modules (Fig. 3).

### Anthropogenic activity-related functions

**Fig. 4:**
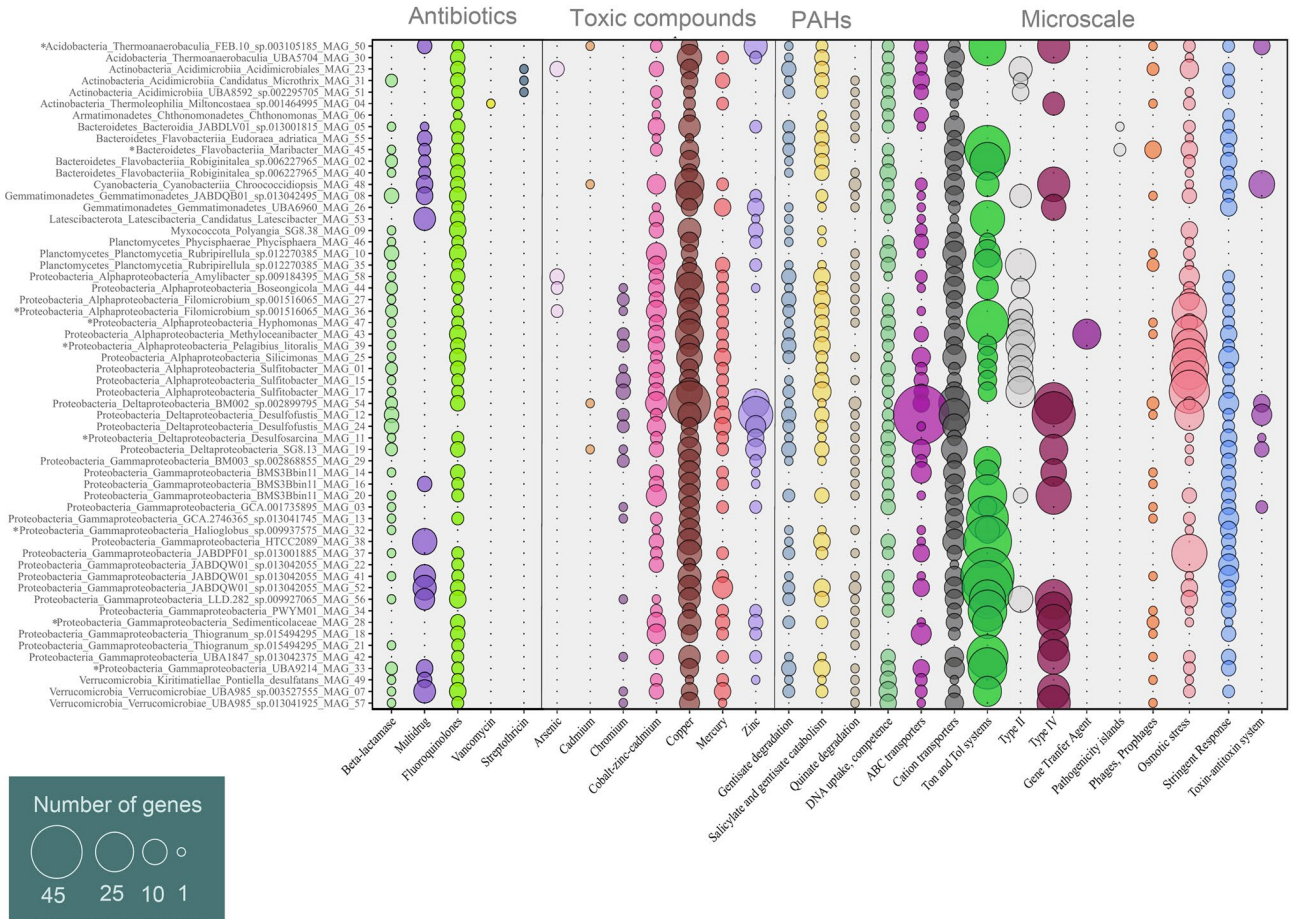
Presence and abundance of genes coding for anthropogenic- and microscale-related functions identified in the MAG dataset. Asterisks indicate high-quality MAGs. PAH: polycyclic aromatic hydrocarbons

The impact of human-related activities requires multidisciplinary and integrated management strategies to assess, predict and mitigate their effect on valuable, productive, and vulnerable coastal and transitional ecosystems like the Venice Lagoon. The genomes we reconstructed were investigated for the presence and abundance of genes related to the resistance to antibiotics, to the resistance to toxic compounds and to the degradation of aromatic compounds (Fig. 4).

Marine sediments are considered reservoirs of genes bearing resistance to antibiotics and toxic compounds (e.g*.*, heavy metals), representing a pool that can contribute to their spreading, transfer, and evolution (Vignaroli et al. 2018; Wu et al. 2021; Yang et al. 2013). Furthermore, resistance genes from contaminated sediment can be mobilized and resuspended in the overlaying water, posing a health risk particularly in coastal areas (Heß et al. 2018). In our dataset, all MAGs presented genes related to the resistance to antibiotics or toxic compounds, indicating that Venice Lagoon sediments represent a long-standing impacted environment. Microbes are therefore constantly challenged and need to cope with human-derived compounds from runoff waters, sewage, farms, tourism, traffic and industry.

Antibiotic resistance is a major health issue at a global level, threatening humans, animals, and the environment (Kim and Cha 2021; Larsson and Flach 2022). It is a critical concern within the One Health framework, which highlights the interconnections and interdependencies among these three domains (Aslam et al. 2021). The most widespread antibiotic resistance genes in the MAGs were the ones toward Fluoroquinolones (present in 51 MAGs), followed by Beta-lactamase (39), and Multidrug (17) (Fig. 4). Following Currant and coauthors (2022), we underline that Fluoroquinolone resistance was identified by the detection of *DNA gyrase* (topoisomerases) genes, which are not conclusive of the presence but only of the potential of resistance variants. The genes of the MAG dataset that were associated with antibiotic resistance represented a subset of the categories annotated in metagenomes (Banchi et al. 2021), suggesting that these genetic traits were not homogeneously distributed across prokaryotic communities, as previously reported (Wang et al. 2019; Yang et al. 2022). While Beta-lactamase and Fluoroquinolones resistance were distributed across all phyla, Streptotricin resistance was recorded only within (Actinobacteria) Acidimiicrobia and Vancomycin resistance only in (Actinobacteria) Thermoleophilia (Fig. 4).

Heavy metal pollution is one of the most significant sources of environmental contamination (Roane and Kellogg 1996) and represents a long-term selection pressure on microbial communities (Stepanauskas et al. 2005). Moreover, toxic compounds, including heavy metals, can support and co-select antibiotic resistance through different mechanisms, such as co-resistance, cross-resistance, and co-regulation (Baker-Austin et al. 2006; Gupta et al. 2022; Vats et al. 2022), which may lead to greater diversity and abundance of antibiotic resistance genes in the microbial communities (Qi et al. 2021). The genes related to toxic compounds detected in the MAGs included all categories previously found in the metagenomic dataset (Banchi et al. 2021), which may indicate a higher prevalence of these features compared to antibiotic resistance ones. Copper and Cobalt-zinc-cadmium resistance genes were the most widespread (detected in 56 and 45 MAGs respectively) (Fig. 4), as also recently highlighted in another study on Venice Lagoon sediments (Curran et al. 2022). The high abundance of toxic compounds resistance genes was consistent with the high concentration of such elements (e.g*.*, cadmium, copper, chromium, zinc) in the sediment of this area (Curran et al. 2022; Zonta et al. 2020). Beside the general reduction trend in the Lagoon due to sanitation measures, cadmium, copper, chromium, zinc concentrations are still above the international sediment quality guideline limits (Apitz et al. 2007; Zonta et al. 2020). In our dataset, all antibiotic resistant bacteria also carried information for toxic compound resistance (Fig. 4), a condition that may promote and therefore worsen the resistance genes spreading issue, heightening the urgency to enhance the monitoring system in the Venice Lagoon.

Genes related to the degradation pathways of various aromatic compounds were also found (aromatic amines, benzoate, biphenyls, phenols). Genes related to polycyclic aromatic hydrocarbons (PAHs) were the most abundant and evenly distributed within the MAGs: Salicylate and gentisate catabolism (present in 44 MAGs), followed by Gentisate degradation (43) and Quinate degradation (18) (Fig. 4). PAHs are important anthropogenic pollutants (Tobiszewski and Namieśnik 2012), and the presence and abundance of genes responsible for their degradation in the reconstructed genomes indicates a general influence of these persistent organic pollutants on lagoon microbial communities (Fig. 4, Supplementary Fig. S2). Indeed, PAHs (e.g*.,* naphthalene) that bind to particulate organic matter tend to deposit and accumulate in the sediment (Hussar et al. 2012), and their potential toxicity may pose a hazard to humans, animals, and to the environment. In the Venice Lagoon, surface sediments have been assessed for PAHs contamination, largely due to fossil fuel combustion and, to a lesser extent, petroleum spills (Cassin et al. 2018; Zonta et al. 2020). PAHs contamination was detected across the Lagoon at a wide range of concentrations, with higher levels near industrial areas, fishing farms, and in areas impacted by car and boat traffic and urban waste discharge (Cassin et al. 2018; Zonta et al. 2020).

### Microscale microbial ecological dynamics

Marine sediment is a highly structured environment, characterized by chemical and organic matter gradients (Zinger et al. 2011), in which Bacteria are competing within microscale niches for resources and fighting for living. The successful microbes thrive given the adaptive strategies present in their genomes. We operationally defined microscale-related genes in our dataset belonged to DNA metabolism (DNA uptake and competence), Membrane transport (ABC transporters, Cation transporters, Ton and Tol systems, Type II, Type IV), Mobilome (Pathogenicity islands, Phages and Prophages), and Regulation and Cell Signaling (Osmotic stress, Stringent Response, Toxin-antitoxin Systems) (Banchi et al. 2021) categories (Fig. 4). Within the above-mentioned categories, the most frequently found genes were belonged to DNA uptake and competence (present in 48 MAGs), Cation transporters (57), Phages, Prophages (23), and Stringent response (48) respectively. We did not identify a clear relationship between the microscale category genes and taxonomy. The emerging pattern suggested a ‘must-have’ toolkit composed by functions related to DNA uptake and competence, Cation transporters, Ton and Tol systems, ABC transporters, osmotic stress, and stringent response. This indicated that the microbes living in an ever-changing sediment habitat such as Venice Lagoon have developed successful adaptive strategies to efficiently uptake micronutrients such as cation important cofactors of enzymes (Waldron et al. 2009; Waldron and Robinson 2009) and/or to export toxic metals outside the cells (Hagström et al. 2021; Paulsen and Saier 1997). Given the high diversity of transporters, a wide range of molecules from amino acids, sugars, inorganic ions and informative molecules such as DNA (Davies et al. 2021; Finkel and Kolter 2001; Mell and Redfield 2014) are traded in the microbial sediment ecosystem. In Gram-negative, Tol and Ton systems have been thoroughly studied in the context of virulence and pathogenesis (Hirakawa et al. 2022). The Tol system, originally port of entry for toxins and bacteriophages in *Escherichia coli* (Szczepaniak et al. 2020) is important in stabilizing the outer membrane during cell division and its homeostasis. The Tol system is a major antibiotic efflux channel while the Ton system plays a role in transporting siderophores, vitamin B12, nickel complexes, and carbohydrates through the outer membrane (Noinaj et al. 2010). From the metabolic point of view, being able to counteract the osmotic changes by pumping solutes into the cells from the environment or producing small osmolytes is highly valuable in a lagoon system (Wood 2015 and reference therein). The presence of functions associated with the stringent response indicates that microbes regularly experience starvation due to lack of nutrients and in order to persist they need to re-wire their metabolism and slow down gene expression related to rRNA, tRNA and cell division that is not essential, thus allocating the limited energetic resources for amino acid biosynthetic and stress survival pathways (Irving et al. 2021; Milewska et al. 2020).

Important microscale-related genes, that presented narrower distributions within the MAGs, were Secretion System Type II and IV, toxin-antitoxin module, Pathogenicity islands, and Phages and Prophages (Fig. 4). Type II and Type IV are nanomachines that span through the inner membrane-peptidoglycan-outer membrane sandwich-like structure of the Gram-negative (Costa et al. 2015). These systems secrete a wide range of molecules from the cytoplasm to the exterior such as proteases, lipases, adhesion factors, exotoxins and DNA, Type II and IV specifically (Costa et al. 2015). Furthermore, Type IV is structurally a pilus that can extend and retract to allow a non-flagellar based motility, thus also favouring adhesion and biofilm formation (Ligthart et al. 2020). Mobile genetic elements are pervasive features of microbial life (Koonin et al. 2020), and they come in different shapes and are very heterogeneous. Phages, insertion sequences, transposons and plasmids are part of the mobilome. Over evolutionary time, gene swapping has contributed to evolution and high diversity of structure and functions at the microbial level (Koonin et al. 2020). To persist, the mobilome, being by definition not part of the regular cellular genes, needs to have some degree of selfishness such as the toxin-antitoxin system or the use of the lytic phage strategy (Van Melderen and Saavedra De Bast 2009). The picture that the MAGs describe is a dynamic genetic landscape that changes upon horizontal gene transfer, carrying diverse mobile genetic elements. Some of them, the phages, ultimately, also control the microbes at the population level to persist in the environment.

### Biosynthetic gene clusters

Marine microorganisms are constantly interacting and competing with other microbes for resources and nutrients. A key strategy to thrive within the highly competitive environment is the microbial production of bioactive compounds/secondary metabolites (Gozari et al. 2021; Patin et al. 2017). Within this category, there are compounds with pharmaceutical and biotechnological applications, antimicrobial potential, antibiotics, drugs, and siderophores. Such metabolites are commonly produced by pathways in which the genes involved are clustered locally on the chromosome: the Biosynthetic Gene Clusters (BGCs) (Blin et al. 2021). For this reason, we examined our dataset for the presence of BGCs, important for future bioprospecting research of unexplored ecological niches (Paoli et al. 2022).

**Fig. 5:**
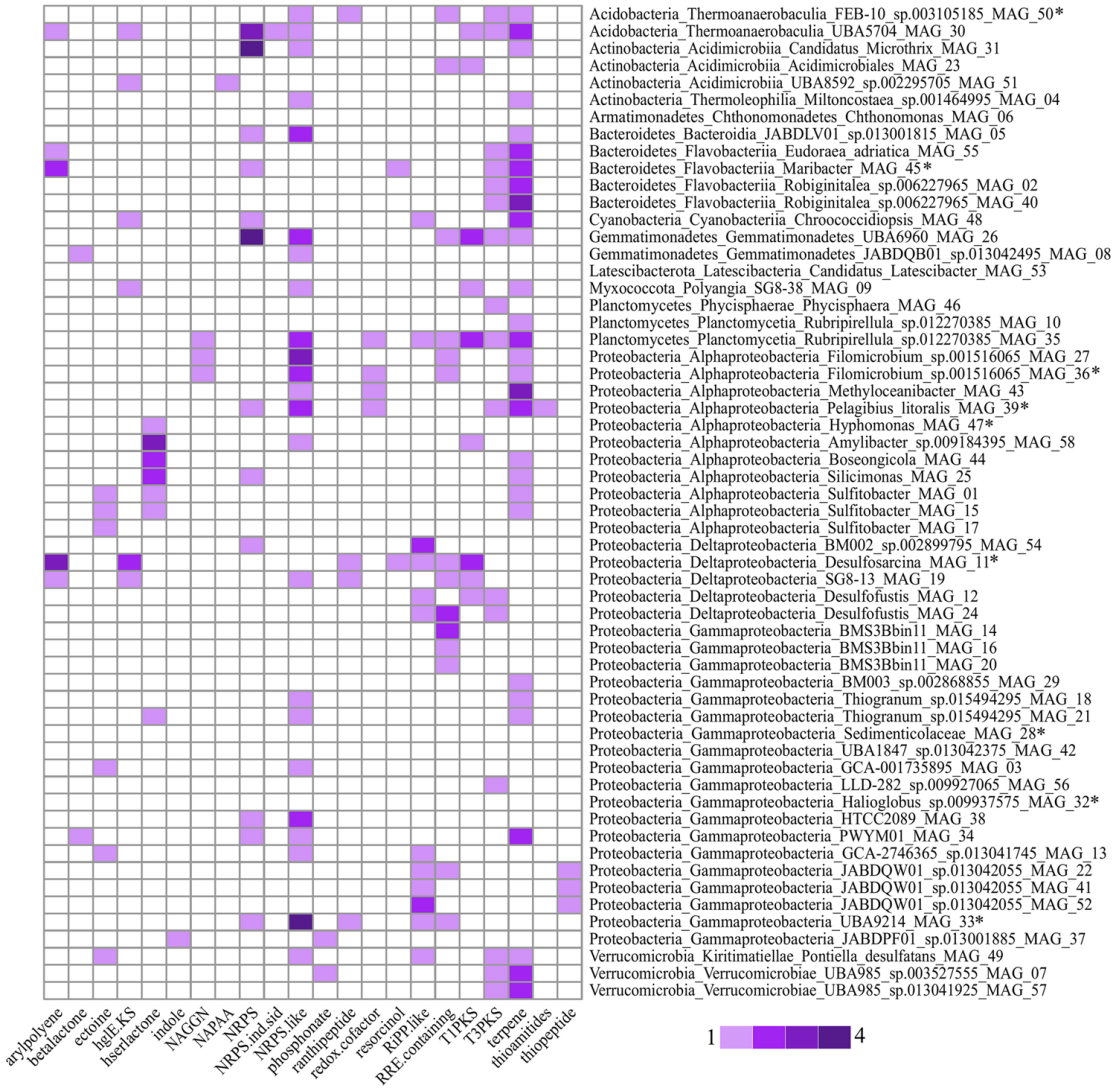
Heatmap of the biosynthetic gene clusters (BGCs) detected in the MAG dataset. Asterisks indicate high-quality MAGs. Betalactone: beta-lactone containing protease inhibitor; PKS: polyketide synthase; hglE-KS: heterocyst glycolipid synthase-like PKS; hserlactone: homoserine lactone cluster; NAGGN: N-acetylglutaminylglutamine amide; NAPAA: non-alpha poly-amino acids like e-Polylysin; NRPS: non-ribosomal peptide synthetase cluster; NRPS-ind-sid: NRPS-independent-siderophore; RiPP-like: ribosomally synthesised and post-translationally modified peptide product cluster; RRE: RiPP recognition element

Biosynthetic gene clusters analysis in the MAG dataset revealed that 53 out of 58 genomes contained at least one BGC belonging to 22 different classes (Fig. 5). Such array highlighted a wide range and a variable distribution pattern among genomes, including clusters related to (i) antimicrobial potential (e.g*.*, ribosomally synthesized and post-translationally modified peptides, RiPPs, including Thipeptides and Ranthipeptide, beta-lactone, non-alpha poly-amino acids like e-polylysin), (ii) pharmaceutical and biotechnological potential (e.g*.*, Thioamitide RiPPs, polyketide synthases Type I and Type III), (iii) Quorum Sensing (e.g*.,* Homoserine lactone), (iv) siderophore biosynthesis (e.g*.,* NRPS-independent-siderophore), (v) osmotic stress response (e.g*.,* ectoine).

The most widespread BGCs were terpenes (present in 29 MAGs) (Fig. 5). Their prevalence is a common feature in benthic communities (Bruce et al. 2022), and sediments are considered the main source of terpene producing microorganisms within the marine environment (Gozari et al. 2021). Terpenes are the largest group of natural products that play important roles in organism interactions, mechanisms defense, and physiological functions (*e.g.*, antioxidant properties, membrane stabilization) (De Carvalho and Fernandez 2010; Gershenzon and Dudareva 2007). Several terpenes isolated from sediment bacteria have been found to have cytotoxic and antimicrobial properties, such as the meroterpenoid ctinoranone (Nam et al. 2013) and the bromo-phenazinone meroterpenoids marinocyanins (Asolkar et al. 2017). Marine Actinobacteria are commonly found as the largest source of these natural products (Gozari et al. 2021; Manivasagan et al. 2014), whereas in our BGC dataset Proteobacteria scored as major producers (Fig. 5). The Venice Lagoon spectrum of BGCs showed a broad phylogenetic distribution. We believe that such microbial communities, with their high biosynthetic capacity, may be good candidates for exploring BGCs with more targeted approaches such as single-cell isolation and sequencing (Geers et al. 2021) and culture-based studies coupled with chemical identification by MALDI-based imaging mass spectrometry (Fang and Dorrestein 2014).

### MAGs overview

To depict the comprehensive picture of the MAG microbial metabolic processes and adaptations in the surface sediment of the Venice Lagoon, we have performed a cluster analysis based on presence/absence data of the functional annotations (Supplementary Fig. S3). The clustering highlighted the most prevalent and relevant features of the reconstructed genomes, represented by traits spanning throughout all investigated categories.

It has emerged that top “must have” strategies were glycolysis and fermentation, DNA competence, cation transporters, thus including Copper, Cobalt, Zinc, Mercury, and Cadmium followed by the stringent response and the osmotic shock regulation, the antibiotic resistance (i.e*.*, Beta-lactamase, Fluoroquinolones), and the ability to degrade xenobiotic compounds (e.g*.*, PAHs). Deltaproteobacteria and a group of Gammaproteobacteria (e.g., *Thiogranum* sp., *BMS3Bbin11* sp.) were characterized by dissimilatory sulfate reduction and thiosulphate oxidation potential. Furthermore, Deltaproteobacteria were described by many annotations within N cycle whereas the Gammaproteobacteria by CO_2_ transformation into biomass genes. Terpene and NRPS were enriched in Alphaproteobacteria, Bacteroidetes, and Verrucomicrobia.

Overall, the potential to live without oxygen emerged as one of the most important requirements to flourish in the recurrent conditions of oxygen depletion in these sediments. Mixotrophy was another widespread feature that allows microbes to flexibly use different energy sources. The complex sediment environment influenced the presence of niches at the microscale level, where microbes can live and thrive with tailored strategies, such as micronutrient transport, DNA uptake and detoxification potential. The microscale local environment is intrinsically linked to the landscape of secondary metabolites. Most MAGs encoded for a broad range of biosynthetic gene clusters that may be useful in interacting with other microbes for energy and resource management and highlighted a great biotechnological potential of the sediment communities. The Venice Lagoon presents high levels of contamination (Cassin et al. 2018; Zonta et al. 2020), which affects the diversity, function, and structure of microbial communities in the surface sediment (Banchi et al. 2021; Lyautey et al. 2021). Our results showed that all anthropogenic activities (*e.g.*, tourism, waste discharge, industry, farms, transports) have impacts on genetic resistance potential to antibiotics and heavy metals, as well as on the capacity to degrade aromatic compounds, especially PAHs.

## Conclusion and future perspectives

The analysis of the reconstructed MAGs revealed that the surface sediment bacteria of the Venice Lagoon can cope with environmental pressures and may enhance the ecosystem stability and resilience by integrating different strategies: (i) metabolic plasticity as different bacteria may use multiple energy metabolic strategies, (ii) functional redundancy with microbes capable of using the same metabolic pathways, and (iii) high biosynthetic potential with the presence of genes clusters encoding for a wide array of secondary metabolites production.

Our study represents the first effort to investigate genome-resolved metagenomics in the sediments of the Venice Lagoon. ‘Omics’ technologies enable the detailed study of prokaryotic communities in terms of biodiversity, dynamics, ecological role, and identification of important functional traits that can improve existing environmental monitoring and management tools (Bourlat et al. 2013; Pinhassi et al. 2022). In the perspective of increasing the number and the quality of MAGs, future experimental designs should expand both the sampling site coverage (e.g*.*, including the Venice city canals) and the vertical stratification (e.g*.,* deeper core layers), as well as the sequencing effort and approach (e.g*.*, the coupling with third generation sequencing platforms). This study represents an advance in the knowledge of surface sediment bacteria in the Venice Lagoon, allowing the definition of their ecological niches, their functional characterization, the distribution of ecosystem services, and the impact of human activities, completing and integrating community-level assessment using metagenomic data (Banchi et al. 2021; Pinhassi et al. 2022).

### Supplementary Information

Below is the link to the electronic supplementary material.

Supplementary file1 (PDF 1657 KB)

## Data Availability

The MAG dataset generated during the current study is available in the NCBI genome repository, with the accession number PRJNA924243 [http://www.ncbi.nlm.nih.gov/bioproject/924243].
