# Supplementary material for: Genome-resolved metagenomics of Venice Lagoon surface sediment bacteria reveals high biosynthetic potential and metabolic plasticity as successful strategies in an impacted environment
Source: Mar Life Sci Technol. 2023 Nov 3;6(1):126–42. doi: 10.1007/s42995-023-00192-z (PMC10902248; doi:10.1007/s42995-023-00192-z)
Supplement: Supplementary file 1 — Supplementary file1 (PDF 1657 KB) [file 42995_2023_192_MOESM1_ESM.pdf]

Supplementary information

Genome-resolved metagenomics of Venice Lagoon surface sediment bacteria reveals high biosynthetic potential and metabolic plasticity as successful strategies in an impacted environment

Elisa Banchi*[1], Erwan Corre[2], Paola Del Negro[1], Mauro Celussi*[1], Francesca Malfatti*[1,3]

[1]National Institute of Oceanography and Applied Geophysics – OGS, Trieste, Italy
[2]Roscoff Marine Station (SBR), Roscoff, France
[3]Department of Life Sciences, University of Trieste, Trieste, Italy

*Correspondence:
Elisa Banchi ebanchi@ogs.it
Mauro Celussi mcelussi@ogs.it
Francesca Malfatti fmalfatti@units.it

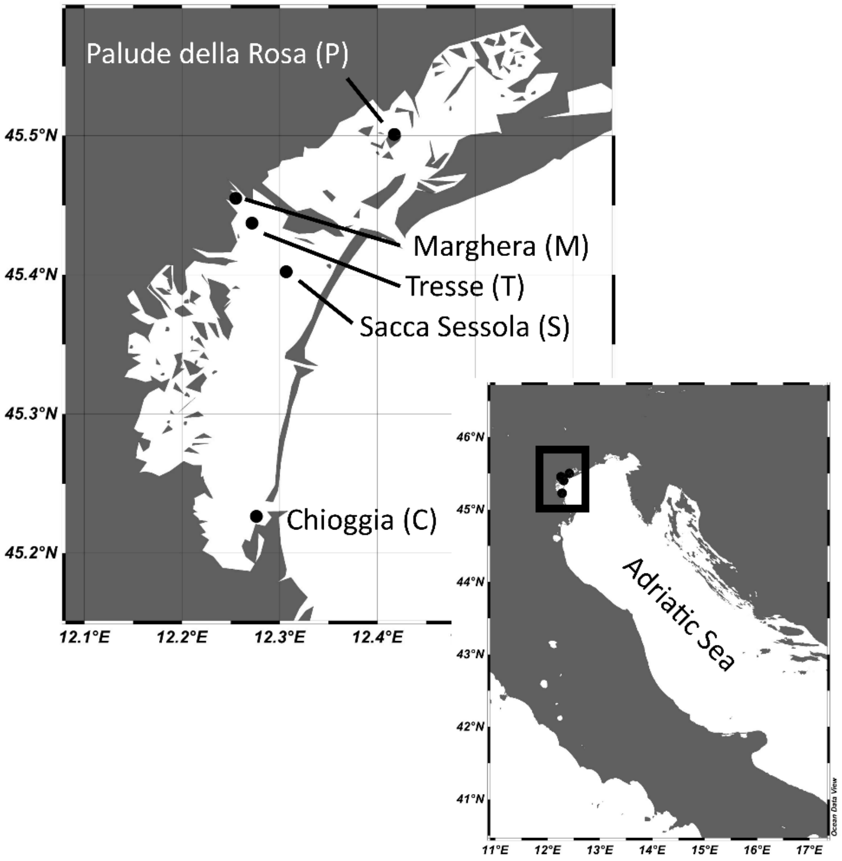

Supplementary Fig. S1 Sampling sites in the Venice Lagoon. Maps were generated using Ocean Data View software (Schlitzer 2018)

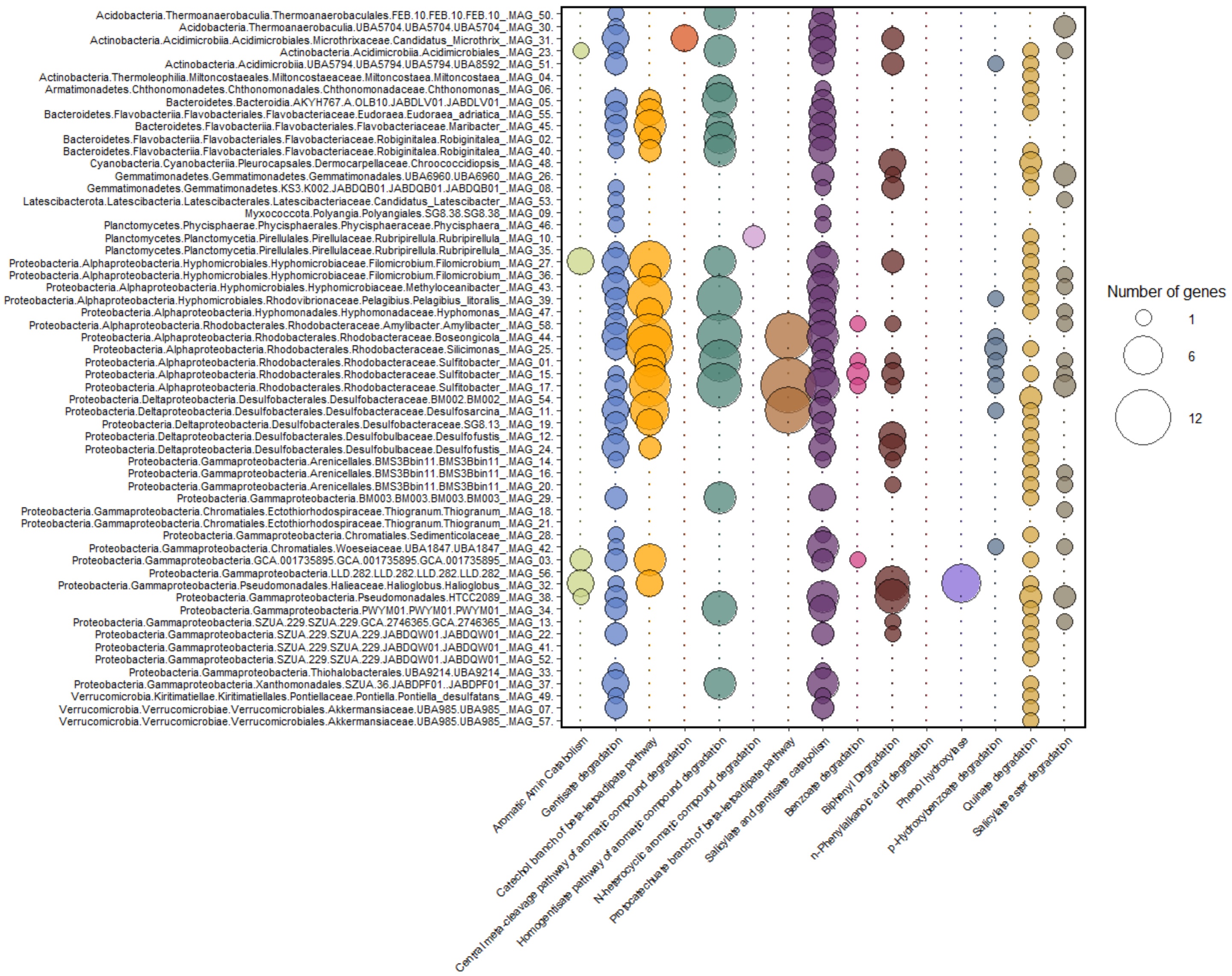

Supplementary Fig. S2 Presence and abundance of genes coding for aromatic compounds

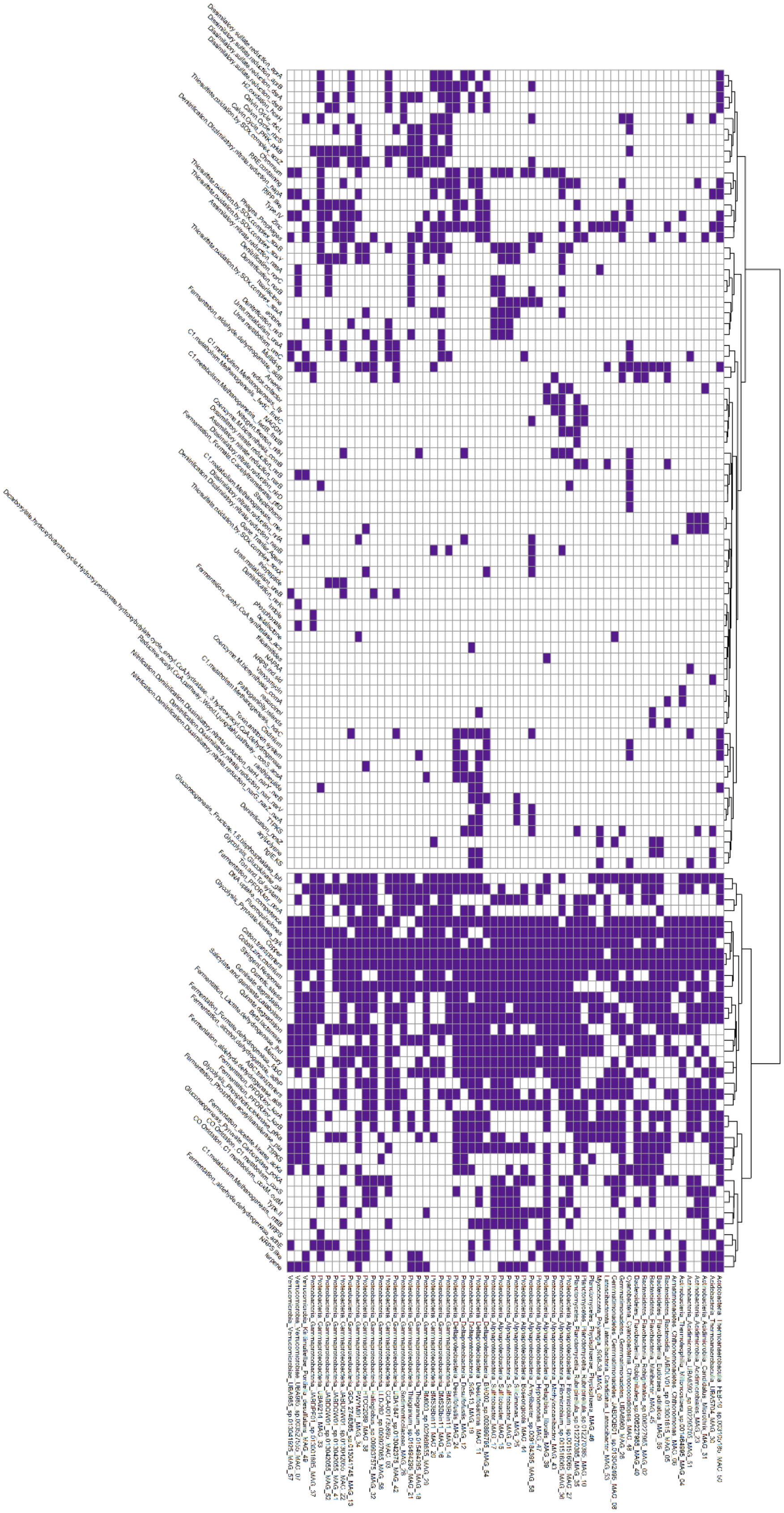

Supplementary Fig. S3 Heatmap showing the presence/absence of the investigated genes and gene categories.

Supplementary Table S1 Sampling stations and coordinates in the Venice Lagoon. C: Chioggia; M: Marghera; P: Palude della Rosa; S: Sacca Sessola; T: Tresse

Station	Coordinates (N, E)	Winter 2019	Spring 2019	Summer 2019	Autumn 2019
C	45°13'59.8", 12°16'56.2"	feb-13	mag-15	aug-22	11-apr
M	45°27'30.3", 12°15'32.5"	feb-13	mag-15	aug-22	11-apr
P	45°30'06.1", 12°25'03.0"	feb-13	mag-15	aug-22	11-apr
S	45°24'13.608", 12°18'38.844"	mar-20	mag-24	lug-23	24-oct
T	45°26'23.44", 12°16'29.207"	mar-20	mag-24	lug-23	24-oct

Supplementary Table S2 Bins and MAGs reconstructed from Venice Lagoon sediment samples. C: Chioggia; M: Marghera; P: Palude della Rosa; S: Sacca Sessola; T: Tresse

	Assembly		*Binning*					*MAGs quality*	
**Sample**	**MegaHIT tot**	**>1000 bp**	**MetaBAT2**	**Maxbin2**	**CONCOCT**	**DAS_Tool**	**CheckM**	**High**	**Medium**
C	8071416	541844	49	205	117	7	2	0	1
C_Aut	5460086	278545	26	102	91	2	1	0	0
C_Spr	6605893	406551	32	153	106	15	7	0	6
C_Sum	5746752	413773	45	155	131	12	5	0	4
C_Win	5701087	364366	29	149	88	4	2	0	1
M	6105291	457180	62	186	154	10	5	0	4
M_Aut	4889959	350124	47	139	145	12	5	0	5
M_Spr	5190510	391885	49	155	123	8	3	1	1
M_Sum	4958138	377081	54	154	141	10	2	0	1
M_Win	5033271	380604	62	157	123	13	4	0	3
P	6474034	541008	88	240	152	16	8	0	7
P_Aut	4983406	394990	62	174	130	14	4	0	3
P_Spr	5103404	475895	78	213	136	24	15	0	14
P_Sum	5059836	416620	67	185	129	20	7	0	6
P_Win	1501500	95625	14	40	46	4	0	0	0
S	9714147	755696	122	308	186	37	20	6	13
S_Aut	5128743	378248	59	145	135	18	6	1	5
S_Spr	5211901	385002	60	165	124	14	7	1	5
S_Sum	8518367	567858	86	226	145	19	8	0	7
S_Win	5137825	406968	70	188	128	20	10	3	6
T	7050400	546125	68	204	153	13	9	1	7
T_Aut	3986644	237776	28	84	99	7	1	0	1
T_Spr	5686041	443748	61	181	132	14	6	0	5
T_Sum	5360694	346531	37	135	119	8	3	0	2
T_Win	4902770	412010	68	160	132	17	6	0	6

Supplementary Table S3 Metrics of the MAGs reconstructed from Venice Lagoon sediment samples. C: Chioggia; M: Marghera; P: Palude della Rosa; S: Sacca Sessola; T: Tresse

**MAG**	**Size**	**GC Content**	**N50**	**L50**	**Number of Contigs (with**	**Number of Subsystems**	**Number of Coding**	**Completeness**	**Contamination**	**Strain heterogeneity**	**Bin quality**	**Binning program**
C_concoct.35	2.042.011	48.5	2678	255	827	186	2549	63.59	3.72	15.6	Medium	Concoct
C_Spr_bin.12	2.163.243	60.7	5438	132	431	179	2472	50.44	1.72	0	Medium	Metabat
C_Spr_bin.4	1.582.193	56	6020	85	283	176	1833	68.75	1.52	28.57	Medium	Metabat
C_Spr_bin.7	2.370.794	47.4	5579	146	454	178	2854	68.49	10	25	Medium	Metabat
C_Spr_concoct.23	2.282.350	58.7	2824	283	891	221	3063	68.28	4.67	60	Medium	Concoct
C_Spr_concoct.41	2.179.716	48.2	2688	263	885	203	2756	67.83	2.64	66.67	Medium	Concoct
C_Spr_concoct.90	3.066.138	53	7142	124	546	235	3415	82.55	4.69	29.17	Medium	Concoct
C_Sum_concoct.103	1.994.402	72.1	7572	87	306	170	2260	76.94	5.2	23.08	Medium	Concoct
C_Sum_concoct.15	2.523.999	45	3870	214	789	193	2810	87.13	3.64	41.67	Medium	Concoct
C_Sum_concoct.30	1.248.605	55.6	2346	190	590	103	1639	56.92	5.23	22.22	Medium	Concoct
C_Sum_concoct.69	3.174.934	53	5.225	189	710	187	3356	78.99	2.41	20	Medium	Concoct
C_Win_bin.5	2.781.272	51.6	7839	108	408	197	2952	84.47	4.95	20	Medium	Metabat
M_Aut_bin.39	1.763.941	59	5074	116	368	168	2102	64.67	1.56	12.5	Medium	Metabat
M_Aut_concoct.2	2.636.098	64.3	3384	267	825	160	3110	55.38	2.35	0	Medium	Concoct

M_Aut_concoct.75	2.688.825	62.4	3290	284	893	153	3089	54.01	0.85	0	Medium	Concoct
M_bin.39	2.100.329	58.9	7107	89	342	208	2418	76.49	2.63	30	Medium	Metabat
M_concoct.127	5.020.657	58.1	2279	771	2356	181	6280	56.46	5.29	0	Medium	Concoct
M_concoct.139	2.918.641	62.7	2511	416	1224	174	4088	57.99	4.27	11.11	Medium	Concoct
M_concoct.35	2.796.047	62.2	3332	281	891	158	3208	57.53	2.78	40	Medium	Concoct
M_Spr_bin.12	3.544.683	60.6	44248	22	168	268	3548	93.43	6.28	0	Medium	Metabat
M_Spr_bin.15	6.676.887	48	12674	158	689	274	7621	91.72	3.25	14.29	High	Metabat
M_Spr_bin.34	2.766.880	51.7	8431	101	379	197	2942	74.26	0	0	Medium	Metabat
M_Sum_bin.34	2.477.696	48.1	7720	102	375	209	2830	78.73	9.42	24.44	Medium	Metabat
M_Win_bin.13	2.001.224	55.9	8356	73	270	199	2217	82.15	4.88	20	Medium	Metabat
M_Win_bin.27	2.146.801	58.9	8838	70	300	217	2467	83.6	3.68	18.18	Medium	Metabat
M_Win_concoct.7	2.400.848	58.6	2516	324	1044	242	3239	69.74	3.58	66.67	Medium	Concoct
M_Win_concoct.71	3.190.183	62.2	3942	265	901	193	3535	78.43	0.9	0	Medium	Concoct
P_Aut_bin.28	3.747.121	54.4	7081	176	621	213	4249	81.72	1.17	23.43	Medium	Metabat
P_Aut_bin.39	2.558.259	55.1	7310	106	390	196	2918	70.06	4.51	24.32	Medium	Metabat
P_Aut_bin.56	3.834.933	60.3	47134	24	223	273	3925	93.35	6.44	5.88	Medium	Metabat
P_Aut_bin.8	3.747.121	54.4	7081	176	621	213	4249	77.12	3.56	12.5	Medium	Metabat
P_bin.23	1.903.078	38.7	4600	145	430	155	2138	52.58	0.89	33.33	Medium	Metabat
P_bin.57	2.780.230	47	49748	19	139	236	2943	95.09	6.44	21.43	Medium	Metabat
P_bin.7	3.987.431	54.7	9128	130	517	237	4356	76.76	1.31	20	Medium	Metabat
P_bin.71	3.529.995	60.5	46970	23	149	263	3537	93.32	5.49	7.69	Medium	Metabat
P_bin.85	2.962.348	54.9	8017	118	433	225	3367	81.21	3.97	15	Medium	Metabat
P_concoct.112	3.678.150	68.2	4345	276	951	190	3995	62.42	5.49	20	Medium	Concoct
P_concoct.94	2.354.269	58.7	2745	286	943	247	3154	69.04	2.17	61.54	Medium	Concoct
P_Spr_bin.18	2.138.065	56	6071	116	378	155	2537	53.69	1.81	18.18	Medium	Metabat
P_Spr_bin.22	4.123.915	58.7	10676	109	511	254	4749	91.85	7.89	18.92	Medium	Metabat
P_Spr_bin.24	2.780.048	46.8	48274	18	123	237	2929	96.34	9.18	12	Medium	Metabat
P_Spr_bin.27	3.559.848	55.3	11.150	96	411	230	3752	73.51	2.28	40	Medium	Metabat
P_Spr_bin.28	2.329.436	47.4	16.396	46	203	212	2500	88.11	1.86	75	Medium	Metabat
P_Spr_bin.38	2.256.321	38.5	4709	169	497	191	2538	65.13	1.13	28.57	Medium	Metabat
P_Spr_bin.56	2.345.323	49.5	12107	46	235	169	2607	64.94	1.69	9.09	Medium	Metabat
P_Spr_bin.6	3.088.988	54.8	7071	132	487	233	3529	85.4	7.48	6.25	Medium	Metabat
P_Spr_bin.62	3.725.458	60.5	42802	26	187	269	3748	94.38	5.62	0	Medium	Metabat
P_Spr_bin.68	3.079.144	55	8389	116	428	217	3471	80.23	2.46	6.25	Medium	Metabat
P_Spr_bin.74	3.411.645	69.7	8586	128	503	203	3722	73.12	4.78	0	Medium	Metabat
P_Spr_bin.75	3.636.440	54.5	9536	117	479	246	3905	81.24	4.89	16.67	Medium	Metabat
P_Spr_bin.78	3.252.905	60.9	4995	223	684	232	3809	70.2	6.96	25.71	Medium	Metabat
P_Spr_concoct.24	4.145.213	68.2	5708	231	832	201	4223	82.44	6.59	23.78	Medium	Concoct
P_Sum_bin.26	3.737.593	55	6795	177	616	221	4171	72.31	4.22	25	Medium	Metabat
P_Sum_bin.4	3.716.776	60.5	42848	25	188	268	3747	93.11	6.08	13.33	Medium	Metabat
P_Sum_bin.47	2.267.142	49.5	13575	50	205	209	2447	81.89	2.79	38.46	Medium	Metabat
P_Sum_bin.5	3.288.251	64	5456	196	644	222	3768	63.49	3.32	14.29	Medium	Metabat
P_Sum_bin.52	2.973.342	55	7224	130	457	223	3423	76.9	4.52	23.81	Medium	Metabat
P_Sum_bin.61	1.355.703	58	6403	68	226	160	1560	57.84	0.8	33.33	Medium	Metabat
S_Aut_bin.1	3.509.078	60.6	64203	19	140	260	3498	94.05	4.08	4.55	High	Metabat
S_Aut_bin.18	3.648.874	56.1	8830	127	516	253	4181	87.73	4.03	3.45	Medium	Metabat
S_Aut_bin.37	4.253.777	58.6	17043	69	392	266	4724	94.24	5.76	13.79	Medium	Metabat
S_Aut_bin.51	3.434.496	63.6	20181	51	244	265	3497	91.13	1.74	25	High	Metabat
S_Aut_bin.7	1.603.588	49.5	11329	32	160	148	1733	60.03	0.41	0	Medium	Metabat
S_bin.101	3.126.677	62.4	5238	200	638	161	3253	71.89	2.61	0	Medium	Metabat
S_bin.103	2.031.858	48.1	6701	99	325	189	2306	65.57	2.53	33.33	Medium	Metabat
S_bin.112	2.855.127	57.5	10803	85	331	242	3253	85.07	2.91	57.14	Medium	Metabat
S_bin.12	3.169.618	67.2	4757	214	683	180	3551	54.53	7.76	31.25	Medium	Metabat
S_bin.19	3.689.536	58.4	24629	44	228	280	3554	97.2	3.03	11.11	High	Metabat
S_bin.34	3.838.475	55.9	15557	74	359	258	4173	91.37	3.58	0	High	Metabat
S_bin.40	3.750.835	65.4	27979	37	208	258	3674	92.41	6.04	14.29	Medium	Metabat
S_bin.65	6.038.647	58.2	6278	312	1073	187	6065	76.83	2.35	50	Medium	Metabat
S_bin.66	3.695.323	60.5	41880	25	161	274	3706	94.58	4.11	0	High	Metabat
S_bin.75	4.499.258	58.4	19594	62	379	271	5061	96.43	4.09	29.41	High	Metabat
S_bin.77	2.770.194	57.6	8772	92	375	215	2860	78.76	5.33	28.57	Medium	Metabat
S_bin.78	3.790.336	40	8476	133	520	227	4041	80.3	1.65	16.67	Medium	Metabat
S_bin.9	3.826.094	62.3	6265	184	677	216	4145	71.75	6.6	12.9	Medium	Metabat
S_bin.93	3.626.067	63.6	46856	25	141	277	3603	98.62	2.2	0	High	Metabat
S_concoct.155	2.598.018	57.2	2236	373	1257	154	3247	67.13	8.71	12.5	Medium	Concoct
S_concoct.158	1.967.358	48.3	2684	254	801	190	2509	66.67	6.65	29.73	Medium	Concoct
S_concoct.171	2.652.790	60.3	3726	234	826	160	3001	70.18	2.08	14.29	Medium	Concoct
S_concoct.178	2.778.667	59.3	5357	163	700	184	3105	78.1	5.51	8.33	Medium	Concoct
S_concoct.5	2.145.918	51.7	4715	149	562	231	2589	90.52	4.85	51.72	High	Concoct
S_Spr_bin.21	3.663.372	60.6	48713	25	174	264	3649	93.35	4.43	9.09	High	Metabat
S_Spr_bin.27	2.181.597	47.5	8278	82	297	190	2438	75.71	2.44	16.67	Medium	Metabat
S_Spr_bin.46	3.008.159	56.4	6453	149	509	225	3436	80.54	3.58	13.33	Medium	Metabat
S_Spr_bin.59	2.623.057	49.6	16008	44	218	198	2739	80.48	2.84	6.67	Medium	Metabat
S_Spr_bin.60	2.468.720	63.9	4921	172	530	218	2823	71.99	9.65	3.23	Medium	Metabat
S_Spr_concoct.116	5.827.868	57.9	3293	605	1883	186	6710	63.62	6.47	14.29	Medium	Concoct
S_Sum_bin.1	4.329.003	58.7	15012	76	446	274	4883	93.63	7.46	14.29	Medium	Metabat

S_Sum_bin.38	3.407.040	56.4	8260	127	483	223	3828	83.86	3.68	8.7	Medium	Metabat
S_Sum_bin.48	3.467.848	59.2	10598	95	450	221	3623	60.53	9.6	18.18	Medium	Metabat
S_Sum_bin.59	3.550.579	60.6	46101	22	161	265	3555	94.21	6.94	9.38	Medium	Metabat
S_Sum_bin.81	2.371.107	58.8	17539	38	206	218	2589	89.53	1.19	42.86	Medium	Metabat
S_Sum_concoct.101	2.531.474	57.3	2162	387	1269	158	3219	67.13	5.24	0	Medium	Concoct
S_Sum_concoct.74	6.849.056	58.1	4088	550	1840	222	7551	74.28	8.77	21.66	Medium	Concoct
S_Win_bin.3	2.916.120	57.5	12967	71	294	251	3278	90.51	2.3	69.23	Medium	Metabat
S_Win_bin.30	6.135.162	48.1	7200	250	963	274	7297	77.92	3.23	20	Medium	Metabat
S_Win_bin.45	4.230.583	40	17024	73	353	249	4281	94.46	1.77	7.69	High	Metabat
S_Win_bin.50	3.519.308	60.6	39030	24	151	262	3515	93.62	4.25	0	High	Metabat
S_Win_bin.62	1.979.236	58.7	16323	37	184	196	2190	80.32	1.65	14.29	Medium	Metabat
S_Win_bin.63	2.330.465	60.2	6010	127	419	148	2392	71.53	0	0	Medium	Metabat
S_Win_concoct.43	2.159.042	51.6	5432	124	523	236	2565	92.48	3.36	64.71	High	Concoct
S_Win_concoct.75	4.541.210	38.4	2995	506	1607	232	5702	70.89	7.57	19.05	Medium	Concoct
S_Win_concoct.80	2.867.194	59.5	8139	106	491	200	2998	85.14	4.77	36.36	Medium	Concoct
T_Aut_bin.9	2.429.947	49.6	13830	50	258	210	2639	81.89	6.56	34.29	Medium	Metabat
T_bin.11	5.074.682	63.3	24499	56	336	229	4673	96.15	5.13	0	High	Metabat
T_bin.21	3.360.193	69.5	6129	175	617	209	3740	82.24	7.26	7.69	Medium	Metabat
T_bin.27	1.847.532	55.7	7849	68	266	149	1877	73.81	3.91	13.33	Medium	Metabat
T_bin.36	2.531.447	57.4	6039	130	451	222	3037	70.29	2.07	38.46	Medium	Metabat
T_bin.40	2.163.969	58.9	10026	65	290	214	2428	84.01	1.34	28.57	Medium	Metabat
T_bin.52	3.586.958	62.6	16435	62	305	237	3874	87.67	5.13	0	Medium	Metabat
T_bin.56	1.883.436	53.3	6128	103	347	153	2191	60.77	3.93	31.58	Medium	Metabat
T_concoct.77	2.063.071	57.3	2264	302	972	134	2579	58.09	1.65	50	Medium	Concoct
T_Spr_bin.1	3.523.351	62.6	11355	100	415	239	3920	92.02	5.13	0	Medium	Metabat
T_Spr_bin.2	2.755.898	46.6	43559	21	151	226	2963	92.11	7.32	0	Medium	Metabat
T_Spr_bin.33	2.565.146	57.4	5461	151	491	229	3099	75.24	5.1	70	Medium	Metabat
T_Spr_bin.42	2.936.854	60.8	9882	100	398	225	3156	77.18	5.75	3.7	Medium	Metabat
T_Spr_bin.52	2.215.134	58.8	6894	93	356	206	2576	79.02	2.45	30.77	Medium	Metabat
T_Sum_bin.3	1.525.079	50.3	15394	26	151	147	1611	59.55	2.51	26.09	Medium	Metabat
T_Sum_concoct.63	3.924.940	50.4	3139	447	1301	191	5461	53.12	2.4	25	Medium	Concoct
T_Win_bin.25	1.954.044	38.6	5011	137	418	151	2126	58.01	1.49	14.29	Medium	Metabat
T_Win_bin.3	5.347.394	63.4	20291	70	431	232	4960	95.98	6.69	72.22	Medium	Metabat
T_Win_bin.30	3.066.920	57.5	12700	77	328	262	3465	89.4	3.37	52.94	Medium	Metabat
T_Win_concoct.100	943.598	24.9	3181	97	346	115	1135	64.52	1.94	0	Medium	Concoct
T_Win_concoct.29	3.696.916	57.7	7713	160	605	199	3566	89.56	1.08	72.42	Medium	Concoct
T_Win_concoct.89	2.206.040	46.6	2895	258	865	232	2901	71.72	1.7	10	Medium	Concoct

Supplementary Table S4 Metrics and taxonomy of the non-redundant MAG dataset. C: Chioggia; M: Marghera; P: Palude della Rosa; S: Sacca Sessola; T: Tresse

MAG	MAG_old name (Table S3)	Size	Completeness	Contamination	Strain heterogeneity	SEED	KEGG	Taxonomy
MAG_Ve_Sed_01	C_Spr_concoct.23	2282350	68.28	4.67	60	658	1303	Proteobacteria;Alphaproteobacteria;Rhodobacterales;Rhodobacteraceae; Sulfitobacter_(MAG_01)
MAG_Ve_Sed_02	C_Spr_concoct.41	2179716	67.83	2.64	66.67	540	955	Bacteroidetes;Flavobacteriia;Flavobacteriales; Flavobacteriaceae;Robiginitalea;Robiginitalea_sp.006227965_(MAG_02)
MAG_Ve_Sed_03	C_Spr_concoct.90	3066138	82.55	4.69	29.17	703	1569	Proteobacteria;Gammaproteobacteria;GCA-001735895; GCA-001735895;GCA-001735895_(MAG_03)
MAG_Ve_Sed_04	C_Sum_concoct.103	1994402	76.94	5.2	23.08	449	937	Actinobacteria;Thermoleophilia;Miltoncostaeales;Miltoncostaeaceae; Miltoncostaea;Miltoncostaea_sp.001464995_(MAG_04)
MAG_Ve_Sed_05	C_Sum_concoct.15	2523999	87.13	3.64	41.67	509	962	Bacteroidetes;Bacteroidia;AKYH767-A;OLB10;JABDLV01; JABDLV01_sp.013001815_(MAG_05)
MAG_Ve_Sed_06	C_Sum_concoct.30	1248605	56.92	5.23	22.22	238	608	Armatimonadetes;Chthonomonadetes;Chthonomonadales;Chthonomonadaceae; Chthonomonas_(MAG_06)
MAG_Ve_Sed_07	C_Sum_concoct.69	3174934	78.99	2.41	20	565	1035	Verrucomicrobia;Verrucomicrobiae;Verrucomicrobiales;Akkermansiaceae ;UBA985;UBA985_sp.003527555_(MAG_07)
MAG_Ve_Sed_08	C_Win_bin.5	2781272	84.47	4.95	20	588	1038	Gemmatimonadetes;Gemmatimonadetes;KS3-K002;JABDQB01; JABDQB01;JABDQB01_sp.013042495_(MAG_08)
MAG_Ve_Sed_09	M_Aut_concoct.2	2636098	55.38	2.35	0	407	1028	Myxococcota;Polyangia;Polyangiales;SG8-38; SG8-38_(MAG_09)
MAG_Ve_Sed_10	M_concoct.127	5020657	56.46	5.29	0	505	1196	Planctomycetes;Planctomycetia;Pirellulales;Pirellulaceae; Rubripirellula;Rubripirellula_sp.012270385_(MAG_10)
MAG_Ve_Sed_11	M_Spr_bin.15	6676887	91.72	3.25	14.29	980	2257	Proteobacteria;Deltaproteobacteria;Desulfobacterales; Desulfobacteraceae;Desulfosarcina_(MAG_11)

MAG_Ve_Sed_12	M_Spr_bin.34	2477696	78.73	9.42	0	538	1295	Proteobacteria;Deltaproteobacteria;Desulfobacterales; Desulfobulbaceae;Desulfofustis_(MAG_12)
MAG_Ve_Sed_13	M_Sum_bin.34	2001224	82.15	4.88	24.44	593	1263	Proteobacteria;Gammaproteobacteria;SZUA-229;SZUA-229; GCA-2746365;GCA-2746365_sp.013041745_(MAG_13)
MAG_Ve_Sed_14	M_Win_bin.13	2400848	69.74	3.58	20	605	1152	Proteobacteria;Gammaproteobacteria;Arenicellales;BMS3Bbin11; BMS3Bbin11_(MAG_14)
MAG_Ve_Sed_15	M_Win_concoct.7	2780230	95.09	6.44	66.67	742	1389	Proteobacteria;Alphaproteobacteria;Rhodobacterales;Rhodobacteraceae; Sulfitobacter_(MAG_15)
MAG_Ve_Sed_16	P_bin.57	2354269	69.04	2.17	21.43	824	1389	Proteobacteria;Gammaproteobacteria;Arenicellales;BMS3Bbin11; BMS3Bbin11_(MAG_16)
MAG_Ve_Sed_17	P_concoct.94	2138065	53.69	1.81	61.54	770	1398	Proteobacteria;Alphaproteobacteria;Rhodobacterales;Rhodobacteraceae; Sulfitobacter_(MAG_17)
MAG_Ve_Sed_18	P_Spr_bin.18	3559848	73.51	2.28	18.18	386	969	Proteobacteria;Gammaproteobacteria;Chromatiales;Ectothiorhodospiraceae; Thiogranum;Thiogranum_sp.015494295_(MAG_18)
MAG_Ve_Sed_19	P_Spr_bin.27	2329436	88.11	1.86	40	690	1438	Proteobacteria;Deltaproteobacteria;Desulfobacterales;Desulfobacteraceae; SG8-13_(MAG_19)
MAG_Ve_Sed_20	P_Spr_bin.28	3088988	85.4	7.48	75	674	1267	Proteobacteria;Gammaproteobacteria;Arenicellales;BMS3Bbin11; BMS3Bbin11_(MAG_20)
MAG_Ve_Sed_21	P_Spr_bin.6	3079144	80.23	2.46	6.25	766	1513	Proteobacteria;Gammaproteobacteria;Chromatiales;Ectothiorhodospiraceae; Thiogranum;Thiogranum_sp.015494295_(MAG_21)
MAG_Ve_Sed_22	P_Spr_bin.68	3411645	73.12	4.78	6.25	621	1603	Proteobacteria;Gammaproteobacteria;SZUA-229;SZUA-229;JABDQW01; JABDQW01_sp.013042055_(MAG_22)
MAG_Ve_Sed_23	P_Spr_bin.74	3636440	81.24	4.89	0	625	1342	Actinobacteria;Acidimicrobiia;Acidimicrobiales_(MAG_23)
MAG_Ve_Sed_24	P_Spr_bin.75	3252905	70.2	6.96	16.67	828	1607	Proteobacteria;Deltaproteobacteria;Desulfobacterales;Desulfobulbaceae; Desulfofustis_(MAG_24)
MAG_Ve_Sed_25	P_Spr_bin.78	4145213	82.44	6.59	25.71	742	1274	Proteobacteria;Alphaproteobacteria;Rhodobacterales;Rhodobacteraceae; Silicimonas_(MAG_25)
MAG_Ve_Sed_26	P_Spr_concoct.24	3288251	63.49	3.32	23.78	529	1313	Gemmatimonadetes;Gemmatimonadetes;Gemmatimonadales;UBA6960; UBA6960_(MAG_26)
MAG_Ve_Sed_27	P_Sum_bin.5	1355703	57.84	0.8	14.29	687	1436	Proteobacteria;Alphaproteobacteria;Hyphomicrobiales;Hyphomicrobiaceae; Filomicrobium;Filomicrobium_sp.001516065_(MAG_27)
MAG_Ve_Sed_28	P_Sum_bin.61	3509078	94.05	4.08	33.33	431	887	Proteobacteria;Gammaproteobacteria;Chromatiales;Sedimenticolaceae_(MAG_28)
MAG_Ve_Sed_29	S_Aut_bin.1	2477696	78.73	9.42	4.55	943	1657	Proteobacteria;Gammaproteobacteria;BM003;BM003;BM003; BM003_sp.002868855_(MAG_29)
MAG_Ve_Sed_30	S_bin.101	3126677	71.89	2.61	0	425	1129	Acidobacteria;Thermoanaerobaculia;UBA5704;UBA5704;UBA5704_(MAG_30)
MAG_Ve_Sed_31	S_bin.12	3169618	54.53	7.76	31.25	521	1207	Actinobacteria;Acidimicrobiia;Acidimicrobiales;Microthrixaceae; Candidatus_Microthrix_(MAG_31)
MAG_Ve_Sed_32	S_bin.19	3689536	97.2	3.03	11.11	1007	1779	Proteobacteria;Gammaproteobacteria;Pseudomonadales;Halieaceae; Halioglobus;Halioglobus_sp.009937575_(MAG_32)
MAG_Ve_Sed_33	S_bin.34	3838475	91.37	3.58	0	901	1588	Proteobacteria;Gammaproteobacteria;Thiohalobacterales;UBA9214; UBA9214_(MAG_33)
MAG_Ve_Sed_34	S_bin.40	3750835	92.41	6.04	14.29	989	1689	Proteobacteria;Gammaproteobacteria;PWYM01;PWYM01;PWYM01_(MAG_34)
MAG_Ve_Sed_35	S_bin.65	6038647	76.83	2.35	50	512	1304	Planctomycetes;Planctomycetia;Pirellulales;Pirellulaceae;Rubripirellula; Rubripirellula_sp.012270385_(MAG_35)
MAG_Ve_Sed_36	S_bin.75	4499258	96.43	4.09	29.41	1025	959	Proteobacteria;Alphaproteobacteria;Hyphomicrobiales;Hyphomicrobiaceae; Filomicrobium;Filomicrobium_sp.001516065_(MAG_36)
MAG_Ve_Sed_37	S_bin.77	2770194	78.76	5.33	28.57	698	1367	Proteobacteria;Gammaproteobacteria;Xanthomonadales;SZUA-36; JABDPF01;;JABDPF01_sp.013001885_(MAG_37)
MAG_Ve_Sed_38	S_bin.9	3826094	71.75	6.6	12.9	618	1585	Proteobacteria;Gammaproteobacteria;Pseudomonadales;HTCC2089_(MAG_38)
MAG_Ve_Sed_39	S_bin.93	3626067	98.62	2.2	0	978	1782	Proteobacteria;Alphaproteobacteria;Hyphomicrobiales;Rhodovibrionaceae; Pelagibius;Pelagibius_litoralis_(MAG_39)
MAG_Ve_Sed_40	S_concoct.158	1967358	66.67	6.65	29.73	501	862	Bacteroidetes;Flavobacteriia;Flavobacteriales;Flavobacteriaceae; Robiginitalea;Robiginitalea_sp.006227965_(MAG_40)
MAG_Ve_Sed_41	S_Spr_bin.59	2623057	80.48	2.84	6.67	569	1293	Proteobacteria;Gammaproteobacteria;SZUA-229;SZUA-229;JABDQW01; JABDQW01_sp.013042055_(MAG_41)
MAG_Ve_Sed_42	S_Sum_bin.48	3467848	60.53	9.6	18.18	663	1468	Proteobacteria;Gammaproteobacteria;Chromatiales;Woeseiaceae;UBA1847; UBA1847_sp.013042375_(MAG_42)
MAG_Ve_Sed_43	S_Sum_bin.81	2371107	89.53	1.19	42.86	722	1194	Proteobacteria;Alphaproteobacteria;Hyphomicrobiales;Hyphomicrobiaceae; Methyloceanibacter_(MAG_43)
MAG_Ve_Sed_44	S_Win_bin.3	2916120	90.51	2.3	69.23	848	1570	Proteobacteria;Alphaproteobacteria;Rhodobacterales;Rhodobacteraceae; Boseongicola_(MAG_44)
MAG_Ve_Sed_45	S_Win_bin.45	4230583	94.46	1.77	7.69	839	1359	Bacteroidetes;Flavobacteriia;Flavobacteriales;Flavobacteriaceae; Maribacter_(MAG_45)
MAG_Ve_Sed_46	S_Win_bin.63	2330465	71.53	0	0	363	815	Planctomycetes;Phycisphaerae;Phycisphaerales;Phycisphaeraceae; Phycisphaera_(MAG_46)
MAG_Ve_Sed_47	S_Win_concoct.43	2159042	92.48	3.36	64.71	813	1146	Proteobacteria;Alphaproteobacteria;Hyphomonadales;Hyphomonadaceae; Hyphomonas_(MAG_47)
MAG_Ve_Sed_48	S_Win_concoct.75	4541210	70.89	7.57	19.05	729	1610	Cyanobacteria;Cyanobacteriia;Pleurocapsales;Dermocarpellaceae; Chroococcidiopsis_(MAG_48)
MAG_Ve_Sed_49	S_Win_concoct.80	2867194	85.14	4.77	36.36	607	1194	Verrucomicrobia;Kiritimatiellae;Kiritimatiellales;Pontiellaceae; Pontiella;Pontiella_desulfatans_(MAG_49)

MAG_Ve_Sed_50	T_bin.11	5074682	96.15	5.13	0	719	1336	Acidobacteria;Thermoanaerobaculia;Thermoanaerobaculales;FEB-10;FEB-10;FEB-10_sp.003105185_(MAG_50)
MAG_Ve_Sed_51	T_bin.52	3586958	87.67	5.13	0	723	1448	Actinobacteria;Acidimicrobiia;UBA5794;UBA5794;UBA5794;UBA8592_sp.002295705_(MAG_51)
MAG_Ve_Sed_52	T_bin.56	1883436	60.77	3.93	31.58	439	968	Proteobacteria;Gammaproteobacteria;SZUA-229;SZUA-229;JABDQW01;JABDQW01_sp.013042055_(MAG_52)
MAG_Ve_Sed_53	T_concoct.77	2063071	58.09	1.65	50	307	830	Latescibacterota;Latescibacteria;Latescibacterales;Latescibacteriaceae;Candidatus_Latescibacter_(MAG_53)
MAG_Ve_Sed_54	T_Sum_concoct.63	3924940	53.12	2.4	25	476	1246	Proteobacteria;Deltaproteobacteria;Desulfobacterales;Desulfobacteraceae;BM002;BM002_sp.002899795_(MAG_54)
MAG_Ve_Sed_55	T_Win_bin.25	1954044	58.01	1.49	14.29	363	813	Bacteroidetes;Flavobacteriia;Flavobacteriales;Flavobacteriaceae;Eudoraea;Eudoraea_adriatica_(MAG_55)
MAG_Ve_Sed_56	T_Win_concoct.100	943598	64.52	1.94	0	290	528	Proteobacteria;Gammaproteobacteria;LLD-282;LLD-282;LLD-282;LLD-282_sp.009927065_(MAG_56)
MAG_Ve_Sed_57	T_Win_concoct.29	3696916	89.56	1.08	72.42	607	1105	Verrucomicrobia;Verrucomicrobiae;Verrucomicrobiales;Akkermansiaceae;UBA985;UBA985_sp.013041925_(MAG_57)
MAG_Ve_Sed_58	T_Win_concoct.89	2206040	71.72	1.7	10	686	1274	Proteobacteria;Alphaproteobacteria;Rhodobacterales;Rhodobacteraceae;Amylibacter;Amylibacter_sp.009184395_(MAG_58)

Supplementary Table S5 Covered fraction of MAGs on the metagenomic assemblies

Genome	C	C_Aut	C_Spr	C_Sum	C_Win	M	M_Aut	M_Spr	M_Sum	M_Win	P	P_Aut	P_Spr	P_Sum	P_Win	S	S_Aut	S_Spr	S_Sum	S_Win	T	T_Aut	T_Spr	T_Sum	T_Win
MAG_Ve_Sed_01	0.99	0.97	0.99	0.99	0.99	0.93	0.97	0.99	0.99	0.99	0.99	0.99	0.99	0.99	0.99	0.99	0.98	0.99	0.99	0.99	0.93	0.99	0.99	0.99	0.99
MAG_Ve_Sed_02	0.99	0.96	0.99	0.99	0.99	0.95	0.98	0.99	0.99	0.99	0.98	0.96	0.99	0.99	1.00	0.99	0.96	0.99	1.00	1.00	0.95	0.99	0.99	0.99	0.99
MAG_Ve_Sed_03	0.99	0.96	1.00	0.99	0.99	0.94	0.99	0.99	0.99	0.99	0.99	0.98	0.99	1.00	0.97	0.99	0.97	0.99	0.99	1.00	0.94	1.00	1.00	0.99	0.99
MAG_Ve_Sed_04	0.99	0.95	0.99	1.00	1.00	0.96	0.99	1.00	0.99	1.00	0.99	0.98	0.99	1.00	1.00	0.99	0.99	0.99	0.99	0.99	0.97	0.99	1.00	0.99	0.99
MAG_Ve_Sed_05	0.99	0.98	0.99	1.00	0.99	0.97	0.96	0.99	0.99	0.99	0.99	0.98	0.99	0.99	0.99	0.99	0.96	0.99	0.99	0.99	0.96	0.99	0.99	1.00	1.00
MAG_Ve_Sed_06	0.99	0.97	0.99	0.99	0.99	0.93	0.97	0.99	0.99	0.99	1.00	0.98	0.99	0.99	1.00	0.99	0.97	0.99	0.99	0.99	0.96	0.99	0.99	0.99	0.99
MAG_Ve_Sed_07	0.99	0.96	0.99	1.00	0.99	0.95	0.97	0.99	0.99	0.99	0.99	0.97	0.99	1.00	1.00	0.99	0.97	0.99	1.00	1.00	0.95	0.99	0.99	0.99	0.99
MAG_Ve_Sed_08	0.99	0.97	0.99	0.99	1.00	0.90	0.95	1.00	0.99	0.99	0.99	0.98	0.99	1.00	0.99	0.99	0.99	0.99	0.99	0.99	0.94	1.00	1.00	0.98	0.99
MAG_Ve_Sed_09	0.99	0.96	0.99	0.99	0.99	0.94	0.99	1.00	1.00	0.99	0.99	0.97	1.00	0.99	0.99	0.99	0.98	0.99	0.99	0.99	0.96	0.99	0.99	0.99	0.99
MAG_Ve_Sed_10	0.99	0.96	0.99	0.99	0.99	0.96	0.97	0.99	0.99	0.99	0.99	0.97	0.99	0.99	1.00	0.99	0.97	0.99	0.99	0.99	0.95	0.99	0.99	0.98	0.99
MAG_Ve_Sed_11	0.98	0.97	0.99	0.99	0.98	0.95	0.96	1.00	0.99	0.99	0.99	0.97	0.99	0.99	0.98	1.00	0.98	0.99	0.99	0.99	0.94	0.99	0.99	0.99	0.99
MAG_Ve_Sed_12	0.99	0.95	0.99	0.99	0.98	0.94	0.97	1.00	0.99	0.99	1.00	0.99	0.99	0.99	1.00	0.99	0.99	0.99	0.99	0.99	0.96	1.00	0.99	0.99	0.99
MAG_Ve_Sed_13	0.99	0.97	0.99	0.99	1.00	0.95	0.99	0.99	1.00	1.00	1.00	0.99	0.99	0.99	1.00	1.00	0.97	0.99	0.99	0.98	0.96	0.99	1.00	0.99	0.98
MAG_Ve_Sed_14	0.99	0.97	0.99	0.99	0.99	0.93	0.98	0.99	1.00	1.00	0.99	0.96	0.99	0.99	0.99	0.99	0.96	0.99	1.00	0.99	0.97	1.00	1.00	0.99	0.99
MAG_Ve_Sed_15	0.99	0.97	0.99	0.99	0.99	0.97	0.98	0.99	0.99	0.99	0.99	0.98	0.99	0.99	0.98	0.99	0.98	0.99	0.99	0.99	0.94	0.99	0.99	0.99	0.99
MAG_Ve_Sed_16	0.99	0.98	0.98	0.99	0.98	0.92	0.95	0.99	1.00	1.00	1.00	0.99	1.00	0.99	1.00	1.00	0.99	1.00	0.98	0.99	0.94	0.99	0.99	1.00	0.99
MAG_Ve_Sed_17	0.99	0.97	0.99	0.99	0.99	0.94	0.98	0.99	0.99	0.99	0.99	0.98	0.99	0.99	0.99	1.00	0.98	0.99	0.99	0.99	0.96	0.99	0.99	0.99	0.99
MAG_Ve_Sed_18	0.99	0.96	0.99	1.00	0.99	0.92	0.97	0.99	0.99	0.98	0.99	0.96	1.00	0.99	1.00	0.99	0.96	0.99	0.99	0.99	0.95	0.99	1.00	0.99	0.99
MAG_Ve_Sed_19	0.98	0.99	1.00	0.99	0.98	0.97	0.98	0.99	0.99	0.98	0.99	0.98	1.00	1.00	0.99	0.99	0.97	0.99	0.99	0.99	0.89	0.98	0.99	0.99	0.99
MAG_Ve_Sed_20	0.98	0.96	0.99	0.98	0.99	0.95	0.95	1.00	0.98	1.00	0.99	0.96	1.00	1.00	0.98	1.00	0.98	0.99	0.97	1.00	0.95	0.98	0.99	1.00	0.99
MAG_Ve_Sed_21	0.99	0.97	0.99	0.99	1.00	0.97	0.97	0.99	0.99	0.99	1.00	0.98	1.00	0.99	0.99	1.00	0.97	0.98	0.99	0.99	0.95	0.99	0.99	0.99	0.99
MAG_Ve_Sed_22	0.99	0.96	0.99	0.99	0.98	0.96	0.97	1.00	0.98	1.00	0.99	0.97	1.00	0.99	0.99	0.99	0.98	0.99	0.99	0.99	0.96	0.98	0.99	0.99	1.00
MAG_Ve_Sed_23	1.00	0.96	0.98	0.99	1.00	0.95	0.95	0.99	1.00	0.99	0.99	0.97	1.00	1.00	1.00	0.99	0.97	1.00	0.99	0.99	0.94	1.00	0.99	0.99	0.99
MAG_Ve_Sed_24	0.99	0.96	1.00	0.99	0.99	0.95	0.98	0.98	0.99	0.99	0.99	0.97	1.00	0.99	0.98	0.99	0.96	0.98	0.99	0.99	0.95	0.99	0.99	0.99	0.98
MAG_Ve_Sed_25	0.99	0.97	0.99	0.99	1.00	0.95	0.98	0.99	0.99	0.99	0.99	0.98	1.00	0.99	0.99	0.99	0.99	1.00	1.00	0.99	0.93	1.00	0.99	0.99	0.99
MAG_Ve_Sed_26	0.99	0.96	0.99	1.00	0.99	0.94	0.97	0.99	0.99	0.99	0.99	0.98	1.00	0.99	0.97	1.00	0.96	0.99	0.99	0.98	0.96	0.99	0.99	0.99	0.99
MAG_Ve_Sed_27	0.99	0.98	0.99	0.99	0.98	0.93	0.97	0.99	0.99	1.00	0.99	0.98	0.99	1.00	0.99	0.99	0.98	0.99	0.99	0.99	0.94	0.99	0.99	1.00	0.99
MAG_Ve_Sed_28	0.98	1.00	0.99	0.99	0.99	0.93	0.99	0.99	0.99	0.98	0.99	0.98	1.00	1.00	0.99	0.99	0.97	0.98	0.99	0.99	0.94	0.98	0.99	0.99	0.99
MAG_Ve_Sed_29	1.00	0.99	0.99	0.99	0.97	0.99	0.96	0.99	0.98	0.99	1.00	0.94	0.98	0.99	1.00	0.99	1.00	0.99	0.99	1.00	0.97	1.00	0.98	1.00	0.99
MAG_Ve_Sed_30	0.99	0.97	1.00	0.99	0.99	0.95	0.98	0.99	0.99	1.00	1.00	0.97	0.99	1.00	0.99	1.00	0.98	0.98	0.99	0.99	0.93	1.00	0.99	0.99	1.00
MAG_Ve_Sed_31	0.99	0.95	1.00	0.98	0.99	0.96	0.98	0.99	0.97	1.00	0.99	0.97	0.99	1.00	0.99	1.00	0.98	0.99	0.99	0.99	0.96	0.98	1.00	0.99	0.97
MAG_Ve_Sed_32	0.99	0.93	0.99	0.99	1.00	0.94	0.97	1.00	0.98	1.00	0.99	0.94	1.00	1.00	1.00	1.00	0.98	1.00	0.98	0.97	0.95	1.00	0.97	0.99	1.00
MAG_Ve_Sed_33	0.99	0.96	1.00	1.00	0.98	0.96	0.94	0.98	0.99	1.00	0.99	0.99	1.00	1.00	1.00	1.00	0.97	1.00	1.00	0.99	0.95	0.98	0.99	0.99	1.00
MAG_Ve_Sed_34	1.00	0.98	0.99	1.00	0.98	0.92	0.91	0.98	1.00	0.99	0.99	0.99	1.00	0.98	1.00	1.00	0.96	0.99	0.99	0.98	0.96	0.97	1.00	0.97	0.98
MAG_Ve_Sed_35	0.99	0.96	0.99	0.99	0.99	0.94	0.97	0.99	0.99	0.99	0.99	0.97	0.99	0.99	1.00	1.00	0.96	0.99	1.00	0.98	0.94	0.99	0.99	0.99	0.99
MAG_Ve_Sed_36	0.99	0.95	0.98	1.00	0.99	0.96	0.99	0.99	1.00	0.99	0.99	0.96	1.00	0.99	1.00	1.00	0.96	0.99	1.00	1.00	0.95	1.00	0.99	0.99	0.99
MAG_Ve_Sed_37	0.99	0.96	0.99	0.99	1.00	0.93	0.96	0.99	0.99	1.00	1.00	0.97	0.99	0.99	0.99	1.00	0.98	0.98	1.00	0.99	0.94	1.00	0.99	0.99	0.99
MAG_Ve_Sed_38	0.99	0.96	0.99	0.99	0.99	0.95	0.98	0.98	0.99	1.00	0.99	0.98	0.99	1.00	0.99	1.00	0.96	0.99	1.00	0.99	0.95	1.00	0.98	1.00	1.00
MAG_Ve_Sed_39	1.00	0.97	0.96	1.00	1.00	0.92	0.99	0.97	1.00	1.00	1.00	0.96	0.99	0.99	1.00	1.00	0.96	0.99	0.99	1.00	0.97	1.00	1.00	0.97	0.99
MAG_Ve_Sed_40	0.99	0.95	0.99	0.99	0.99	0.95	0.96	0.99	1.00	0.98	0.99	0.98	1.00	1.00	1.00	0.99	0.95	1.00	0.99	0.99	0.94	1.00	0.99	0.99	0.99
MAG_Ve_Sed_41	0.99	0.94	0.99	0.99	0.99	0.92	0.95	0.99	0.98	1.00	1.00	0.99	0.99	0.99	1.00	0.99	0.97	1.00	0.98	1.00	0.93	1.00	1.00	0.99	0.98
MAG_Ve_Sed_42	0.99	0.96	0.99	0.99	0.99	0.95	0.97	1.00	0.99	1.00	0.99	0.98	1.00	1.00	1.00	0.99	0.97	0.99	1.00	1.00	0.94	1.00	1.00	0.99	0.99
MAG_Ve_Sed_43	0.99	0.97	1.00	0.99	1.00	0.94	0.97	0.98	1.00	0.99	0.99	0.98	0.99	0.99	1.00	0.99	0.98	0.98	1.00	1.00	0.95	0.99	0.99	1.00	0.98
MAG_Ve_Sed_44	0.98	0.94	0.99	0.98	0.99	0.96	0.96	0.99	1.00	1.00	0.99	0.97	0.99	0.98	1.00	0.99	0.97	0.99	1.00	1.00	0.96	0.99	0.99	0.99	0.99
MAG_Ve_Sed_45	0.98	0.95	0.99	0.99	0.99	0.92	0.96	0.99	0.98	0.99	0.99	0.97	0.99	1.00	0.97	0.99	0.98	0.99	0.99	1.00	0.96	0.99	0.98	0.99	0.99
MAG_Ve_Sed_46	1.00	0.97	1.00	0.99	0.98	0.94	0.96	0.99	0.99	0.99	0.99	0.97	1.00	0.99	0.99	0.98	0.97	0.99	0.99	1.00	0.93	0.99	0.99	0.99	0.99
MAG_Ve_Sed_47	0.99	0.97	1.00	0.99	0.99	0.95	0.97	1.00	0.99	1.00	0.99	0.97	1.00	0.99	0.99	0.99	0.98	0.99	0.99	1.00	0.95	0.98	0.98	0.99	0.99
MAG_Ve_Sed_48	0.99	0.97	0.99	0.99	0.99	0.92	0.98	0.99	0.99	0.99	0.99	0.98	0.99	0.99	0.99	0.99	0.97	0.99	0.99	1.00	0.95	0.99	0.99	0.99	0.99
MAG_Ve_Sed_49	0.99	0.96	0.99	0.99	0.99	0.95	0.97	0.99	1.00	0.99	1.00	0.97	0.99	1.00	0.99	1.00	0.97	0.99	0.99	1.00	0.94	0.98	0.99	1.00	0.99
MAG_Ve_Sed_50	0.99	0.97	0.99	0.99	0.99	0.95	0.98	0.99	0.99	0.99	1.00	0.96	0.99	0.99	0.99	0.98	0.98	0.99	1.00	0.99	1.00	0.97	0.99	1.00	0.99
MAG_Ve_Sed_51	0.99	0.99	0.99	0.99	0.99	0.98	0.98	0.99	1.00	0.99	0.99	0.98	0.99	1.00	1.00	0.99	0.95	1.00	0.99	0.99	0.99	0.99	0.99	1.00	0.99

MAG_Ve_Sed_52	1.00	0.96	0.99	0.99	0.99	0.93	0.99	0.99	0.99	0.99	1.00	1.00	0.99	0.99	0.99	0.99	0.98	0.99	0.98	1.00	0.99	1.00	0.98	0.99	0.99
MAG_Ve_Sed_53	0.99	0.97	1.00	0.99	0.99	0.94	0.98	0.99	1.00	0.99	0.99	0.97	0.99	0.99	0.98	1.00	0.98	1.00	0.99	1.00	0.97	0.99	0.99	0.99	0.99
MAG_Ve_Sed_54	0.99	0.97	0.99	0.99	0.99	0.95	0.97	0.99	0.99	0.99	0.99	0.96	0.99	0.99	0.99	0.99	0.97	0.99	0.99	1.00	0.95	0.99	0.99	1.00	0.99
MAG_Ve_Sed_55	0.99	0.96	0.98	0.99	0.99	0.93	0.98	1.00	1.00	1.00	0.99	0.97	0.99	0.99	1.00	0.99	0.97	0.99	0.99	0.99	0.96	1.00	0.99	1.00	1.00
MAG_Ve_Sed_56	0.99	0.94	0.99	0.98	0.99	0.97	0.97	0.99	0.99	1.00	0.98	0.99	0.98	1.00	1.00	0.99	0.98	0.97	0.98	0.99	0.95	1.00	0.99	0.99	0.99
MAG_Ve_Sed_57	0.99	0.96	0.99	1.00	1.00	0.95	0.98	1.00	0.99	0.99	0.99	0.95	0.99	0.99	1.00	0.99	0.96	1.00	0.99	0.98	0.96	0.98	0.99	1.00	1.00

Supplementary Table S6 Relative abundance (average and standard deviation) of MAGs in the metagenomic assemblies. Bold numbers indicate significant differences ($q < 0.05$) among sites

MAG	C	C_sd	M	M_sd	P	P_sd	S	S_sd	T	T_sd
MAG_Ve_Sed_01	0.10731113	0.099752	0.012197	0.005036	0.009376	0.002872	0.010284	0.002703	0.008427	0.002286
MAG_Ve_Sed_02	0.12096782	0.065526	0.011074	0.00152	0.008274	0.002333	0.009645	0.003167	0.008542	0.000968
MAG_Ve_Sed_03	0.19390732	0.074385	0.012906	0.00413	0.008106	0.003897	0.008963	0.002298	0.008802	0.002977
MAG_Ve_Sed_04	0.11637399	0.113595	0.010403	0.002213	0.007886	0.003006	0.009296	0.002834	0.008667	0.001221
MAG_Ve_Sed_05	0.11372501	0.10009	0.012112	0.007056	0.009292	0.003643	0.009481	0.003023	0.009095	0.001846
MAG_Ve_Sed_06	0.0974655	0.072414	0.007518	0.001117	0.008703	0.00136	0.006016	0.001495	0.006173	0.002009
MAG_Ve_Sed_07	0.11476817	0.096469	0.01006	0.002622	0.009736	0.003431	0.010081	0.002445	0.009878	0.002934
MAG_Ve_Sed_08	0.10639819	0.086283	0.009362	0.002691	0.009152	0.003025	0.008381	0.002171	0.007444	0.001099
MAG_Ve_Sed_09	0.00845857	0.002128	0.104596	0.188452	0.014269	0.005601	0.010828	0.002285	0.018166	0.009115
MAG_Ve_Sed_10	0.01085183	0.00397	0.108701	0.087074	0.017681	0.006508	0.016034	0.001563	0.014333	0.002704
**MAG_Ve_Sed_11**	0.01293227	0.002895	0.02292	0.002674	**0.197655**	0.141657	**0.276928**	0.084626	0.019623	0.00513
**MAG_Ve_Sed_12**	0.0048114	0.001039	**0.29658**	0.269388	0.01366	0.007729	0.007951	0.003506	0.007728	0.002427
**MAG_Ve_Sed_13**	0.01081415	0.003797	**0.227099**	0.093742	0.017244	0.008347	**0.178951**	0.071758	0.015911	0.002753
MAG_Ve_Sed_14	0.29782442	0.14936	0.444682	0.44902	0.30641	0.404809	0.34396	0.289404	0.423581	0.315569
**MAG_Ve_Sed_15**	0.00998858	0.002724	**0.102896**	0.153751	0.021944	0.009225	0.016839	0.006896	0.018306	0.003875
**MAG_Ve_Sed_16**	0.004952	0.001533	0.009694	0.003224	**0.251785**	0.27995	0.009124	0.003251	0.006428	0.00137
**MAG_Ve_Sed_17**	0.00504674	0.001628	0.007891	0.001061	**0.121707**	0.111252	0.007969	0.001651	0.008829	0.002258
MAG_Ve_Sed_18	0.00446346	0.001518	0.008067	0.001491	0.297398	0.440156	0.006305	0.00239	0.006875	0.001562
**MAG_Ve_Sed_19**	0.02097273	0.005647	0.034621	0.002515	**0.787002**	0.576657	0.027482	0.004362	0.029381	0.006619
MAG_Ve_Sed_20	0.00433896	0.000989	0.009126	0.004308	0.377369	0.642934	0.005146	0.00162	0.005625	0.000896
MAG_Ve_Sed_21	0.00513026	0.001623	0.008526	0.001873	0.177278	0.1794	0.006503	0.001413	0.006261	0.00144
**MAG_Ve_Sed_22**	0.03950358	0.010125	0.06854	0.012341	**1.539791**	1.266364	0.230743	0.061517	0.36998	0.084772
**MAG_Ve_Sed_23**	0.00822778	0.002057	0.01578	0.001915	**0.188221**	0.177595	0.013969	0.004896	**0.146852**	0.042301
MAG_Ve_Sed_24	0.0047272	0.001558	0.009655	0.004053	0.204788	0.210772	0.007239	0.002615	0.007353	0.001917
MAG_Ve_Sed_25	0.00558733	0.003357	0.007828	0.002157	0.180485	0.142886	0.006426	0.001387	0.006195	0.00129
MAG_Ve_Sed_26	0.00992535	0.00429	0.01719	0.003626	0.289331	0.166719	0.013793	0.001911	0.016969	0.005034
**MAG_Ve_Sed_27**	0.0048652	0.001252	0.009429	0.002223	**0.158473**	0.149176	0.007475	0.002496	0.008028	0.001466
MAG_Ve_Sed_28	0.00534227	0.002041	0.010166	0.004313	0.226458	0.240278	0.006748	0.001846	0.006652	0.00142
MAG_Ve_Sed_29	0.17572045	0.136717	0.406279	0.318082	1.174266	0.764803	1.140496	0.28685	0.220447	0.08657
**MAG_Ve_Sed_30**	0.01916228	0.005274	**0.583541**	0.301719	0.041207	0.01203	0.163456	0.048848	0.033611	0.009252
**MAG_Ve_Sed_31**	0.00415626	0.001394	0.006163	0.001676	0.006518	0.001037	**0.135463**	0.045132	0.006941	0.002152
**MAG_Ve_Sed_32**	0.00400981	0.001478	0.008584	0.004191	0.007719	0.005979	**0.228131**	0.237559	0.005348	0.001463
**MAG_Ve_Sed_33**	0.01689433	0.003683	0.024821	0.004633	0.029085	0.012329	**0.713436**	0.295538	0.025959	0.007228
**MAG_Ve_Sed_34**	0.00473658	0.001133	0.006731	0.003963	0.010907	0.010118	**0.174675**	0.087321	0.006038	0.002354
**MAG_Ve_Sed_35**	0.01451815	0.004227	0.023386	0.004609	0.025089	0.007593	**0.494845**	0.165881	0.024148	0.007198
**MAG_Ve_Sed_36**	0.01805454	0.003787	0.028498	0.00445	0.23729	0.251814	**0.62515**	0.367762	0.027674	0.006329

**MAG_Ve_Sed_37**	0.00528423	0.003175	0.006948	0.002331	0.006458	0.002031	**0.269363**	0.314969	0.00674	0.00268
**MAG_Ve_Sed_38**	0.00546707	0.001705	0.007503	0.001132	0.009473	0.004016	**0.202171**	0.062934	0.00935	0.002124
**MAG_Ve_Sed_39**	0.01244837	0.006405	0.015028	0.002472	0.017962	0.00904	**0.334605**	0.11231	0.013703	0.003468
**MAG_Ve_Sed_40**	**0.10983845**	0.086356	0.020567	0.006077	0.019165	0.005371	**0.209556**	0.097915	0.019787	0.007645
**MAG_Ve_Sed_41**	0.00425781	0.00161	0.007487	0.004189	0.007677	0.002102	**0.16922**	0.052367	0.005946	0.00228
**MAG_Ve_Sed_42**	0.0043251	0.001348	0.00769	0.002441	0.007073	0.002411	**0.174389**	0.096253	0.0069	0.002237
**MAG_Ve_Sed_43**	0.02507083	0.007494	**0.495018**	0.222372	0.053754	0.014946	**0.410949**	0.251306	**0.341169**	0.143117
**MAG_Ve_Sed_44**	0.03182147	0.008212	0.055789	0.009208	0.059217	0.017716	**0.643472**	0.358872	**0.671761**	0.819733
**MAG_Ve_Sed_45**	0.00935287	0.002372	0.014898	0.002261	0.01733	0.005767	**0.313403**	0.111473	0.014316	0.004142
**MAG_Ve_Sed_46**	0.00717528	0.001075	0.014607	0.002795	0.016231	0.008385	**0.310062**	0.16741	0.011004	0.002929
**MAG_Ve_Sed_47**	0.00869333	0.001996	0.014147	0.005387	0.013439	0.005008	**0.295594**	0.116042	0.013789	0.004318
**MAG_Ve_Sed_48**	0.0042266	0.000894	0.006122	0.000976	0.007061	0.001792	**0.132627**	0.0479	0.00669	0.001739
**MAG_Ve_Sed_49**	0.00918374	0.003333	0.015492	0.003621	0.016313	0.007977	**0.3652**	0.207907	0.015398	0.005865
**MAG_Ve_Sed_50**	0.00839914	0.002404	0.012825	0.001911	0.011812	0.002871	0.016336	0.008715	**0.372937**	0.255063
**MAG_Ve_Sed_51**	0.01741528	0.003855	0.11075	0.069773	0.032048	0.012067	0.029294	0.005908	**0.286001**	0.144645
**MAG_Ve_Sed_52**	0.00392066	0.001109	0.008407	0.001384	0.007919	0.002963	0.00498	0.001328	**0.156875**	0.099016
**MAG_Ve_Sed_53**	0.01731804	0.010005	0.02411	0.003996	0.02592	0.006991	0.322558	0.10172	**0.141794**	0.048469
MAG_Ve_Sed_54	0.00602925	0.002081	0.013625	0.00309	0.011066	0.002496	0.010726	0.004916	0.151914	0.128845
**MAG_Ve_Sed_55**	0.01279068	0.004056	0.022785	0.000711	0.342066	0.296413	0.019863	0.006892	**0.181344**	0.07701
**MAG_Ve_Sed_56**	0.00412191	0.001368	0.008908	0.004999	0.006757	0.002396	0.006147	0.001953	**0.172663**	0.133581
MAG_Ve_Sed_57	0.00656313	0.002148	0.012879	0.004219	0.013184	0.004319	0.013493	0.014062	0.184088	0.189783
**MAG_Ve_Sed_58**	0.00386645	0.001156	0.008232	0.003232	0.006911	0.001711	0.005894	0.002082	**0.128807**	0.058235

Supplementary Table S7 Marker genes detected in the MAG dataset

Gene	Pathway	Metabolism	KO
PRK, prkB; phosphoribulokinase [EC:2.7.1.19]	Calvin Cycle	Carbon metabolism/autotrophic pathways	K00855
rbcL; ribulose-bisphosphate carboxylase large chain [EC:4.1.1.39]	Calvin Cycle	Carbon metabolism/autotrophic pathways	K01601
rbcS; ribulose-bisphosphate carboxylase small chain [EC:4.1.1.39]	Calvin Cycle	Carbon metabolism/autotrophic pathways	K01602
enoyl-CoA hydratase / 3-hydroxyacyl-CoA dehydrogenase [EC:4.2.1.17 1.1.1.35]	Dicarboxylate-hydroxybutyrate cycle | Hydroxypropionate-hydroxybutylate cycle	Carbon metabolism/autotrophic pathways	K15016
cooS, acsA; anaerobic carbon-monoxide dehydrogenase catalytic subunit [EC:1.2.7.4]	Reductive acetyl-CoA pathway (Wood-Ljungdahl pathway)	Carbon metabolism/autotrophic pathways	K00198
ackA; acetate kinase	Fermentation_acetate kinase	Central metabolism	K00925
adhP; alcohol dehydrogenase, propanol-preferring [EC:1.1.1.1]	Fermentation_alcohol dehydrogenase	Central metabolism	K13953
exaA; alcohol dehydrogenase (cytochrome c) [EC:1.1.2.8]	Fermentation_alcohol dehydrogenase	Central metabolism	K00114
aldh; aldehyde dehydrogenase (NAD+)	Fermentation_aldehyde dehydrogenase	Central metabolism	K00128
aldB; aldehyde dehydrogenase	Fermentation_aldehyde dehydrogenase	Central metabolism	K00138
adhE; acetaldehyde dehydrogenase / alcohol dehydrogenase	Fermentation_aldehyde dehydrogenase	Central metabolism	K04072
fdoG, fdfH; formate dehydrogenase major subunit	Fermentation_Formate dehydrogenase	Central metabolism	K00123
ldh; L-lactate dehydrogenase	Fermentation_Lactate dehydrogenase	Central metabolism	K00016
korA, oorA, oforA; 2-oxoglutarate/2-oxoacid ferredoxin oxidoreductase subunit alpha	Fermentation_PFOR/kor	Central metabolism	K00174
korB, oorB, oforB; 2-oxoglutarate/2-oxoacid ferredoxin oxidoreductase subunit beta	Fermentation_PFOR/kor	Central metabolism	K00175

porA; pyruvate ferredoxin oxidoreductase alpha subunit	Fermentation_PFOR/kor	Central metabolism	K00169
pta; phosphate acetyltransferase	Fermentation_Phosphate acetyltransferase	Central metabolism	K00625
fbp; fructose-1,6-bisphosphatase I	Gluconeogenesis_Fructose-1,6-bisphosphatase	Central metabolism	K03841
pckA; phosphoenolpyruvate carboxykinase (ATP)	Gluconeogenesis_Pyruvate Carboxylase	Central metabolism	K01610
glk; glucokinase	Glycolysis_Glucokinase	Central metabolism	K00845
pfKa; 6-phosphofructokinase	Glycolysis_Phosphofructokinase	Central metabolism	K00850
pyk; pyruvate kinase	Glycolysis_Pyruvate kinase	Central metabolism	K00873
coxS; aerobic carbon-monoxide dehydrogenase large subunit [EC:1.2.5.3]	CO-Oxidation	CO-Oxidation	K03518
coxM, cutM; aerobic carbon-monoxide dehydrogenase medium subunit [EC:1.2.5.3]	CO-Oxidation	CO-Oxidation	K03519
hoxH, NAD-reducing hydrogenase large subunit [EC:1.12.1.2]	H2 oxidation	H2 oxidation	K00436
fwdB, fmdB; formylmethanofuran dehydrogenase subunit B [EC:1.2.7.12]	CO2 => methane	Methane metabolism	K00201
fwdC, fmdC; formylmethanofuran dehydrogenase subunit C [EC:1.2.7.12]	CO2 => methane	Methane metabolism	K00202
mer; 5,10-methylenetetrahydromethanopterin reductase [EC:1.5.98.2]	CO2 => methane	Methane metabolism	K00320
ftr; formylmethanofuran--tetrahydromethanopterin N-formyltransferase [EC:2.3.1.101].	CO2 => methane	Methane metabolism	K00672
comB; 2-phosphosulfolactate phosphatase [EC:3.1.3.71]	Coenzyme M biosynthesis	Methane metabolism	K05979
comA; phosphosulfolactate synthase [EC:4.4.1.19]	Coenzyme M biosynthesis	Methane metabolism	K08097
hdrC; heterodisulfide reductase subunit C [EC:1.8.98.1]	methanol => methane |acetate => methane | methylamine/dimethylamine/trimethylamine => methane | CO2 => methane	Methane metabolism	K03390
mttB; trimethylamine---corrinoid protein Co-methyltransferase [EC:2.1.1.250]	methylamine/dimethylamine/trimethylamine => methane	Methane metabolism	K14083
narB; ferredoxin-nitrate reductase [EC:1.7.7.2]	Assimilatory nitrate reduction, nitrate => ammonia	Nitrogen metabolism	K00367
nasA; assimilatory nitrate reductase catalytic subunit [EC:1.7.99.4]	Assimilatory nitrate reduction, nitrate => ammonia	Nitrogen metabolism	K00372
nirK; nitrite reductase (NO-forming) [EC:1.7.2.1]	Denitrification, nitrate => nitrogen	Nitrogen metabolism	K00368
nosZ; nitrous-oxide reductase [EC:1.7.2.4]	Denitrification, nitrate => nitrogen	Nitrogen metabolism	K00376
norC; nitric oxide reductase subunit C	Denitrification, nitrate => nitrogen	Nitrogen metabolism	K02305
norB; nitric oxide reductase subunit B [EC:1.7.2.5]	Denitrification, nitrate => nitrogen	Nitrogen metabolism	K04561
nirS; nitrite reductase (NO-forming) / hydroxylamine reductase [EC:1.7.2.1 1.7.99.1]	Denitrification, nitrate => nitrogen	Nitrogen metabolism	K15864
narI, narV; nitrate reductase gamma subunit [EC:1.7.5.1 1.7.99.-]	Denitrification, nitrate => nitrogen|Dissimilatory nitrate reduction, nitrate => ammonia	Nitrogen metabolism	K00374
napA; periplasmic nitrate reductase NapA [EC:1.7.99.-]	Denitrification, nitrate => nitrogen|Dissimilatory nitrate reduction, nitrate => ammonia	Nitrogen metabolism	K02567
napB; cytochrome c-type protein NapB	Denitrification, nitrate => nitrogen|Dissimilatory nitrate reduction, nitrate => ammonia	Nitrogen metabolism	K02568
nirB; nitrite reductase (NADH) large subunit [EC:1.7.1.15]	Dissimilatory nitrate reduction, nitrate => ammonia	Nitrogen metabolism	K00362
nirD; nitrite reductase (NADH) small subunit [EC:1.7.1.15]	Dissimilatory nitrate reduction, nitrate => ammonia	Nitrogen metabolism	K00363
nrfA; nitrite reductase (cytochrome c-552) [EC:1.7.2.2]	Dissimilatory nitrate reduction, nitrate => ammonia	Nitrogen metabolism	K03385
narG, narZ, nxrA; nitrate reductase / nitrite oxidoreductase, alpha subunit [EC:1.7.5.1 1.7.99.-]	Nitrification, ammonia => nitrite => nitrate|Denitrification, nitrate => nitrogen|Dissimilatory nitrate reduction, nitrate => ammonia|	Nitrogen metabolism	K00370

narH, narY, nxrB; nitrate reductase / nitrite oxidoreductase, beta subunit [EC:1.7.5.1 1.7.99.-]	Nitrification, ammonia => nitrite => nitrate|Denitrification, nitrate => nitrogen|Dissimilatory nitrate reduction, nitrate => ammonia|	Nitrogen metabolism	K00371
nifH; nitrogenase iron protein NifH [EC:1.18.6.1]	Nitrogen fixation, nitrogen => ammonia	Nitrogen metabolism	K02588
ureA; urease subunit gamma [EC:3.5.1.5]	Urease	Nitrogen metabolism	K01428
ureB; urease subunit beta [EC:3.5.1.5]	Urease	Nitrogen metabolism	K01429
ureC; urease subunit alpha [EC:3.5.1.5]	Urease	Nitrogen metabolism	K01430
aprA; adenylylsulfate reductase, subunit A [EC:1.8.99.2]	Dissimilatory sulfate reduction, sulfate => H2S	Sulfur metabolism	K00394
aprB; adenylylsulfate reductase, subunit B [EC:1.8.99.2]	Dissimilatory sulfate reduction, sulfate => H2S	Sulfur metabolism	K00395
dsrA; dissimilatory sulfite reductase alpha subunit [EC:1.8.99.5]	Dissimilatory sulfate reduction, sulfate => H2S	Sulfur metabolism	K11180
dsrB; dissimilatory sulfite reductase beta subunit [EC:1.8.99.5]	Dissimilatory sulfate reduction, sulfate => H2S	Sulfur metabolism	K11181
soxA; sulfur-oxidizing protein SoxA	Thiosulfate oxidation by SOX complex, thiosulfate => sulfate	Sulfur metabolism	K17222
soxX; sulfur-oxidizing protein SoxX	Thiosulfate oxidation by SOX complex, thiosulfate => sulfate	Sulfur metabolism	K17223
soxB; sulfur-oxidizing protein SoxB	Thiosulfate oxidation by SOX complex, thiosulfate => sulfate	Sulfur metabolism	K17224
soxY; sulfur-oxidizing protein SoxY	Thiosulfate oxidation by SOX complex, thiosulfate => sulfate	Sulfur metabolism	K17226
soxZ; sulfur-oxidizing protein SoxZ	Thiosulfate oxidation by SOX complex, thiosulfate => sulfate	Sulfur metabolism	K17227

Supplementary Table S8 Output of the SIMPER analysis used to characterize the MAG clusters by the presence and distribution of genes related to different metabolisms and metabolic pathways.

Cluster1 (a) vs other clusters (b)	average	sd	ratio	ava	avb	cumsum	p
Calvin,Cycle_PRK,,prkB	0.03271	0.015389	2.1255	1	0.125	0.046	0.01
Dissimilatory,sulfate,reduction_dsrA	0.02778	0.018765	1	1	0.104	0.086	0.01
Thiosulfate,oxidation,by,SOX,complex_soxZ	0.02676	0.017564	2	0.8	0.104	0.124	0.01
Fermentation_Lactate,dehydrogenase_ldh	0.02464	0.017663	1	0.1	0.750	0.159	0.01
Fermentation_PFOR,kor_porA	0.0228	0.019172	1	0.8	0.292	0.192	0.02
Fermentation_aldehyde,dehydrogenase_aldh	0.02208	0.018672	1	0.1	0.646	0.223	0.02
Fermentation_PFOR,kor_korB	0.02077	0.018495	1	0.1	0.604	0.252	0.06
Calvin,Cycle_rbcL	0.02076	0.01856	1	0.6	0.063	0.282	0.01
H2,oxidation_hoxH	0.02068	0.019283	1	0.6	0.125	0.311	0.01
Fermentation_Phosphate,acetyltransferase_pta	0.02063	0.017486	1	0	0.604	0.341	0.05
Fermentation_PFOR,kor_korA	0.02042	0.018451	1	0.2	0.625	0.370	0.08
Glycolysis_Phosphofructokinase_pfKa	0.01912	0.019466	1	0.4	0.625	0.397	0.32
Gluconeogenesis_Pyruvate,Carboxylase_pcKA	0.01873	0.019136	0.9785	0	0.521	0.424	0.55
Fermentation_Formate,dehydrogenase_fdoG	0.01861	0.019499	0.9544	0.5	0.688	0.450	0.31
Fermentation_alcohol,dehydrogenase_adhP	0.01769	0.017726	0.9979	0	0.521	0.475	0.76
Thiosulfate,oxidation,by,SOX,complex_soxY	0.0173	0.018037	0.959	0.5	0.167	0.500	0.03
Dissimilatory,sulfate,reduction_aprB	0.01689	0.017542	0.9626	0.5	0.125	0.524	0.02
Dissimilatory,sulfate,reduction_aprA	0.01685	0.017525	0.9615	0.5	0.104	0.548	0.02
Thiosulfate,oxidation,by,SOX,complex_soxB	0.01672	0.017122	0.9763	0.5	0.250	0.571	0.12
Glycolysis_Glucokinase_glk	0.01629	0.020404	0.7982	1	0.583	0.595	0.89
Dissimilatory,sulfate,reduction_dsrB	0.01627	0.020303	0.8015	0	0.104	0.618	0.02
Gluconeogenesis_Fructose,1,6,bisphosphatase_fpb	0.0162	0.019958	0.8118	0.8	0.625	0.641	0.89
Fermentation_aldehyde,dehydrogenase_aldhE	0.01501	0.018711	0.8024	0.3	0.292	0.662	0.56
Fermentation_acetate,kinase_ackA	0.01445	0.017651	0.8188	0.1	0.396	0.683	0.96
Calvin,Cycle_rbcS	0.0142	0.018063	0.7863	0.4	0.021	0.703	0.01
CO,Oxidation,,C1,metabolism,_coxS	0.01405	0.017074	0.8227	0.1	0.396	0.723	0.99
CO,Oxidation,,C1,metabolism,_coxM,,cutM	0.01237	0.016688	0.7414	0.1	0.333	0.740	0.96
Denitrification,Dissimilatory,nitrate,reduction_napA	0.01208	0.016252	0.7435	0.3	0.167	0.758	0.26
Denitrification_norB	0.01136	0.016194	0.7017	0.3	0.104	0.774	0.07
Denitrification_nirS	0.01135	0.016189	0.7013	0.3	0.104	0.790	0.07
Denitrification_norC	0.0108	0.01599	0.6757	0.3	0.063	0.805	0.04
Urea,metabolism_ureC	0.01043	0.01585	0.6581	0.2	0.188	0.820	0.60
C1,metabolism,Methanogenesis,_mttB	0.01037	0.015746	0.6588	0	0.313	0.835	0.99
Urea,metabolism_ureA	0.00964	0.015574	0.6191	0.2	0.146	0.849	0.33
Thiosulfate,oxidation,by,SOX,complex_soxA	0.00878	0.014516	0.6051	0.2	0.125	0.861	0.40
C1,metabolism,Methanogenesis,_hdrC	0.00859	0.015402	0.5574	0.1	0.188	0.873	0.59
Assimilatory,nitrate,reduction_nasA	0.00821	0.01509	0.5439	0.2	0.063	0.885	0.14
Fermentation_aldehyde,dehydrogenase_aldB	0.00767	0.015366	0.4991	0	0.208	0.896	0.99
Nitrogen,fixation_nifH	0.00766	0.016008	0.4783	0.1	0.125	0.907	0.45
Denitrification,Dissimilatory,nitrate,reduction_napB	0.00731	0.013802	0.5293	0.2	0.042	0.917	0.02
Denitrification_nosZ	0.00589	0.013539	0.4348	0.1	0.083	0.925	0.62
Fermentation_alcohol,dehydrogenase_exaA	0.00479	0.01178	0.4064	0	0.146	0.932	0.95
Assimilatory,nitrate,reduction_narB	0.00428	0.011089	0.386	0.1	0.042	0.938	0.42
Coenzyme,M,biosynthesis_comB	0.00384	0.011497	0.3341	0	0.104	0.944	0.91
Dissimilatory,nitrate,reduction_nrfA	0.00377	0.011642	0.3242	0	0.104	0.949	0.91
Glycolysis_Pyruvate,kinase_pyk	0.00376	0.011327	0.3322	1	0.896	0.955	0.94
Reductive,acetyl,CoA,pathway,,Wood,Ljungdahl,pathway,_cooS,,acsA	0.00316	0.009403	0.3365	0	0.104	0.959	0.96
C1,metabolism,Methanogenesis,_ftr	0.00302	0.010383	0.2912	0	0.083	0.963	0.89
C1,metabolism,Methanogenesis,_mer	0.00296	0.010057	0.2944	0	0.083	0.968	0.87
Urea,metabolism_ureB	0.00272	0.009152	0.2973	0	0.083	0.971	0.87
C1,metabolism,Methanogenesis,_fwdC,,fmdC	0.00268	0.009003	0.2975	0	0.083	0.975	0.92
Nitrification,Denitrification,Dissimilatory,nitrate,reduction_narH,,narY,,nxrB	0.00256	0.008627	0.2971	0	0.083	0.979	0.93
Nitrification,Denitrification,Dissimilatory,nitrate,reduction_narG,,narZ,,nxrA	0.0025	0.008379	0.2978	0	0.083	0.982	0.96
Dicarboxylate,hydroxybutyrate,cycle,Hydroxypropionate,hydroxybutylate,cycle_enoyl,CoA,hydratase,,,3,hydroxyacyl,CoA,dehydrogenase	0.00245	0.008219	0.2984	0	0.083	0.986	0.94
Dissimilatory,nitrate,reduction_nirB	0.00217	0.008537	0.2544	0	0.063	0.989	0.85
Thiosulfate,oxidation,by,SOX,complex_soxX	0.00195	0.007638	0.2548	0	0.063	0.992	0.86
Denitrification,Dissimilatory,nitrate,reduction_narI,,narV	0.00191	0.007475	0.255	0	0.063	0.994	0.92
Coenzyme,M,biosynthesis_comA	0.00185	0.009084	0.2041	0	0.042	0.997	0.79
C1,metabolism,Methanogenesis,_fwdB,,fmdB	0.00069	0.004767	0.1444	0	0.021	0.998	0.82
Denitrification_nirK	0.00069	0.004767	0.1444	0	0.021	0.999	0.82
Dissimilatory,nitrate,reduction_nirD	0.00069	0.004767	0.1444	0	0.021	1.000	0.78

Cluster2 (a) vs other clusters (b)	average	sd	ratio	ava	avb	cumsum	p
Thiosulfate,oxidation,by,SOX,complex_soxB	0.028464	0.01453	1.959	1	0.180	0.044	0.01
CO,Oxidation,,C1,metabolism,_coxM,,cutM	0.024757	0.016306	2	1	0.200	0.083	0.01
Thiosulfate,oxidation,by,SOX,complex_soxA	0.024299	0.015707	2	0.75	0.040	0.120	0.01
CO,Oxidation,,C1,metabolism,_coxS	0.021161	0.017792	1	0.75	0.280	0.153	0.02
Gluconeogenesis_Fructose,1,6,bisphosphatase_fpb	0.020293	0.016778	1	0.25	0.720	0.185	0.12
C1,metabolism,Methanogenesis,_mttB	0.020175	0.018144	1	0.625	0.200	0.216	0.03

Glycolysis_Glucokinase_glk	0.020151	0.01672	1	0.25	0.720	0.248	0.13
Gluconeogenesis_Pyruvate,Carboxylase_pcKA	0.020048	0.016928	1	0.875	0.360	0.279	0.20
Thiosulfate,oxidation,by,SOX,complex_soxY	0.019438	0.017052	1	0.625	0.160	0.309	0.01
Fermentation_alcohol,dehydrogenase_adhP	0.019173	0.017744	1	0.75	0.380	0.339	0.22
Glycolysis_Phosphofructokinase_pfKa	0.019138	0.017284	1	0.25	0.640	0.369	0.35
Fermentation_PFOR,kor_korB	0.018101	0.016609	1	0.125	0.580	0.397	0.86
Fermentation_PFOR,kor_korA	0.017947	0.01674	1	0.25	0.600	0.425	0.85
Fermentation_Phosphate,acetyltransferase_pta	0.017883	0.017878	1	0.75	0.460	0.452	0.85
Fermentation_aldehyde,dehydrogenase_aldh	0.017661	0.017668	1	1	0.480	0.480	0.87
Denitrification_nirS	0.016723	0.017337	0.9646	1	0.080	0.506	0.01
Denitrification_norB	0.016276	0.01674	0.9723	0.5	0.080	0.531	0.01
Fermentation_Formate,dehydrogenase_fdoG	0.014679	0.018258	0.804	0.875	0.620	0.554	1.00
Fermentation_PFOR,kor_porA	0.014632	0.01665	0.8788	0.25	0.400	0.577	1.00
Fermentation_Lactate,dehydrogenase_ldh	0.014629	0.017628	0.8299	0.875	0.600	0.600	1.00
Fermentation_aldehyde,dehydrogenase_aldhE	0.014519	0.01672	0.8683	0.375	0.280	0.622	0.57
Fermentation_acetate,kinase_ackA	0.013298	0.016414	0.8102	0.125	0.380	0.643	1.00
Thiosulfate,oxidation,by,SOX,complex_soxZ	0.011306	0.01536	0.7361	0.25	0.220	0.660	0.59
Nitrogen,fixation_nifH	0.010108	0.015882	0.6365	0.25	0.100	0.676	0.24
Calvin,Cycle_PRK,,prkB	0.009896	0.014677	0.6742	0	0.320	0.692	1.00
Denitrification_norC	0.009558	0.015275	0.6257	0.25	0.080	0.706	0.14
H2,oxidation_hoxH	0.009333	0.014859	0.6281	0.125	0.220	0.721	0.91
Nitrification,Denitrification,Dissimilatory,nitrate,reduction_narG,,narZ,,nxrA	0.008894	0.014954	0.5947	0.25	0.040	0.735	0.02
Denitrification,Dissimilatory,nitrate,reduction_narI,,narV	0.00863	0.014875	0.5802	0.25	0.020	0.748	0.01
C1,metabolism,Methanogenesis,_fwdC,,fmdC	0.008499	0.014215	0.5979	0.25	0.040	0.761	0.10
Denitrification,Dissimilatory,nitrate,reduction_napB	0.008447	0.014128	0.5979	0.25	0.040	0.774	0.09
Dissimilatory,sulfate,reduction_aprB	0.008377	0.013827	0.6058	0.125	0.200	0.787	0.78
Denitrification,Dissimilatory,nitrate,reduction_napA	0.008327	0.013693	0.6081	0.125	0.200	0.800	0.81
C1,metabolism,Methanogenesis,_hdrC	0.008062	0.013817	0.5835	0.125	0.180	0.813	0.80
Thiosulfate,oxidation,by,SOX,complex_soxX	0.008057	0.013833	0.5824	0.25	0.020	0.826	0.07
Dissimilatory,sulfate,reduction_dsrA	0.008028	0.013787	0.5823	0	0.260	0.838	1.00
Urea,metabolism_ureC	0.006699	0.012748	0.5255	0	0.220	0.848	1.00
Fermentation_aldehyde,dehydrogenase_aldB	0.006678	0.013543	0.4931	0	0.200	0.859	1.00
Fermentation_alcohol,dehydrogenase_exaA	0.006552	0.01266	0.5175	0.125	0.120	0.869	0.67
Dissimilatory,nitrate,reduction_nrfA	0.006097	0.013113	0.4649	0.125	0.080	0.878	0.54
Dissimilatory,sulfate,reduction_aprA	0.00579	0.01168	0.4957	0	0.200	0.887	1.00
Assimilatory,nitrate,reduction_nasA	0.005755	0.012239	0.4702	0.125	0.080	0.896	0.56
Dissimilatory,sulfate,reduction_dsrB	0.005571	0.012077	0.4613	0	0.180	0.905	1.00
Nitrification,Denitrification,Dissimilatory,nitrate,reduction_narH,,narY,,nxrB	0.005569	0.012585	0.4425	0.125	0.060	0.914	0.29
Assimilatory,nitrate,reduction_narB	0.005509	0.013194	0.4175	0.125	0.040	0.922	0.18
Calvin,Cycle_rbcL	0.005432	0.011732	0.463	0	0.180	0.931	1.00
C1,metabolism,Methanogenesis,_ftr	0.005412	0.012188	0.444	0.125	0.060	0.939	0.42
Urea,metabolism_ureA	0.005329	0.011462	0.465	0	0.180	0.948	1.00
C1,metabolism,Methanogenesis,_mer	0.005287	0.011866	0.4455	0.125	0.060	0.956	0.52
Dicarboxylate,hydroxybutyrate,cycle,Hydroxypropionate,hydroxybutylate,cycle_enoyl ,CoA,hydratase,,,3,hydroxyacyl,CoA,dehydrogenase	0.004963	0.011088	0.4475	0.125	0.060	0.963	0.43
Denitrification_nosZ	0.003386	0.010287	0.3292	0	0.100	0.969	1.00
Coenzyme,M,biosynthesis_comB	0.003347	0.010138	0.3301	0	0.100	0.974	1.00
Glycolysis_Pyruvate,kinase_pyk	0.003282	0.009985	0.3287	1	0.900	0.979	1.00
Calvin,Cycle_rbcS	0.003003	0.009125	0.3291	0	0.100	0.984	1.00
Reductive,acetyl,CoA,pathway,,Wood,Ljungdahl,pathway,_cooS,,acsA	0.00281	0.008486	0.3311	0	0.100	0.988	1.00
Urea,metabolism_ureB	0.002401	0.008196	0.293	0	0.080	0.992	1.00
Dissimilatory,nitrate,reduction_nirB	0.001905	0.007591	0.251	0	0.060	0.995	1.00
Coenzyme,M,biosynthesis_comA	0.001581	0.007804	0.2025	0	0.040	0.997	1.00
C1,metabolism,Methanogenesis,_fwdB,,fmdB	0.000607	0.004266	0.1423	0	0.020	0.998	1.00
Denitrification_nirK	0.000607	0.004266	0.1423	0	0.020	0.999	1.00
Dissimilatory,nitrate,reduction_nirD	0.000607	0.004266	0.1423	0	0.020	1.000	1.00

**Cluster3 (a) vs other clusters (b)**	**average**	**sd**	**ratio**	**ava**	**avb**	**cumsum**	**p**
C1,metabolism,Methanogenesis,_hdrC	0.029708	0.010193	2.9146	1	0.077	0.049	0.01
Dissimilatory,sulfate,reduction_dsrB	0.020754	0.016435	1	1	0.096	0.083	0.01
Dissimilatory,sulfate,reduction_aprA	0.020686	0.016603	1	0.6667	0.115	0.117	0.01
Reductive,acetyl,CoA,pathway,,Wood,Ljungdahl,pathway,_cooS,,acsA	0.020274	0.015094	1	0.6667	0.019	0.151	0.01
Dissimilatory,sulfate,reduction_dsrA	0.019932	0.016635	1	0.6667	0.173	0.183	0.05
Dissimilatory,sulfate,reduction_aprB	0.019902	0.016081	1	0.6667	0.135	0.216	0.01
C1,metabolism,Methanogenesis,_mttB	0.019147	0.016434	1	0.6667	0.212	0.248	0.08
Fermentation_Phosphate,acetyltransferase_pta	0.018458	0.017149	1	1	0.442	0.278	0.64
Fermentation_acetate,kinase_ackA	0.01813	0.016496	1	1	0.308	0.308	0.31
Fermentation_aldehyde,dehydrogenase_aldh	0.01771	0.01602	1	0.1667	0.596	0.337	0.80
Fermentation_PFOR,kor_korB	0.017459	0.016873	1	1	0.462	0.366	0.94
Glycolysis_Pyruvate,kinase_pyk	0.016856	0.017362	1	1	0.962	0.394	0.01
Fermentation_PFOR,kor_korA	0.01647	0.017181	0.9587	1	0.500	0.421	0.98
Fermentation_aldehyde,dehydrogenase_aldhE	0.016221	0.016765	0.9676	1	0.269	0.448	0.34
Fermentation_PFOR,kor_porA	0.016084	0.016615	0.9681	0.5	0.365	0.474	0.84

Denitrification,Dissimilatory,nitrate,reduction_napA	0.015785	0.016234	0.9724	0.5	0.154	0.500	0.04
Fermentation_alcohol,dehydrogenase_adhP	0.01576	0.016138	0.9766	0.5	0.423	0.526	0.98
Nitrification,Denitrification,Dissimilatory,nitrate,reduction_narH,,narY,,nxrB	0.015656	0.01622	0.9652	0.5	0.019	0.552	0.01
Dicarboxylate,hydroxybutyrate,cycle,Hydroxypropionate,hydroxybutylate,cycle_enoyl,CoA,hydratase,,,3,hydroxyacyl,CoA,dehydrogenase	0.015545	0.015964	0.9738	0.5	0.019	0.577	0.01
Gluconeogenesis_Pyruvate,Carboxylase_pcKA	0.014989	0.01627	0.9213	0.1667	0.462	0.602	1.00
Glycolysis_Phosphofructokinase_pfKa	0.0146	0.016326	0.8942	0.8333	0.558	0.626	1.00
Fermentation_Lactate,dehydrogenase_ldh	0.013881	0.016688	0.8318	0.8333	0.615	0.649	1.00
CO,Oxidation,,C1,metabolism,_coxS	0.013863	0.015678	0.8842	0.3333	0.346	0.672	0.94
Gluconeogenesis_Fructose,1,6,bisphosphatase_fpb	0.013609	0.016748	0.8126	0.8333	0.635	0.694	0.99
Fermentation_Formate,dehydrogenase_fdoG	0.013312	0.017318	0.7687	1	0.615	0.716	1.00
Glycolysis_Glucokinase_glk	0.012868	0.016717	0.7697	1	0.615	0.737	1.00
CO,Oxidation,,C1,metabolism,_coxM,,cutM	0.011137	0.014669	0.7593	0	0.308	0.756	0.98
Nitrification,Denitrification,Dissimilatory,nitrate,reduction_narG,,narZ,,nxrA	0.010084	0.014072	0.7166	0.3333	0.039	0.772	0.06
Calvin,Cycle_PRK,,prkB	0.009117	0.013894	0.6562	0	0.308	0.787	1.00
Thiosulfate,oxidation,by,SOX,complex_soxB	0.009059	0.013101	0.6915	0	0.327	0.802	1.00
Nitrogen,fixation_nifH	0.007792	0.013992	0.5569	0.1667	0.115	0.815	0.59
Thiosulfate,oxidation,by,SOX,complex_soxZ	0.007189	0.012616	0.5698	0	0.250	0.827	1.00
Thiosulfate,oxidation,by,SOX,complex_soxY	0.007043	0.01231	0.5721	0	0.250	0.839	1.00
Dissimilatory,nitrate,reduction_nrfA	0.007031	0.013606	0.5168	0.1667	0.077	0.850	0.36
H2,oxidation_hoxH	0.00695	0.012854	0.5407	0	0.231	0.862	1.00
Thiosulfate,oxidation,by,SOX,complex_soxX	0.006629	0.013939	0.4756	0.1667	0.039	0.873	0.13
Urea,metabolism_ureC	0.006178	0.012046	0.5129	0	0.212	0.883	1.00
Fermentation_aldehyde,dehydrogenase_aldB	0.006132	0.01273	0.4817	0	0.192	0.893	1.00
Denitrification,Dissimilatory,nitrate,reduction_narI,,narV	0.005519	0.011442	0.4824	0.1667	0.039	0.902	0.25
Calvin,Cycle_rbcL	0.00501	0.011074	0.4524	0	0.173	0.910	1.00
Urea,metabolism_ureA	0.004921	0.010835	0.4542	0	0.173	0.918	1.00
Denitrification_norB	0.004305	0.010156	0.4239	0	0.154	0.925	1.00
Denitrification_nirS	0.004286	0.010129	0.4232	0	0.154	0.932	1.00
Thiosulfate,oxidation,by,SOX,complex_soxA	0.004264	0.010067	0.4236	0	0.154	0.939	1.00
Fermentation_alcohol,dehydrogenase_exaA	0.003895	0.009952	0.3913	0	0.135	0.946	1.00
Denitrification_norC	0.003274	0.009119	0.3591	0	0.115	0.951	1.00
Denitrification_nosZ	0.003107	0.00964	0.3224	0	0.096	0.956	1.00
Coenzyme,M,biosynthesis_comB	0.003074	0.00951	0.3232	0	0.096	0.961	1.00
Assimilatory,nitrate,reduction_nasA	0.002841	0.008793	0.3231	0	0.096	0.966	1.00
Calvin,Cycle_rbcS	0.00277	0.008595	0.3223	0	0.096	0.971	1.00
C1,metabolism,Methanogenesis,_ftr	0.002417	0.008528	0.2834	0	0.077	0.975	1.00
C1,metabolism,Methanogenesis,_mer	0.002379	0.008344	0.2851	0	0.077	0.979	1.00
Urea,metabolism_ureB	0.002216	0.007726	0.2869	0	0.077	0.982	1.00
C1,metabolism,Methanogenesis,_fwdC,,fmdC	0.002185	0.007618	0.2869	0	0.077	0.986	1.00
Denitrification,Dissimilatory,nitrate,reduction_napB	0.002104	0.007319	0.2874	0	0.077	0.989	1.00
Dissimilatory,nitrate,reduction_nirB	0.001754	0.007134	0.2459	0	0.058	0.992	1.00
Assimilatory,nitrate,reduction_narB	0.00167	0.006788	0.246	0	0.058	0.995	1.00
Coenzyme,M,biosynthesis_comA	0.00144	0.007253	0.1985	0	0.039	0.997	1.00
C1,metabolism,Methanogenesis,_fwdB,,fmdB	0.00056	0.004015	0.1395	0	0.019	0.998	1.00
Denitrification_nirK	0.00056	0.004015	0.1395	0	0.019	0.999	1.00
Dissimilatory,nitrate,reduction_nirD	0.00056	0.004015	0.1395	0	0.019	1.000	1.00

**Cluster4 (a) vs other clusters (b)**	**average**	**sd**	**ratio**	**ava**	**avb**	**cumsum**	**p**
Urea,metabolism_ureA	0.019746	0.016564	1.1921	0.625	0.080	0.033	0.01
Fermentation_alcohol,dehydrogenase_adhP	0.019599	0.01816	1	0.75	0.380	0.067	0.17
Glycolysis_Phosphofructokinase_pfKa	0.01958	0.01777	1	0.25	0.640	0.100	0.28
Urea,metabolism_ureC	0.019505	0.016715	1	0.625	0.120	0.133	0.02
Calvin,Cycle_PRK,,prkB	0.019438	0.017649	1	0.625	0.220	0.166	0.06
Fermentation_Phosphate,acetyltransferase_pta	0.017891	0.016729	1	0.125	0.560	0.196	0.88
Fermentation_aldehyde,dehydrogenase_aldh	0.017549	0.018348	1	0.875	0.500	0.226	0.90
Fermentation_alcohol,dehydrogenase_exaA	0.017533	0.018295	0.9584	0.5	0.060	0.256	0.01
C1,metabolism,Methanogenesis,_mttB	0.01746	0.01835	0.9515	0.5	0.220	0.285	0.04
Fermentation_PFOR,kor_porA	0.017341	0.018059	0.9602	0.5	0.360	0.314	0.64
Fermentation_PFOR,kor_korB	0.017273	0.018018	0.9587	0.5	0.520	0.344	0.96
Fermentation_PFOR,kor_korA	0.017261	0.018015	0.9581	0.5	0.560	0.373	0.95
CO,Oxidation,,C1,metabolism,_coxS	0.01703	0.017736	0.9602	0.5	0.320	0.402	0.39
Gluconeogenesis_Pyruvate,Carboxylase_pcKA	0.016398	0.017718	0.9255	0.25	0.460	0.430	0.98
CO,Oxidation,,C1,metabolism,_coxM,,cutM	0.015233	0.017688	0.8612	0.375	0.280	0.455	0.39
Fermentation_Formate,dehydrogenase_fdoG	0.015182	0.018928	0.8021	0.875	0.620	0.481	0.97
Fermentation_Lactate,dehydrogenase_ldh	0.015143	0.018295	0.8277	0.875	0.600	0.507	0.99
Gluconeogenesis_Fructose,1,6,bisphosphatase_fpb	0.014692	0.018329	0.8015	0.875	0.620	0.532	0.97
Thiosulfate,oxidation,by,SOX,complex_soxB	0.014556	0.016675	0.8729	0.375	0.280	0.556	0.52
Glycolysis_Glucokinase_glk	0.014554	0.018457	0.7885	1	0.600	0.581	0.96
Fermentation_acetate,kinase_ackA	0.014393	0.017007	0.8463	0	0.360	0.605	0.92
Fermentation_aldehyde,dehydrogenase_aldB	0.014255	0.017668	0.8068	0.375	0.140	0.629	0.15
Fermentation_aldehyde,dehydrogenase_aldhE	0.012428	0.017029	0.7298	0.125	0.320	0.650	0.93
Urea,metabolism_ureB	0.01242	0.01623	0.7653	0.375	0.020	0.671	0.01
Thiosulfate,oxidation,by,SOX,complex_soxY	0.011844	0.016205	0.7309	0.25	0.220	0.692	0.48

Thiosulfate,oxidation,by,SOX,complex_soxZ	0.01173	0.016052	0.7307	0.25	0.220	0.711	0.55
Calvin,Cycle_rbcL	0.010691	0.015955	0.6701	0.25	0.140	0.730	0.32
Nitrogen,fixation_nifH	0.010125	0.015852	0.6387	0.25	0.100	0.747	0.24
Dissimilatory,sulfate,reduction_dsrA	0.009736	0.014999	0.6491	0.125	0.240	0.763	0.96
H2,oxidation_hoxH	0.00968	0.015478	0.6254	0.125	0.220	0.780	0.89
Dissimilatory,sulfate,reduction_aprB	0.008302	0.01363	0.6091	0.125	0.200	0.794	0.92
C1,metabolism,Methanogenesis,_hdrC	0.008283	0.014226	0.5823	0.125	0.180	0.808	0.84
Dissimilatory,sulfate,reduction_aprA	0.0079	0.013485	0.5859	0.125	0.180	0.821	0.82
Dissimilatory,sulfate,reduction_dsrB	0.007749	0.01386	0.5591	0.125	0.160	0.834	0.77
Denitrification_nirS	0.006974	0.012909	0.5403	0.125	0.140	0.846	0.71
Denitrification,Dissimilatory,nitrate,reduction_napA	0.006607	0.012581	0.5252	0	0.220	0.857	1.00
Coenzyme,M,biosynthesis_comB	0.006437	0.013755	0.468	0.125	0.080	0.868	0.53
C1,metabolism,Methanogenesis,_mer	0.006424	0.014643	0.4387	0.125	0.060	0.879	0.30
Reductive,acetyl,CoA,pathway,,Wood,Ljungdahl,pathway,_cooS,,acsA	0.006136	0.013216	0.4643	0.125	0.080	0.889	0.26
Dissimilatory,nitrate,reduction_nirB	0.005318	0.012635	0.4209	0.125	0.040	0.898	0.35
Assimilatory,nitrate,reduction_narB	0.005241	0.012467	0.4204	0.125	0.040	0.907	0.28
Denitrification_norB	0.004789	0.011054	0.4332	0	0.160	0.915	1.00
Thiosulfate,oxidation,by,SOX,complex_soxA	0.004741	0.01095	0.4329	0	0.160	0.923	1.00
Dissimilatory,nitrate,reduction_nirD	0.004313	0.011622	0.3711	0.125	0.000	0.931	0.01
Denitrification_norC	0.003646	0.009945	0.3666	0	0.120	0.937	0.99
Denitrification_nosZ	0.003499	0.010654	0.3285	0	0.100	0.943	0.98
Glycolysis_Pyruvate,kinase_pyk	0.003388	0.010331	0.328	1	0.900	0.949	0.97
Dissimilatory,nitrate,reduction_nrfA	0.003387	0.010517	0.3221	0	0.100	0.954	0.98
Assimilatory,nitrate,reduction_nasA	0.003175	0.009635	0.3295	0	0.100	0.960	0.99
Calvin,Cycle_rbcS	0.003091	0.009409	0.3285	0	0.100	0.965	0.99
C1,metabolism,Methanogenesis,_ftr	0.002718	0.009432	0.2882	0	0.080	0.970	0.96
C1,metabolism,Methanogenesis,_fwdC,,fmdC	0.002434	0.008317	0.2926	0	0.080	0.974	0.96
Denitrification,Dissimilatory,nitrate,reduction_napB	0.002336	0.007963	0.2933	0	0.080	0.978	0.99
Nitrification,Denitrification,Dissimilatory,nitrate,reduction_narH,,narY,,nxrB	0.002335	0.007991	0.2922	0	0.080	0.982	0.97
Nitrification,Denitrification,Dissimilatory,nitrate,reduction_narG,,narZ,,nxrA	0.002278	0.00778	0.2928	0	0.080	0.985	0.97
Dicarboxylate,hydroxybutyrate,cycle,Hydroxypropionate,hydroxybutylate,cycle_enoyl ,CoA,hydratase,,,3,hydroxyacyl,CoA,dehydrogenase	0.002241	0.007643	0.2932	0	0.080	0.989	1.00
Thiosulfate,oxidation,by,SOX,complex_soxX	0.001772	0.007069	0.2507	0	0.060	0.992	0.97
Denitrification,Dissimilatory,nitrate,reduction_narI,,narV	0.001738	0.006929	0.2508	0	0.060	0.995	0.97
Coenzyme,M,biosynthesis_comA	0.001643	0.008128	0.2021	0	0.040	0.998	0.96
C1,metabolism,Methanogenesis,_fwdB,,fmdB	0.000625	0.004394	0.1422	0	0.020	0.999	0.95
Denitrification_nirK	0.000625	0.004394	0.1422	0	0.020	1.000	0.95

**Cluster5 (a) vs other clusters (b)**	**average**	**sd**	**ratio**	**ava**	**avb**	**cumsum**	**p**
Fermentation_PFOR,kor_korA	0.031322	0.025956	1.2068	0	0.615	0.044	0.01
Gluconeogenesis_Fructose,1,6,bisphosphatase_fpb	0.028664	0.026469	1	0.3333	0.692	0.085	0.01
Glycolysis_Glucokinase_glk	0.028383	0.026159	1	0.3333	0.692	0.125	0.01
Fermentation_Formate,dehydrogenase_fdoG	0.028321	0.025763	1	0.3333	0.692	0.165	0.02
Fermentation_Phosphate,acetyltransferase_pta	0.027737	0.025778	1	0	0.558	0.204	0.01
Fermentation_PFOR,kor_korB	0.027193	0.026104	1	0.1667	0.558	0.243	0.01
Fermentation_aldehyde,dehydrogenase_aldh	0.026359	0.026075	1	0.3333	0.577	0.280	0.01
Fermentation_Lactate,dehydrogenase_ldh	0.025439	0.026421	1	0.5	0.654	0.316	0.01
Glycolysis_Phosphofructokinase_pfKa	0.02534	0.026374	0.9608	0.5	0.596	0.352	0.01
Gluconeogenesis_Pyruvate,Carboxylase_pcKA	0.024384	0.026337	0.9258	0.3333	0.442	0.387	0.01
Fermentation_alcohol,dehydrogenase_adhP	0.023146	0.025175	0.9194	0.1667	0.462	0.420	0.02
Fermentation_PFOR,kor_porA	0.021953	0.026068	0.8421	0.1667	0.404	0.450	0.04
Fermentation_aldehyde,dehydrogenase_aldhE	0.02137	0.025476	0.8388	0.3333	0.289	0.481	0.02
Fermentation_acetate,kinase_ackA	0.019316	0.0252	0.7665	0	0.385	0.508	0.22
CO,Oxidation,,C1,metabolism,_coxS	0.018372	0.023787	0.7724	0	0.385	0.534	0.25
CO,Oxidation,,C1,metabolism,_coxM,,cutM	0.015721	0.023087	0.6809	0	0.327	0.556	0.41
Calvin,Cycle_PRK,,prkB	0.015128	0.023523	0.6431	0	0.308	0.578	0.30
Thiosulfate,oxidation,by,SOX,complex_soxB	0.014262	0.020697	0.6891	0	0.327	0.598	0.45
Fermentation_aldehyde,dehydrogenase_aldB	0.014075	0.023002	0.6119	0.1667	0.173	0.618	0.27
C1,metabolism,Methanogenesis,_mttB	0.013743	0.022009	0.6244	0	0.289	0.637	0.55
Dissimilatory,sulfate,reduction_dsrA	0.012294	0.022125	0.5557	0	0.250	0.655	0.44
Thiosulfate,oxidation,by,SOX,complex_soxZ	0.011669	0.020764	0.562	0	0.250	0.671	0.40
H2,oxidation_hoxH	0.011628	0.021855	0.5321	0	0.231	0.688	0.46
Nitrogen,fixation_nifH	0.011607	0.020925	0.5547	0.1667	0.115	0.704	0.14
Thiosulfate,oxidation,by,SOX,complex_soxY	0.011224	0.019746	0.5684	0	0.250	0.720	0.48
Dissimilatory,nitrate,reduction_nrfA	0.010879	0.021136	0.5147	0.1667	0.077	0.736	0.06
Urea,metabolism_ureC	0.01008	0.019819	0.5086	0	0.212	0.750	0.69
C1,metabolism,Methanogenesis,_ftr	0.009582	0.0192	0.499	0.1667	0.058	0.763	0.03
Denitrification,Dissimilatory,nitrate,reduction_napA	0.009444	0.018529	0.5097	0	0.212	0.777	0.64
Dissimilatory,sulfate,reduction_aprB	0.009139	0.017875	0.5113	0	0.212	0.790	0.69
C1,metabolism,Methanogenesis,_hdrC	0.008657	0.017989	0.4813	0	0.192	0.802	0.62
Coenzyme,M,biosynthesis_comA	0.008624	0.018914	0.456	0.1667	0.019	0.814	0.10
Dissimilatory,sulfate,reduction_dsrB	0.008535	0.019304	0.4422	0	0.173	0.826	0.63
Dissimilatory,sulfate,reduction_aprA	0.008462	0.017584	0.4812	0	0.192	0.838	0.63

Calvin,Cycle_rbcL	0.008155	0.018241	0.4471	0	0.173	0.850	0.61
Urea,metabolism_ureA	0.007871	0.017398	0.4524	0	0.173	0.861	0.74
Denitrification_norB	0.006809	0.016097	0.423	0	0.154	0.871	0.54
Denitrification_nirS	0.00678	0.016092	0.4213	0	0.154	0.880	0.69
Thiosulfate,oxidation,by,SOX,complex_soxA	0.006714	0.015896	0.4224	0	0.154	0.890	0.64
Fermentation_alcohol,dehydrogenase_exaA	0.006303	0.016186	0.3894	0	0.135	0.898	0.56
Denitrification_nosZ	0.005468	0.017203	0.3179	0	0.096	0.906	0.37
Coenzyme,M,biosynthesis_comB	0.005332	0.016633	0.3206	0	0.096	0.914	0.56
Denitrification_norC	0.005222	0.014569	0.3584	0	0.115	0.921	0.47
Glycolysis_Pyruvate,kinase_pyk	0.005209	0.016468	0.3163	1	0.904	0.929	0.39
Assimilatory,nitrate,reduction_nasA	0.004675	0.014584	0.3205	0	0.096	0.935	0.38
Calvin,Cycle_rbcS	0.004511	0.014203	0.3176	0	0.096	0.942	0.32
C1,metabolism,Methanogenesis,_mer	0.004059	0.014404	0.2818	0	0.077	0.947	0.37
Reductive,acetyl,CoA,pathway,,Wood,Ljungdahl,pathway,_cooS,,acsA	0.004039	0.012501	0.3231	0	0.096	0.953	0.40
Urea,metabolism_ureB	0.00357	0.012486	0.286	0	0.077	0.958	0.44
C1,metabolism,Methanogenesis,_fwdC,,fmdC	0.003491	0.0122	0.2861	0	0.077	0.963	0.32
Nitrification,Denitrification,Dissimilatory,nitrate,reduction_narH,,narY,,nxrB	0.003291	0.011538	0.2852	0	0.077	0.968	0.37
Denitrification,Dissimilatory,nitrate,reduction_napB	0.003278	0.011402	0.2875	0	0.077	0.972	0.32
Nitrification,Denitrification,Dissimilatory,nitrate,reduction_narG,,narZ,,nxrA	0.003168	0.011067	0.2863	0	0.077	0.977	0.39
Dicarboxylate,hydroxybutyrate,cycle,Hydroxypropionate,hydroxybutylate,cycle_enoyl ,CoA,hydratase,,,3,hydroxyacyl,CoA,dehydrogenase	0.00309	0.010764	0.287	0	0.077	0.981	0.28
Dissimilatory,nitrate,reduction_nirB	0.002924	0.011927	0.2452	0	0.058	0.985	0.34
Assimilatory,nitrate,reduction_narB	0.002696	0.010982	0.2455	0	0.058	0.989	0.29
Thiosulfate,oxidation,by,SOX,complex_soxX	0.002508	0.010228	0.2452	0	0.058	0.993	0.27
Denitrification,Dissimilatory,nitrate,reduction_narI,,narV	0.002436	0.009929	0.2453	0	0.058	0.996	0.28
C1,metabolism,Methanogenesis,_fwdB,,fmdB	0.000904	0.00648	0.1396	0	0.019	0.997	0.10
Denitrification_nirK	0.000904	0.00648	0.1396	0	0.019	0.999	0.11
Dissimilatory,nitrate,reduction_nirD	0.000904	0.00648	0.1396	0	0.019	1.000	0.13

**Cluster6 (a) vs other clusters (b)**	**average**	**sd**	**ratio**	**ava**	**avb**	**cumsum**	**p**
Fermentation_alcohol,dehydrogenase_adhP	0.02498	0.021512	1.1612	0.875	0.360	0.042	0.01
Fermentation_PFOR,kor_korA	0.022386	0.023192	1	1	0.480	0.080	0.03
Fermentation_Lactate,dehydrogenase_ldh	0.021938	0.021172	1.0362	0	0.680	0.116	0.08
Fermentation_PFOR,kor_korB	0.021748	0.021813	1	0.875	0.460	0.153	0.04
CO,Oxidation,,C1,metabolism,_coxS	0.02162	0.020566	1.0512	0.625	0.300	0.189	0.02
Fermentation_Formate,dehydrogenase_fdoG	0.021491	0.020574	1	0.375	0.700	0.226	0.06
Gluconeogenesis_Pyruvate,Carboxylase_pcKA	0.02085	0.02105	1	0.625	0.400	0.261	0.06
Glycolysis_Glucokinase_glk	0.020066	0.021231	0.9451	0.5	0.680	0.294	0.18
Denitrification_nosZ	0.020043	0.021345	0.939	0.5	0.020	0.328	0.01
Fermentation_aldehyde,dehydrogenase_aldh	0.019997	0.021175	0.9444	0.5	0.560	0.362	0.28
Fermentation_Phosphate,acetyltransferase_pta	0.01997	0.02118	0.9428	0.5	0.500	0.395	0.21
Glycolysis_Phosphofructokinase_pfKa	0.018643	0.021008	0.8874	0.875	0.540	0.427	0.44
Fermentation_PFOR,kor_porA	0.017903	0.021047	0.8506	0.25	0.400	0.457	0.46
Gluconeogenesis_Fructose,1,6,bisphosphatase_fpb	0.017406	0.022461	0.775	1	0.600	0.486	0.57
CO,Oxidation,,C1,metabolism,_coxM,,cutM	0.017157	0.019975	0.8589	0	0.280	0.515	0.13
Fermentation_acetate,kinase_ackA	0.016802	0.020327	0.8266	0.25	0.360	0.543	0.39
Fermentation_aldehyde,dehydrogenase_aldhE	0.014207	0.019627	0.7238	0.125	0.320	0.567	0.73
H2,oxidation_hoxH	0.013274	0.018683	0.7105	0.25	0.200	0.589	0.26
Calvin,Cycle_PRK,,prkB	0.011927	0.017826	0.6691	0	0.320	0.609	0.92
Thiosulfate,oxidation,by,SOX,complex_soxB	0.011616	0.01636	0.71	0	0.340	0.629	0.99
C1,metabolism,Methanogenesis,_mttB	0.010955	0.017004	0.6443	0	0.300	0.647	0.95
C1,metabolism,Methanogenesis,_mer	0.010452	0.01786	0.5852	0.25	0.040	0.665	0.02
Urea,metabolism_ureC	0.01003	0.016612	0.6038	0.125	0.200	0.682	0.53
Denitrification,Dissimilatory,nitrate,reduction_napA	0.010016	0.016688	0.6002	0.125	0.200	0.699	0.50
Dissimilatory,sulfate,reduction_dsrA	0.00968	0.016758	0.5776	0	0.260	0.715	0.94
Thiosulfate,oxidation,by,SOX,complex_soxZ	0.009326	0.016048	0.5811	0	0.260	0.731	0.91
C1,metabolism,Methanogenesis,_hdrC	0.009214	0.015817	0.5825	0.125	0.180	0.746	0.53
Thiosulfate,oxidation,by,SOX,complex_soxY	0.009074	0.015509	0.5851	0	0.260	0.761	0.94
Fermentation_aldehyde,dehydrogenase_aldB	0.008179	0.016698	0.4899	0	0.200	0.775	0.85
Glycolysis_Pyruvate,kinase_pyk	0.00799	0.017536	0.4556	0.875	0.920	0.788	0.25
Fermentation_alcohol,dehydrogenase_exaA	0.007705	0.014993	0.5139	0.125	0.120	0.801	0.25
Coenzyme,M,biosynthesis_comB	0.007659	0.016565	0.4623	0.125	0.080	0.814	0.14
Dissimilatory,sulfate,reduction_aprB	0.007453	0.014189	0.5253	0	0.220	0.827	0.88
Assimilatory,nitrate,reduction_nasA	0.007207	0.015571	0.4628	0.125	0.080	0.839	0.17
Dissimilatory,sulfate,reduction_aprA	0.006872	0.013904	0.4943	0	0.200	0.851	0.90
Dissimilatory,sulfate,reduction_dsrB	0.006718	0.014668	0.458	0	0.180	0.862	0.86
Calvin,Cycle_rbcL	0.006507	0.014123	0.4607	0	0.180	0.873	0.81
Urea,metabolism_ureA	0.00635	0.013692	0.4638	0	0.180	0.883	0.85
Denitrification_norB	0.005531	0.012774	0.433	0	0.160	0.893	0.75
Nitrogen,fixation_nifH	0.005509	0.014043	0.3923	0	0.140	0.902	0.75
Denitrification_nirS	0.005506	0.012749	0.4319	0	0.160	0.911	0.82
Thiosulfate,oxidation,by,SOX,complex_soxA	0.005468	0.01264	0.4326	0	0.160	0.920	0.80
Denitrification_norC	0.004221	0.011519	0.3664	0	0.120	0.927	0.68
Dissimilatory,nitrate,reduction_nrfA	0.004035	0.012698	0.3177	0	0.100	0.934	0.58

Calvin,Cycle_rbcS	0.003597	0.01099	0.3273	0	0.100	0.940	0.65
Reductive,acetyl,CoA,pathway,,Wood,Ljungdahl,pathway,_cooS,,acsA	0.003315	0.010032	0.3305	0	0.100	0.946	0.57
C1,metabolism,Methanogenesis,_ftr	0.003226	0.011277	0.2861	0	0.080	0.951	0.53
Urea,metabolism_ureB	0.002868	0.009812	0.2924	0	0.080	0.956	0.59
C1,metabolism,Methanogenesis,_fwdC,,fmdC	0.002819	0.009639	0.2924	0	0.080	0.961	0.56
Nitrification,Denitrification,Dissimilatory,nitrate,reduction_narH,,narY,,nxrB	0.002688	0.009212	0.2919	0	0.080	0.965	0.56
Denitrification,Dissimilatory,nitrate,reduction_napB	0.002686	0.009158	0.2933	0	0.080	0.970	0.58
Nitrification,Denitrification,Dissimilatory,nitrate,reduction_narG,,narZ,,nxrA	0.002612	0.008926	0.2926	0	0.080	0.974	0.52
Dicarboxylate,hydroxybutyrate,cycle,Hydroxypropionate,hydroxybutylate,cycle_enoyl ,CoA,hydratase,,,3,hydroxyacyl,CoA,dehydrogenase	0.002562	0.008741	0.2931	0	0.080	0.979	0.55
Dissimilatory,nitrate,reduction_nirB	0.002302	0.009191	0.2505	0	0.060	0.983	0.34
Assimilatory,nitrate,reduction_narB	0.002163	0.00863	0.2507	0	0.060	0.986	0.45
Thiosulfate,oxidation,by,SOX,complex_soxX	0.002044	0.008157	0.2505	0	0.060	0.990	0.48
Coenzyme,M,biosynthesis_comA	0.002014	0.00998	0.2018	0	0.040	0.993	0.41
Denitrification,Dissimilatory,nitrate,reduction_narI,,narV	0.001998	0.007971	0.2506	0	0.060	0.996	0.43
C1,metabolism,Methanogenesis,_fwdB,,fmdB	0.000726	0.005106	0.1422	0	0.020	0.998	0.33
Denitrification_nirK	0.000726	0.005106	0.1422	0	0.020	0.999	0.26
Dissimilatory,nitrate,reduction_nirD	0.000726	0.005106	0.1422	0	0.020	1.000	0.21

**Cluster7 (a) vs other clusters (b)**	**average**	**sd**	**ratio**	**ava**	**avb**	**cumsum**	**p**
Fermentation_acetate,kinase_ackA	0.026	0.019	1.368	0.833	0.217	0.043	0.01
Fermentation_Phosphate,acetyltransferase_pta	0.025	0.021	1.211	1.000	0.370	0.085	0.01
Fermentation_PFOR,kor_korB	0.021	0.020	1.025	0.833	0.435	0.119	0.10
Gluconeogenesis_Pyruvate,Carboxylase_pcKA	0.020	0.020	1.028	0.667	0.370	0.153	0.04
Fermentation_PFOR,kor_korA	0.020	0.020	0.970	0.833	0.478	0.186	0.14
Fermentation_aldehyde,dehydrogenase_aldB	0.019	0.020	0.948	0.500	0.087	0.218	0.01
Fermentation_aldehyde,dehydrogenase_aldh	0.019	0.020	0.948	0.750	0.500	0.250	0.55
Gluconeogenesis_Fructose,1,6,bisphosphatase_fpb	0.019	0.020	0.953	0.500	0.696	0.281	0.16
Glycolysis_Glucokinase_glk	0.019	0.020	0.949	0.500	0.696	0.312	0.19
Glycolysis_Phosphofructokinase_pfKa	0.018	0.019	0.946	0.917	0.500	0.343	0.53
Fermentation_alcohol,dehydrogenase_adhP	0.018	0.018	0.969	0.167	0.500	0.372	0.67
Fermentation_Lactate,dehydrogenase_ldh	0.017	0.020	0.851	0.917	0.565	0.402	0.72
Fermentation_Formate,dehydrogenase_fdoG	0.017	0.020	0.864	0.667	0.652	0.430	0.63
Fermentation_PFOR,kor_porA	0.017	0.019	0.875	0.167	0.435	0.458	0.88
Fermentation_aldehyde,dehydrogenase_aldhE	0.015	0.019	0.825	0.333	0.283	0.484	0.49
CO,Oxidation,,C1,metabolism,_coxS	0.015	0.018	0.843	0.167	0.391	0.509	0.95
CO,Oxidation,,C1,metabolism,_coxM,,cutM	0.014	0.018	0.765	0.167	0.326	0.531	0.90
C1,metabolism,Methanogenesis,_mttB	0.013	0.018	0.717	0.167	0.283	0.552	0.78
Urea,metabolism_ureC	0.013	0.018	0.683	0.250	0.174	0.573	0.09
Calvin,Cycle_PRK,,prkB	0.012	0.017	0.724	0.083	0.326	0.594	0.98
Thiosulfate,oxidation,by,SOX,complex_soxB	0.012	0.016	0.763	0.083	0.348	0.614	1.00
Denitrification,Dissimilatory,nitrate,reduction_napA	0.011	0.017	0.693	0.250	0.174	0.633	0.20
H2,oxidation_hoxH	0.011	0.017	0.653	0.167	0.217	0.652	0.63
Coenzyme,M,biosynthesis_comB	0.010	0.018	0.589	0.250	0.044	0.669	0.05
Thiosulfate,oxidation,by,SOX,complex_soxZ	0.010	0.016	0.643	0.083	0.261	0.686	0.96
Thiosulfate,oxidation,by,SOX,complex_soxY	0.010	0.015	0.647	0.083	0.261	0.702	0.97
Dissimilatory,sulfate,reduction_dsrA	0.010	0.016	0.613	0.000	0.283	0.718	0.99
Urea,metabolism_ureA	0.009	0.016	0.590	0.167	0.152	0.734	0.31
Calvin,Cycle_rbcL	0.008	0.014	0.534	0.083	0.174	0.746	0.88
Dissimilatory,sulfate,reduction_aprB	0.008	0.014	0.555	0.000	0.239	0.759	1.00
Dissimilatory,nitrate,reduction_nrfA	0.008	0.015	0.493	0.167	0.065	0.771	0.18
C1,metabolism,Methanogenesis,_hdrC	0.007	0.014	0.521	0.000	0.217	0.783	0.96
C1,metabolism,Methanogenesis,_ftr	0.007	0.015	0.479	0.167	0.044	0.795	0.10
Dissimilatory,sulfate,reduction_aprA	0.007	0.013	0.521	0.000	0.217	0.807	1.00
Denitrification_norB	0.007	0.014	0.508	0.083	0.152	0.818	0.75
C1,metabolism,Methanogenesis,_fwdC,,fmdC	0.007	0.014	0.482	0.167	0.044	0.830	0.03
Dissimilatory,sulfate,reduction_dsrB	0.007	0.014	0.483	0.000	0.196	0.841	0.99
Dissimilatory,nitrate,reduction_nirB	0.007	0.015	0.457	0.167	0.022	0.852	0.02
Fermentation_alcohol,dehydrogenase_exaA	0.007	0.014	0.480	0.083	0.130	0.863	0.65
Glycolysis_Pyruvate,kinase_pyk	0.006	0.014	0.418	0.917	0.913	0.873	0.44
Denitrification_norC	0.006	0.013	0.452	0.083	0.109	0.882	0.60
Denitrification_nirS	0.006	0.012	0.454	0.000	0.174	0.892	0.97
Thiosulfate,oxidation,by,SOX,complex_soxA	0.006	0.012	0.455	0.000	0.174	0.901	0.98
Nitrogen,fixation_nifH	0.006	0.013	0.413	0.000	0.152	0.910	0.91
Assimilatory,nitrate,reduction_nasA	0.005	0.013	0.422	0.083	0.087	0.919	0.53
Calvin,Cycle_rbcS	0.005	0.012	0.421	0.083	0.087	0.927	0.66
Urea,metabolism_ureB	0.005	0.013	0.390	0.083	0.065	0.936	0.19
Coenzyme,M,biosynthesis_comA	0.004	0.014	0.327	0.083	0.022	0.943	0.41
Denitrification_nosZ	0.004	0.012	0.343	0.000	0.109	0.950	0.87
Reductive,acetyl,CoA,pathway,,Wood,Ljungdahl,pathway,_cooS,,acsA	0.003	0.010	0.346	0.000	0.109	0.956	0.86
C1,metabolism,Methanogenesis,_mer	0.003	0.010	0.303	0.000	0.087	0.961	0.88
C1,metabolism,Methanogenesis,_fwdB,,fmdB	0.003	0.010	0.296	0.083	0.000	0.966	0.09
Denitrification_nirK	0.003	0.010	0.296	0.083	0.000	0.970	0.10

Nitrification,Denitrification,Dissimilatory,nitrate,reduction_narH,,narY,,nxrB	0.003	0.009	0.306	0.000	0.087	0.975	0.88
Denitrification,Dissimilatory,nitrate,reduction_napB	0.003	0.009	0.307	0.000	0.087	0.980	0.89
Nitrification,Denitrification,Dissimilatory,nitrate,reduction_narG,,narZ,,nxrA	0.003	0.009	0.306	0.000	0.087	0.984	0.89
Dicarboxylate,hydroxybutyrate,cycle,Hydroxypropionate,hydroxybutylate,cycle_enoyl ,CoA,hydratase,,,3,hydroxyacyl,CoA,dehydrogenase	0.003	0.009	0.307	0.000	0.087	0.988	0.84
Assimilatory,nitrate,reduction_narB	0.002	0.008	0.262	0.000	0.065	0.992	0.80
Thiosulfate,oxidation,by,SOX,complex_soxX	0.002	0.008	0.262	0.000	0.065	0.995	0.78
Denitrification,Dissimilatory,nitrate,reduction_narI,,narV	0.002	0.008	0.262	0.000	0.065	0.999	0.86
Dissimilatory,nitrate,reduction_nirD	0.001	0.005	0.148	0	0.022	1.000	0.62

Supplementary Table S9 Number of non-redundant KOs and module completeness based on KEGG annotation of high-quality MAGs.

**Module**	**Description**	**Acidobacteria;Thermoanaerobaculia;FEB-10_sp.003105185_(MAG_50)**	**Bacteroidetes;Flavobacteriia;Maribacter_(MAG_45)**	**Proteobacteria;Alphaproteobacteria;Filomicrobium_sp.001516065_(MAG_36)**
	Central metabolism (map00010]			
M00001	Glycolysis (Embden-Meyerhof pathway), glucose => pyruvate	(10) (2 blocks missing 7/9)	(12) (complete 9/9)	(8) (2 blocks missing 7/9)
M00002	Glycolysis, core module involving three-carbon compounds	(5) (2 blocks missing 3/5)	(6) (complete 5/5)	(4) (2 blocks missing 3/5)
M00003	Gluconeogenesis, oxaloacetate => fructose-6P	(8) (2 blocks missing 5/7)	(8) (complete 7/7)	(5) (2 blocks missing 5/7)
M00307	Gluconeogenesis_Pyruvate oxidation, pyruvate => acetyl-CoA	(4) (complete 1/1)	(5) (complete 1/1)	(4) (complete 1/1)
	Carbon metabolism/autotrophic pathways (map01200)			
M00165	Reductive pentose phosphate cycle (Calvin cycle)	(3) (incomplete 3/11)	(6) (incomplete 8/11)	(7) (incomplete 8/11)
M00374				(8, 1) (incomplete 3/13,
M00375	Dicarboxylate-hydroxybutyrate	(9,2) (incomplete 6/13, 2/14)	(8,2) (incomplete 6/13 ,2/14)	1/14)
M00377	Reductive acetyl-CoA pathway (Wood-Ljungdahl pathway)	absent	(2) (incomplete 2/7)	(1) (incomplete 1/7)
	Methane metabolism (map0680)			
M00356	Methanogenesis, methanol => methane	(3) (2 blocks missing 1/3)	absent	absent
M00357	Methanogenesis, acetate => methane	(6) (incomplete 2/5)	(2) (incomplete 1/5)	absent
M00358	Coenzyme M biosynthesis	absent	absent	absent
M00563	Methanogenesis, methylamine/dimethylamine/trimethylamine => methane	(4) (incomplete 1/4)	absent	absent
M00567	Methanogenesis, CO2 => methane	(3) (incomplete 1/8)	absent	(3) (incomplete 1/8)
	Nitrogen metabolism (map00910)			
M00175	Nitrogen fixation, nitrogen => ammonia	absent	absent	(1) (incomplete 1/4)
M00529	Denitrification, nitrate => nitrogen	(1) (incomplete 1/4)	(1) (incomplete 1/4)	(3) (incomplete 1/4)
M00530	Dissimilatory nitrate reduction, nitrate => ammonia	(1) (1 block missing 1/2)	absent	(2) (1 block missing 1/2)
M00804	Complete nitrification, comammox, ammonia => nitrite => nitrate	absent	absent	absent
M00531	Assimilatory nitrate reduction, nitrate => ammonia	absent	absent	(4) (complete 2/2)
	Sulfur metabolism (map00920)			
M00596	Dissimilatory sulfate reduction	(5) (complete 3/3)	absent	absent
M00595	Thiosulfate oxidation by SOX complex	absent	absent	(6) (1 block missing 6/7)

**Module**	**Description**	**Proteobacteria;Alphaproteobacteria;Pelagibius_litoralis_(MAG_39)**	**Proteobacteria;AlphaproteobacteriaHyphomonas_(MAG_47)**	**Proteobacteria;DeltaproteobacteriaDesulfosarcina_(MAG_11)**
	Central metabolism (map00010]			
M00001	Glycolysis (Embden-Meyerhof pathway), glucose => pyruvate	(10) (complete 9/9)	(9) (1 block missing 8/9)	(10) (1 block missing 8/9)
M00002	Glycolysis, core module involving three-carbon compounds	(6) (complete 5/5)	(6) (complete 5/5)	(6) (1 block missing 4/5)
M00003	Gluconeogenesis, oxaloacetate => fructose-6P	(8) (complete 7/7)	(7) (1 block missing 6/7)	(7) (2 blocks missing 5/7)

M00307	Gluconeogenesis_Pyruvate oxidation, pyruvate => acetyl-CoA	(5) (complete 1/1)	(4) (complete 1/1)	(8) (complete 1/1)
	Carbon metabolism/autotrophic pathways (map01200)			
M00165	Reductive pentose phosphate cycle (Calvin cycle)	(5) (incomplete 6/11)	(6) (2 blocks missing 9/11)	(5) (incomplete 7/11)
M00374 M00375	Dicarboxylate-hydroxybutyrate	(9,3) (incomplete 5/13, 2/14)	(6,2) (incomplete 4/13, 2/14)	(10,5) (incomplete 6/13, 4/14)
M00377	Reductive acetyl-CoA pathway (Wood-Ljungdahl pathway)	(2) (incomplete 2/7)	(2) (incomplete 2/7)	(8) (incomplete 4/7)
	Methane metabolism (map0680)			
M00356	Methanogenesis, methanol => methane	absent	absent	(4) (2 blocks missing 1/3)
M00357	Methanogenesis, acetate => methane	(3) (incomplete 1/5)	(1) (incomplete 1/5)	(8) (2 blocks missing 3/5)
M00358	Coenzyme M biosynthesis	absent	absent	(1) (incomplete 1/4)
M00563	Methanogenesis, methylamine/dimethylamine/trimethylamine => methane	(4) (incomplete 1/4)	absent	(5) (incomplete 1/4)
M00567	Methanogenesis, CO2 => methane	(3) (incomplete 1/8)	absent	(4) (incomplete 1/8)
	Nitrogen metabolism (map00910)			
M00175	Nitrogen fixation, nitrogen => ammonia	absent	absent	absent
M00529	Denitrification, nitrate => nitrogen	absent	absent	(4) (incomplete 1/4)
M00530	Dissimilatory nitrate reduction, nitrate => ammonia	absent	absent	(4) (1 block missing 1/2)
M00804	Complete nitrification, comammox, ammonia => nitrite => nitrate	absent	absent	(2) (2 blocks missing 1/3)
M00531	Assimilatory nitrate reduction, nitrate => ammonia	absent	absent	(1) (1 block missing 1/2)
	Sulfur metabolism (map00920)			
M00596	Dissimilatory sulfate reduction	absent	absent	(2) (1 block missing 2/3)
M00595	Thiosulfate oxidation by SOX complex	absent	absent	absent

**Module**	**Description**	**Proteobacteria;Gammaproteobacteria;Sedimenticolaceae_(MAG_28)**	**Proteobacteria;Gammaproteobacteria;Halioglobus_sp.009937575_(MAG_32)**	**Proteobacteria;Gammaproteobacteria;UBA9214_(MAG_33)**
	Central metabolism (map00010]			
M00001	Glycolysis (Embden-Meyerhof pathway), glucose => pyruvate	(7) (2 blocks missing 7/9)	(10) (complete 9/9)	(12) (1 block missing 8/9)
M00002	Glycolysis, core module involving three-carbon compounds	(4) (2 blocks missing 3/5)	(6) (complete 5/5)	(8) (complete 5/5)
M00003	Gluconeogenesis, oxaloacetate => fructose-6P	(5) (2 blocks missing 5/7)	(7) (1 block missing 6/7)	(9) (2 blocks missing 5/7)
M00307	Gluconeogenesis_Pyruvate oxidation, pyruvate => acetyl-CoA	(3) (complete 1/1)	(7) (complete 1/1)	(8) (complete 1/1)
	Carbon metabolism/autotrophic pathways (map01200)			
M00165	Reductive pentose phosphate cycle (Calvin cycle)	(3) (incomplete 3/11)	(5) (incomplete 7/11)	(7) (incomplete 8/11)
M00374 M00375	Dicarboxylate-hydroxybutyrate	(5,1) (incomplete 3/13, 1/14)	(11,2) (incomplete 5/13, 2/14)	(13,1) (incomplete 7/13, 1/14)
M00377	Reductive acetyl-CoA pathway (Wood-Ljungdahl pathway)	(2) (incomplete 2/7)	(3) (incomplete 3/7)	(2) (incomplete 2/7)
	Methane metabolism (map0680)			
M00356	Methanogenesis, methanol => methane	absent	absent	absent
M00357	Methanogenesis, acetate => methane	(1) (incomplete 1/5)	(1) (incomplete 1/5)	(2) (incomplete 1/5)
M00358	Coenzyme M biosynthesis	absent	absent	absent
M00563	Methanogenesis, methylamine/dimethylamine/trimethylamine => methane	absent	absent	absent
M00567	Methanogenesis, CO2 => methane	absent	absent	absent
	Nitrogen metabolism (map00910)			
M00175	Nitrogen fixation, nitrogen => ammonia	absent	absent	absent
M00529	Denitrification, nitrate => nitrogen	absent	absent	absent
M00530	Dissimilatory nitrate reduction, nitrate => ammonia	absent	absent	(4) (1 block missing 1/2)

M00804	Complete nitrification, comammox, ammonia => nitrite => nitrate	absent	absent	absent
M00531	Assimilatory nitrate reduction, nitrate => ammonia	absent	absent	(3) (complete 2/2)
	Sulfur metabolism (map00920)			
M00596	Dissimilatory sulfate reduction	(3) (1 block missing 2/3)	absent	(4) (1 block missing 2/3)
M00595	Thiosulfate oxidation by SOX complex	absent	absent	(6) (1 block missing 6/7)
